# The Health Education Research Experience (HERE) program metadata dataset

**DOI:** 10.1016/j.dib.2020.105180

**Published:** 2020-01-25

**Authors:** Mindy Menn, Caroline Payne-Purvis, Julia Alber, J. Don Chaney, Beth H. Chaney, Michael Stellefson, Suzanne Sneed-Murphy

**Affiliations:** a Texas Woman's University, USA; b Mississippi University for Women, USA; c California Polytechnic State University, USA; d East Carolina University, USA; e University of Florida, USA

**Keywords:** College students, Health education, Health promotion, Metadata, Online education, Public health, Questionnaire design, Survey methodology

## Abstract

Undergraduate subject pools are prevalent across disciplines in the United States. The Health Education Research Experience (HERE) Program was the first known course-based subject pool entirely managed and conducted online for online students enrolled in an introductory health education/health promotion course. The program was conducted within five semesters from Spring 2012 through Summer 2013. The HERE Program encompassed 13 studies embedded in two sections of an undergraduate online course at the University of Florida. The studies were all related to course topics and current research topics in health education/promotion (as identified through the Healthy People 2020 Framework). The topics ranged from the relatively less sensitive health aspects of college life (i.e., technology use) to studies assessing more sensitive health topics (i.e., intimate partner violence and sexual assault). In alignment with a best practice in survey design, the HERE Program's survey instruments included one metadata item embedded in each survey to identify which devices students used to complete the surveys. Understanding which devices students used for survey completion has ramifications for survey designers and survey researchers. In contrast to the relative uniformity of pen and paper surveys and control of the survey completion environment, online surveys may not look identical across personal devices and may be completed in increasingly varied environments. All studies, study procedures and protocols, and metadata collection procedures were approved by the university's Institutional Review Board. The data presented here were extracted from each survey's data files and aggregated. The aggregated metadata are available through Mendeley Data in a.csv file for widespread access. Descriptive statistics are presented in tables. The data provided in this article will benefit researchers interested in survey methodology, questionnaire design, modes of survey collection, and survey metadata. The data are hosted in the following Mendeley Data repository: https://data.mendeley.com/datasets/ht9jmd3cdt/2.

**Table:** Specifications Table

Subject	Social Sciences: Education
Specific subject area	Health Education/Health Promotion
Type of data	Tables, database
How data were acquired	The metadata were automatically recorded by Qualtrics survey software upon each participant accessing a survey and responding to the informed consent item. The metadata and select demographic characteristics were extracted from the individual datasets and aggregated into one.csv spreadsheet. The demographic data were self-reported by participants through individual survey responses. As noted, informed consent was obtained from all participants and 12 of the 13 survey instruments (with the approved Informed Consent language) are included as supplemental files. PI and Co-PI information was redacted from the files as their contact information is updated from the time of survey administration.
Data format	Raw and Analyzed
Parameters for data collection	Participants were recruited from two sections of one online-delivered health education/health promotion course at a large university in the United States. Participants included students representing all undergraduate academic classifications (freshman, sophomore, junior, and senior). Professional students and graduate students taking the course as an elective or for professional development were not the focus of this program and comprised 1.3% (n = 136) of the sample.
Description of data collection	The metadata presented in this article were automatically collected by Qualtrics survey software. Raw metadata and select demographic characteristics from 41 data collection points were extracted and aggregated in a.csv file. The IRB-approved metadata-related language is included in the Informed Consent section of each survey instrument file.
Data source location	Institution: University of Florida City/Town/Region: Gainesville, Florida Country: United States of America Latitude and longitude (and GPS coordinates) for collected samples/data: 29.6436° N, 82.3549° W
Data accessibility	Repository name: Mendeley Data Data identification number: [10.17632/ht9jmd3cdt.1] Direct URL to data: https://data.mendeley.com/datasets/ht9jmd3cdt/2

**Table:** 

**Value of the Data** • The data will be useful for researchers interested in comparing survey response patterns between desktop computers and mobile devices. • Survey research is prolific in health education/promotion as researchers strive to better understand human behavior. These data include metadata aggregated from adult college students participating in multiple health education studies at an American university. • These data will be valuable to researchers who want to examine relationships between a health topic's sensitivity, participants' device use patterns, and survey completion patterns. • These data will be useful to researchers examining relationships between devices used to complete online surveys and time elapsed to survey completion.

## 1 Data

Online delivered surveys are prolific social science research tools as adults in the United States are increasingly connected to internet-enabled devices [1]. Consequently, adults of all ages are able to complete surveys on more devices in more places than previous generations of survey participants. From campus computer labs, to personal laptops, internet-enabled smartphones and tablet computers, most undergraduate students have multiple options from which to choose when deciding where, when, and how to complete an online survey. Researchers have extensively examined the differences between pen and paper and online surveys. Yet comparably fewer researchers have examined the differences (and ramifications) between completing an online survey on a desktop computer or a mobile device [[2], [3], [4]]. Knowing which devices college students used to complete online surveys is important to understanding response patterns and developing more efficient and effective online survey instruments and ultimately improve data quality [5]. Consequently, the focus of this dataset is on the metadata collected from participants in the Health Education Research Experience program.

The 10,808 individual survey responses were obtained through 13 surveys conducted in five semesters across two years (Spring 2012, Summer 2012, Fall 2012, Spring 2013, Summer 2013). From this total, the authors removed 494 cases for the following reasons: two cases were identified as survey previews, three cases were identified as spam, 478 did not collect metadata, five did not consent to participate in a specific study, and six did not consent to participate due to previous participation (perhaps from taking the course in a previous semester). The final dataset contains descriptive survey information, participants' self-identified sex and academic classification, and metadata aggregated from 10,314 survey responses. The raw data are available in a widely accessible.csv file. A PDF codebook is included as a supplementary file.

The target population included all undergraduate students enrolled in an introductory health education/health promotion course at a large university in the southeastern United States across five semesters. Table 1 includes the survey titles as approved by the university's institutional review board (IRB), the semester(s) in which students completed the surveys for course credit, and the cumulative number of students enrolled in the course across two sections each semester as identified on the official university census dates.

**Table 1:** Survey description.

Survey Title	Semesters Conducted and Enrollment				
	Spring 2012 (n = 352)	Summer 2012 (n = 68)	Fall 2012 (n = 431)	Spring 2013 (n = 298)	Summer 2013 (n = 65)
Beliefs and Behaviors Related to Obesity and Mobile App and Internet Use				X	
Eating Attitudes and Objectification	X	X	X	X	X
Emergency Notification Systems: A Baseline Study			X	X	X
Emerging Issues in Injury Prevention: Firearm Accessibility and Intimate Partner Violence	X	X	X	X	
The Field of Health Education	X	X	X	X	
Geriatric Attitudes and Knowledge			X	X	
Hookah Smoking Knowledge, Attitudes, and Behaviors of College Students				X	X
HPV and Men	X	X	X	X	
Mental Health-Related Information Sources and College Students	X	X	X	X	
Obesity Campaigns			X		
Survey on Distracted Driving Attitudes and Behaviors	X	X	X	X	X
Technology, Health, and College Students	X	X	X	X	
University Student Perceptions and Behaviors Regarding Nutrition Facts Labels	X	X	X		

Table 2 includes descriptive statistics of the demographic data and metadata.

**Table 2:** Demographic and metadata characteristics.

Characteristic	*n* (%)
**Sex**	
Female	7499 (73.9)
Male	2634 (26.0)
Intersex	14 (0.1)
User Missing	167 (1.6)
Total	10,314 (100)
**Academic Classification**	
Freshman	1192 (11.7)
Sophomore	2710 (26.6)
Junior	2692 (26.4)
Senior	2353 (23.1)
Graduate/Professional/Non-Degree Seeking	136 (1.3)
Omitted from survey (Not Collected)	1097 (10.8)
User Missing	134 (1.3)
Total	10,314 (100)
**Finished Survey Status**	
Yes	10,181 (98.7)
No	133 (1.3)
Total	10,314 (100)
**Browser**	
Chrome	3303 (32.0)
Mozilla/Firefox	2712 (26.3)
MSIE	1553 (15.1)
Opera	11 (0.1)
Safari (including iPad, iPhone, iPod)	2735 (26.5)
Total	10,314 (100)
**Operating System**	
Android	16 (.2)
Linux	8 (.1)
iOS (iPhone, iPad, and iPod)	207 (2.0)
Macintosh	4397 (42.6)
Windows	5499 (53.3)
Unknown	187 (1.8)
Total	10,314 (100)
**Screen Resolution**	
1440 × 900	739 (7.2)
1366 × 768	3072 (29.8)
1280 × 800	4010 (38.9)
1280 × 1024	564 (5.5)
Other Resolutions	1929 (18.6)
Total	10,314 (100)

## 2 Experimental design, materials, and methods

Data include survey information provided by the authors (the semester of survey participation, the year of survey participation, and the official title of each IRB-approved study). The metadata include Qualtrics-generated survey information (Start Date, End Date, Recorded Date, Duration (in seconds) to complete the survey, and Finished status (0 = No and 1 = Yes), and device-related metadata. The device-related metadata included the participants' browser type, browser version, operating system, and screen resolution (in pixels). Demographic information provided by the participants (participants' self-identified sex and academic classification) were also included. These two variables were included because they were included across most of the HERE Program studies. A PDF codebook is included as a supplementary file.

All studies were conducted in Qualtrics survey software. In order to collect the device-related metadata, one Meta Info question was embedded on the informed consent landing page of each survey instrument. This placement was hidden to participants (yet acknowledged in the informed consent and approved by the institution's IRB) and ensured that metadata were automatically collected from all devices participants used to access surveys and respond to the informed consent item.

According to Qualtrics, the Meta Info item automatically collects a participants':

“Browser: The browser the respondent is using (e.g., Chrome or Internet Explorer). Version: The version of the browser the respondent is using. Operating System: The operating system the respondent is using (e.g., Windows or Macintosh). Screen Resolution: The size of the respondent's computer screen (in pixels)” [6].

The Meta Info item was standardized across all instruments into which it was inserted and the item could not be modified. As the metadata item was not shown to any participant (and therefore the participants could not verify the recorded data nor choose to skip the item or omit specific device specifications), the authors consider the approved informed consent language to be an especially noteworthy contribution of this research.
