# Supplementary material for: The Health Education Research Experience (HERE) program metadata dataset
Source: Data Brief. 2020 Jan 25;29:105180. doi: 10.1016/j.dib.2020.105180 (PMC7100622; doi:10.1016/j.dib.2020.105180)
Supplement: Multimedia component 1 [file mmc1.pdf]

Eating Attitudes and Objectification

Protocol Title: Eating Attitudes and Objectification
Please read this consent document carefully before you decide to participate in this study.

Purpose of the research study:
The purpose of the current study is to examine the attitudes towards eating and eating behaviors among college students related to objectification experiences.

Role of Research in HSC 3102:
One of the primary responsibilities of Certified Health Education Specialists is to *Conduct Evaluation and Research Related to Health Education*. As such, one of the goals of HSC 3102 – Personal and Family Health -- is to familiarize you with the research process in health education. To familiarize you with the research process in health education, we have created online surveys and introspective journal entries related to the content in each module.

Earning Health Education Research Experience Points:
This module includes a survey AND a journal entry. For this module, you may choose to participate in EITHER activity to receive your Health Education Research Experience points (5 points). Deadlines for this module's survey participation or journal entry are listed in the Sakai course website and correspond with the deadline for completing this module.

What you will be asked to do in the study:
You will be asked to answer 73 questions on an online survey using Qualtrics regarding your objectification experiences and eating attitudes. You will be asked to provide demographic information but will not be asked or required to provide personal identification information. We are also interested in how you complete this survey (e.g. on your computer, your phone, or a tablet computer like an IPad). As such, the survey program, Qualtrics, will collect technical information addressed in the Confidentiality Section below.

At the end of the survey, you will be directed to an external website which will collect your name and email address in order for the instructor to assign credit for participation in this study. If you choose to enter an email address in the external website form, you will receive a confirmation email for your records. If you choose to participate in the study and at the end of your participation you are not directed to the external website and/or do not receive a confirmation email please contact [redacted] as soon as you encounter the technical difficulty.

Confidentiality:
We will not connect your name or email address to your responses. Your information will be assigned a code number. The PI, Co-PI, and Supervisor will not log IP addresses, track IP addresses, or attach IP addresses to information. Your name will not be used in any report, presentation, or publication.

This survey contains a hidden item that collects information about your browser, browser version, operating system, screen resolution, flash version, java support version, and user agent from each device used to complete a survey. An example of the output created by Qualtrics for this item is below. (The output is the information that the researchers will be able to see when we analyze the results.)

Browser	Version	Operating System	Screen Resolution	Flash Version	Java Support	User Agent
Chrome	14.0.835.202	WOW64	1600x900	11.0.1	1	Mozilla/5.0 (Windows NT 6.1; WOW64) AppleWebKit/535.1 (KHTML, like Gecko) Chrome/14.0.835.202 Safari/535.1

This information identifies technical specifications of your device but cannot be used to identify you or your device.

Additional security:
The responses you provide are completely anonymous and cannot be connected with you at any time. The survey is delivered through Qualtrics. There is a minimal risk that security of any online data may be breached, but Qualtrics provides password protection (only the PI and Co-PI can access the data), hosts data on secure servers, and all results are firewall protected so it is highly unlikely that a security breach of the online data would occur or would result in an adverse consequence for you. The Qualtrics privacy statement can be located by clicking on the following link: http://www.qualtrics.com/privacy-statement

Time required:
Approximately 20-30 minutes

Risks and Benefits:
There are minimal risks associated with this study. We do not anticipate that you will benefit directly by participating in this research.

Compensation:
You will receive Health Education Research Experience participation credit for this module in HSC 3102. The participation credit for this module is five (5) points of your total course grade.

Voluntary participation:
Your participation in this study is completely voluntary. There is no penalty for not participating. If you prefer to complete the journal entry for this module instead of this research, please close this window, return to the 3102 course website in Sakai and access the instructions for the module's journal entry located in the corresponding module page under the Course Materials tab.

Right to withdraw from the study:
You have the right to withdraw from the study at anytime without consequence. You will still receive the participation credit (5 points) if you withdraw from the study before the conclusion of the survey. If you choose to participate in the

study and at the end of your participation you are not directed to the external website, please contact [redacted] as soon as you encounter the technical difficulty.

Whom to contact if you have questions about the study:
[redacted]

Whom to contact about your rights as a research participant in the study:
IRB02 Office, [redacted], University of Florida, Gainesville, FL 32611-2250; [redacted]

Agreement:
I have read the procedure described above. I voluntarily agree to participate in the study.

- ○ Begin survey (I consent to participating in this study)
- ○ I do not want to participate in this study
- ○ I have already participated in this study

Browser Meta Info

#EditSection, BrowserInfoExplanation#
Browser: **Chrome**
Version: **79.0.3945.88**
Operating System: **Windows NT 10.0**
Screen Resolution: **1280x1024**
Flash Version: **-1**
Java Support: **0**
User Agent: **Mozilla/5.0 (Windows NT 10.0; Win64; x64) AppleWebKit/537.36 (KHTML, like Gecko) Chrome/79.0.3945.88 Safari/537.36**

Block 2

Are you 18 years of age or older?

Yes	No
○	○

Default Question Block

Please select a response for each of the following statements.

Am terrified about being overweight.

Always	Usually	Often	Sometimes	Rarely	Never
○	○	○	○	○	○

Avoid eating when I am hungry.

Always	Usually	Often	Sometimes	Rarely	Never
○	○	○	○	○	○

Find myself preoccupied with food.

Always	Usually	Often	Sometimes	Rarely	Never
○	○	○	○	○	○

Have gone on eating binges where I feel that I may not be able to stop.

Always	Usually	Often	Sometimes	Rarely	Never
○	○	○	○	○	○

Cut my food into small pieces.

Always	Usually	Often	Sometimes	Rarely	Never
○	○	○	○	○	○

Aware of the calorie content of foods that I eat.

Always	Usually	Often	Sometimes	Rarely	Never
○	○	○	○	○	○

Particularly avoid food with a high carbohydrate content (i.e. bread, rice, potatoes, etc.)

Always	Usually	Often	Sometimes	Rarely	Never
○	○	○	○	○	○

Feel that others would prefer if I ate more.

Always	Usually	Often	Sometimes	Rarely	Never
○	○	○	○	○	○

Vomit after I have eaten.

Always	Usually	Often	Sometimes	Rarely	Never
○	○	○	○	○	○

Feel extremely guilty after eating.

Always	Usually	Often	Sometimes	Rarely	Never
○	○	○	○	○	○

Am preoccupied with a desire to be thinner.

Always	Usually	Often	Sometimes	Rarely	Never
○	○	○	○	○	○

Think about burning up calories when I exercise.

Always	Usually	Often	Sometimes	Rarely	Never
O	O	O	O	O	O

Other people think that I am too thin.

Always	Usually	Often	Sometimes	Rarely	Never
O	O	O	O	O	O

Am preoccupied with the thought of having fat on my body.

Always	Usually	Often	Sometimes	Rarely	Never
O	O	O	O	O	O

Take longer than others to eat my meals.

Always	Usually	Often	Sometimes	Rarely	Never
O	O	O	O	O	O

Avoid foods with sugar in them.

Always	Usually	Often	Sometimes	Rarely	Never
O	O	O	O	O	O

Eat diet foods.

Always	Usually	Often	Sometimes	Rarely	Never
O	O	O	O	O	O

Feel that food controls my life.

Always	Usually	Often	Sometimes	Rarely	Never
O	O	O	O	O	O

Display self-control around food.

Always	Usually	Often	Sometimes	Rarely	Never
O	O	O	O	O	O

Feel that others pressure me to eat.

Always	Usually	Often	Sometimes	Rarely	Never
O	O	O	O	O	O

Give too much time and thought to food.

Always	Usually	Often	Sometimes	Rarely	Never
O	O	O	O	O	O

Feel uncomfortable after eating sweets.

Always	Usually	Often	Sometimes	Rarely	Never
○	○	○	○	○	○

Engage in dieting behavior.

Always	Usually	Often	Sometimes	Rarely	Never
○	○	○	○	○	○

Like my stomach to be empty.

Always	Usually	Often	Sometimes	Rarely	Never
○	○	○	○	○	○

Have the impulse to vomit after meals.

Always	Usually	Often	Sometimes	Rarely	Never
○	○	○	○	○	○

Enjoy trying new rich foods.

Always	Usually	Often	Sometimes	Rarely	Never
○	○	○	○	○	○

In the past 6 months have you: Gone on eating binges where you feel that you may not be able to stop?*
*Defined as eating much more than most people would under the same circumstances and feeling that eating is out of control.

Never	Once a month or less	2-3 times a month	Once a week	2-6 times a week	Once a day or more
○	○	○	○	○	○

In the past 6 months have you: Ever made yourself sick (vomited) to control your weight or shape?

Never	Once a month or less	2-3 times a month	Once a week	2-6 times a week	Once a day or more
○	○	○	○	○	○

In the past 6 months have you: Ever used laxatives, diet pills or diuretics (water pills) to control your weight or shape?

Never	Once a month or less	2-3 times a month	Once a week	2-6 times a week	Once a day or more
○	○	○	○	○	○

In the past 6 months have you: Exercised more than 60 minutes a day to lose or to control your weight?

Never	Once a month or less	2-3 times a month	Once a week	2-6 times a week	Once a day or more
○	○	○	○	○	○

Lost 20 pounds or more in the past 6 months.

Yes	No
○	○

I rarely think about how I look:

1 Strongly Disagree	2	3	4 Neither Agree nor Disagree	5	6	7 Strongly Agree	N/A
○	○	○	○	○	○	○	○

I think it is more important that my clothes are comfortable than whether they look good on me.

1 Strongly Disagree	2	3	4 Neither Agree nor Disagree	5	6	7 Strongly Agree	N/A
○	○	○	○	○	○	○	○

I think more about how my body feels than how my body looks.

1 Strongly Disagree	2	3	4 Neither Agree nor Disagree	5	6	7 Strongly Agree	N/A
○	○	○	○	○	○	○	○

I rarely compare how I look with how other people look.

1 Strongly Disagree	2	3	4 Neither Agree nor Disagree	5	6	7 Strongly Agree	N/A
○	○	○	○	○	○	○	○

During the day, I think about how I look many times.

1 Strongly Disagree	2	3	4 Neither Agree nor Disagree	5	6	7 Strongly Agree	N/A
○	○	○	○	○	○	○	○

I often worry about whether the clothes I am wearing make me look good.

1 Strongly Disagree	2	3	4 Neither Agree nor Disagree	5	6	7 Strongly Agree	N/A
○	○	○	○	○	○	○	○

I rarely worry about how I look to other people.

1 Strongly Disagree	2	3	4 Neither Agree nor Disagree	5	6	7 Strongly Agree	N/A
○	○	○	○	○	○	○	○

I am more concerned with what my body can do than how it looks.

1 Strongly Disagree	2	3	4 Neither Agree nor Disagree	5	6	7 Strongly Agree	N/A
○	○	○	○	○	○	○	○

When I can't control my weight, I feel like something must be wrong with me.

1 Strongly Disagree	2	3	4 Neither Agree nor Disagree	5	6	7 Strongly Agree	N/A
○	○	○	○	○	○	○	○

I feel ashamed of myself when I haven't made the effort to look my best.

1 Strongly Disagree	2	3	4 Neither Agree nor Disagree	5	6	7 Strongly Agree	N/A
○	○	○	○	○	○	○	○

I feel like I must be a bad person when I don't look as good as I could.

1 Strongly Disagree	2	3	4 Neither Agree nor Disagree	5	6	7 Strongly Agree	N/A
○	○	○	○	○	○	○	○

I would be ashamed for people to know what I really weigh.

1 Strongly Disagree	2	3	4 Neither Agree nor Disagree	5	6	7 Strongly Agree	N/A
○	○	○	○	○	○	○	○

I never worry that something is wrong with me when I am not exercising as much as I should.

1 Strongly Disagree	2	3	4 Neither Agree nor Disagree	5	6	7 Strongly Agree	N/A
○	○	○	○	○	○	○	○

When I'm not exercising enough, I question whether I am a good enough person.

1 Strongly Disagree	2	3	4 Neither Agree nor Disagree	5	6	7 Strongly Agree	N/A
○	○	○	○	○	○	○	○

Even when I can't control my weight, I think I am an okay person.

1 Strongly Disagree	2	3	4 Neither Agree nor Disagree	5	6	7 Strongly Agree	N/A
○	○	○	○	○	○	○	○

When I'm not the size I think I should be I feel ashamed.

1 Strongly Disagree	2	3	4 Neither Agree nor Disagree	5	6	7 Strongly Agree	N/A
○	○	○	○	○	○	○	○

I think a person is pretty much stuck with the looks they are born with.

1 Strongly Disagree	2	3	4 Neither Agree nor Disagree	5	6	7 Strongly Agree	N/A
	○	○		○	○		○

A large part of being in shape is having that kind of body in the first place.

1 Strongly Disagree	2	3	4 Neither Agree nor Disagree	5	6	7 Strongly Agree	N/A

I think a person can look pretty much how they want to if they are willing to work at it.

1 Strongly Disagree	2	3	4 Neither Agree nor Disagree	5	6	7 Strongly Agree	N/A

I really don't think I have much control over how my body looks.

1 Strongly Disagree	2	3	4 Neither Agree nor Disagree	5	6	7 Strongly Agree	N/A

I think a person's weight is mostly determined by the genes they are born with.

1 Strongly Disagree	2	3	4 Neither Agree nor Disagree	5	6	7 Strongly Agree	N/A

It doesn't matter how hard I try to change my weight, it's probably always going to be about the same.

1 Strongly Disagree	2	3	4 Neither Agree nor Disagree	5	6	7 Strongly Agree	N/A

I can weigh what I'm suppose to when I try hard enough.

1 Strongly Disagree	2	3	4 Neither Agree nor Disagree	5	6	7 Strongly Agree	N/A

The shape you are in depends mostly on your genes.

1 Strongly Disagree	2	3	4 Neither Agree nor Disagree	5	6	7 Strongly Agree	N/A

Sex

- Male
- Female

Birth Date (mm/dd/yyyy)

Height

Current Weight (lbs) (Please enter a number. You do not need to include 'pounds', 'lbs', or any other letters after the number).

Highest Weight (lbs) (excluding pregnancy):

Lowest Adult Weight (lbs)

Ideal Weight (lbs)

What is your race?

- White/Caucasian
- African American
- Asian
- Native American
- Pacific Islander
- Other

What is your current UF GPA?

- 3.5-4.0
- 3.0-3.49
- 2.5-2.99
- 2.0-2.49
- 1.5-1.99
- Below 1.49

How would you classify your sexual orientation?

- Asexual
- Bisexual/Bi
- Heterosexual/Straight
- Homosexual/Gay/Lesbian/Queer
- Unsure

- Decline to answer

What is your current relationship status?

- Married
- In a committed relationship (with a steady partner)
- Single (not dating)
- Dating
- Divorced
- Widowed
- Separated
- ________ Other

What is your classification at the University of Florida?

- Freshman
- Sophomore
- Junior
- Senior
- Graduate Student
- Professional Student
- Non-degree seeking student
- I am not a student at the University of Florida

In which college is your current major?

- College of Agricultural and Life Sciences
- College of Business Administration
- College of Dentistry
- College of Design, Construction, and Planning
- College of Education
- College of Engineering
- College of Fine Arts
- College of Health and Human Performance
- College of Journalism and Communications
- College of Law
- College of Liberal Arts and Sciences
- College of Medicine
- College of Nursing
- College of Pharmacy
- College of Public Health and Health Professions
- College of Veterinary Medicine

Where do you currently live?

- On campus dormitory
- Off-campus dormitory
- Apartment
- House
- Other

Do you live with your parent(s) or guardian(s)?

- Yes
- No

Powered by Qualtrics
