# Supplementary material for: The Health Education Research Experience (HERE) program metadata dataset
Source: Data Brief. 2020 Jan 25;29:105180. doi: 10.1016/j.dib.2020.105180 (PMC7100622; doi:10.1016/j.dib.2020.105180)
Supplement: Multimedia component 2 [file mmc2.pdf]

Emergency Notification Systems: A Baseline Study

Informed Consent
Protocol Title: Emergency Notification Systems: A Baseline Study
Please read this consent document carefully before you decide to participate in this study.

Purpose of the research study:
The purpose of this study is to identify current Emergency Notification System (ENS) knowledge and perceptions among a sample of college students. More specifically, this study will serve as a baseline research effort to determine current students' perceptions of the UF Alert system which is the University of Florida's ENS. The results of this study will add to the bodies of emergency preparedness and student development literature and address how to best deliver emergency notifications to students at this university. We are also interested in how you complete this survey (e.g. on your computer, your phone, or a tablet computer like an iPad). As such, the survey program, Qualtrics, will collect technical information addressed in the Confidentiality Section below.

Role of Research in HSC 3102:
One of the primary responsibilities of Certified Health Education Specialists is to *Conduct Evaluation and Research Related to Health Education*. As such, one of the goals of HSC 3102 – Personal and Family Health -- is to familiarize you with the research process in health education. To familiarize you with the research process in health education, we have created online surveys and introspective journal entries related to the content in each module.

Earning Health Education Research Experience Points:
This module includes a survey AND a journal entry. For this module, you may choose to participate in EITHER activity to receive your Health Education Research Experience points (5 points). Deadlines for this module's survey participation or journal entry are listed in the Sakai course website and correspond with the deadline for completing this module.

What you will be asked to do in the study:
You will be asked to take a 34-item questionnaire online through Qualtrics. In this study you will be asked your opinions about the UF Alert Emergency Notification System. You will be asked to provide demographic information but will not be asked or required to provide personal identification information. The responses you provide are completely anonymous and cannot be connected with you at any time.

At the end of the survey, you will be directed to an external website which will collect your name and email address in order for the instructor to assign credit for participation in this study. If you choose to enter an email address in the external website form, you will receive a confirmation email for your records. If you choose to participate in the study and at the end of your participation you are not directed to the external website and/or do not receive a confirmation email, please contact [redacted] as soon as you encounter the technical difficulty.

Time required:
Approximately 20-30 minutes

Risks and Benefits:
There are minimal risks associated with this study. We do not anticipate that you will benefit directly by participating in this research.

Compensation:
You will receive Health Education Research Experience participation credit for this module in HSC 3102. The participation credit for this module is five (5) points of your total course grade.

Confidentiality:
We will not connect your name or email address to your responses. Your information will be assigned a code number. The PI, Co-PI, and Supervisor will not collect IP addresses, track IP addresses, or attach IP addresses to information. Your name will not be used in any report, presentation, or publication.

This survey contains a hidden item that collects information about your browser, browser version, operating system, screen resolution, flash version, java support version, and user agent from each device used to complete a survey. An example of the output created by Qualtrics for this item is below. (The output is the information that the researchers will be able to see when we analyze the results.)

Browser	Version	Operating System	Screen Resolution	Flash Version	Java Support	User Agent
Chrome	14.0.835.202	WOW64	1600x900	11.0.1	1	Mozilla/5.0 (Windows NT 6.1; WOW64) AppleWebKit/535.1 (KHTML, like Gecko) Chrome/14.0.835.202 Safari/535.1

This information identifies technical specifications of your device but cannot be used to identify you or your device.

Voluntary participation:
Your participation in this study is completely voluntary. There is no penalty for not participating. <u>You can decline to answer any questions or quit taking the survey at any time without any penalty from your current or any future instructor.</u> The survey software (Qualtrics) allows you to decline to answer any question to which you do not want to answer. The responses you provide are completely anonymous and cannot be connected with you at any time.

If you prefer to complete the journal entry for this module instead of this research, please close this window, return to the 3102 course website in Sakai and access the instructions for the module's journal entry located in the corresponding module page under the Course Materials tab.

Additional security:
The responses you provide are completely anonymous and cannot be connected with you at any time. The survey is delivered through Qualtrics. There is a minimal risk that security of any online data may be breached, but Qualtrics provides password protection (only the PI and Co-PI can access the data), hosts data on secure servers, and all results are firewall protected so it is highly unlikely that a security breach of the online data would occur or would result in an adverse consequence for you. The Qualtrics privacy statement can be located by clicking on the following link: http://www.qualtrics.com/privacy-statement

Right to withdraw from the study:

You have the right to withdraw from the study at anytime without consequence. You will still receive the participation credit (5 points) if you withdraw from the study before the conclusion of the survey. If you choose to participate in the study and at the end of your participation you are not directed to the external website, please contact [redacted] as soon as you encounter the technical difficulty.

Whom to contact if you have questions about the study:

[redacted]

Whom to contact about your rights as a research participant in the study:

IRB02 Office, [redacted], University of Florida, Gainesville, FL 32611-2250; [redacted]

Agreement:

I have read the procedure described above. I voluntarily agree to participate in the study.

- ○ Begin survey (I consent to participating in this study)
- ○ I do not want to participate in this study
- ○ I have already participated in this study

Browser Meta Info

#EditSection, BrowserInfoExplanation#
Browser: **Chrome**
Version: **79.0.3945.88**
Operating System: **Windows NT 10.0**
Screen Resolution: **1280x1024**
Flash Version: **-1**
Java Support: **0**
User Agent: **Mozilla/5.0 (Windows NT 10.0; Win64; x64) AppleWebKit/537.36 (KHTML, like Gecko) Chrome/79.0.3945.88 Safari/537.36**

Actual Use

Have you received and/or read at least one UF Alert message since you have been a student at UF?

- ○ Yes
- ○ No
- ○ I am not a student at the University of Florida

Have you received at least one UF Alert message through the following channels?

	Yes	No
Email	○	○
Text message	○	○
Twitter	○	○

	Yes	No
Facebook	O	O
	Yes	No
Classroom or laboratory telephone or speaker system	O	O
University of Florida webpage	O	O
RSS Feed	O	O
Outdoor speakers in Turlington Plaza	O	O
	Yes	No
Outdoor speakers in the Plaza of the Americas	O	O
Outdoor speakers in The Sun Terrace	O	O
Outdoor speakers around the Reitz Student Union lawn	O	O

Did you receive any of the following UF Alerts?

	Yes	No	I'm not sure
UF Alert Ref armed subject Shands Gainesville, Garage10. Officers clearing location. Please refer to http://www.ufl.edu for details.	O	O	O
(1 of 2) UF Alert Ref incident at Shands Gnvl in Garage10 armed Black male subject believed to have left the campus area Call 352-392-1111	O	O	O
(2 of 2) with info 2 of 3 message	O	O	O
(1 of 2) UF Alert Reference armed disturbance reported at Shands Gainesville in Garage 10. Two subjects, one black male armed with handgun.	O	O	O
(2 of 2) 1 of 2 messages	O	O	O
	Yes	No	I'm not sure
UF Alert Location of incident is Gar10 Shands Gainesville no injuries reported at this time.	O	O	O
UF Alert 2 subjects detained, 1 black male white shirt with hand gun at large. Report info to 352-392-1111	O	O	O

	Yes	No	I'm not sure
UF Alert Reported armed 2 males subj's unk. race at shands Gar.10 unknown intent. UPD on scene. Report info to 352-392-1111	O	O	O
UF Alert Reference the Bomb Threat at Shands UF the threat was a hoax and two are now in custody.	O	O	O
UF Alert Shands main campus No suspicious devices located Officers are clearing the area Call 352 392-1111 with any information	O	O	O

Did you respond to any of the following UF Alerts? (By respond we mean did you take any actions such as leaving campus, evacuating a building, avoiding an area of campus, contacting a law enforcement agency, or alerting other individuals to the notification).

	Yes	No	No, I did not know how to respond
UF Alert Ref armed subject Shands Gainesville, Garage10. Officers clearing location. Please refer to http://www.ufl.edu for details.	O	O	O
(1 of 2) UF Alert Ref incident at Shands Gnvl in Garage10 armed Black male subject believed to have left the campus area Call 352-392-1111	O	O	O
(2 of 2) with info 2 of 3 message	O	O	O
(1 of 2) UF Alert Reference armed disturbance reported at Shands Gainesville in Garage 10. Two subjects, one black male armed with handgun.	O	O	O
(2 of 2) 1 of 2 messages	O	O	O

	Yes	No	No, I did not know how to respond
UF Alert Location of incident is Gar10 Shands Gainesville no injuries reported at this time.	O	O	O
UF Alert 2 subjects detained, 1 black male white shirt with hand gun at large. Report info to 352-392-1111	O	O	O

	Yes	No	No, I did not know how to respond
UF Alert Reported armed 2 males subj's unk. race at shands Gar.10 unknown intent. UPD on scene. Report info to 352-392-1111	○	○	○
UF Alert Reference the Bomb Threat at Shands UF the threat was a hoax and two are now in custody.	○	○	○
UF Alert Shands main campus No suspicious devices located Officers are clearing the area Call 352 392-1111 with any information	○	○	○

Self-Efficacy

To what extent do you agree or disagree with the following statements?
If I received a UF Alert about an on campus ________________________, I would know how to respond.

	Strongly agree	Moderately agree	Somewhat agree	Neutral (neither disagree nor agree)	Somewhat disagree	Moderately disagree	Strongly disagree
Shelter-In-Place	○	○	○	○	○	○	○
Building Evacuation	○	○	○	○	○	○	○
Campus Evacuation	○	○	○	○	○	○	○
Chemical or Hazardous Material Spill	○	○	○	○	○	○	○
Missing Person	○	○	○	○	○	○	○

	Strongly agree	Moderately agree	Somewhat agree	Neutral (neither disagree nor agree)	Somewhat disagree	Moderately disagree	Strongly disagree
Suicide Threat or Attempt	○	○	○	○	○	○	○
Utility Failure	○	○	○	○	○	○	○
Bomb Threat	○	○	○	○	○	○	○
Fire	○	○	○	○	○	○	○
Gunman on Campus	○	○	○	○	○	○	○

	Strongly agree	Moderately agree	Somewhat agree	Neutral (neither disagree nor agree)	Somewhat disagree	Moderately disagree	Strongly disagree
Trespass Notice	○	○	○	○	○	○	○
Tornado	○	○	○	○	○	○	○

	Strongly agree	Moderately agree	Somewhat agree	Neutral (neither disagree nor agree)	Somewhat disagree	Moderately disagree	Strongly disagree
Inclement Weather Closing	○	○	○	○	○	○	○

Perceived Ease of Use

To what extent do you agree or disagree with the following statements?

	Strongly agree	Moderately agree	Somewhat agree	Neutral (neither disagree nor agree)	Somewhat disagree	Moderately disagree	Strongly disagree
I think that UF Alerts are easy to use.	○	○	○	○	○	○	○
I think that UF Alerts are clear and understandable.	○	○	○	○	○	○	○
Learning how to use the UF Alert system was easy for me.	○	○	○	○	○	○	○
The UF Alert system is easy to interact with.	○	○	○	○	○	○	○

Perceived Usefulness

To what extent do you agree or disagree with the following statements?

	Strongly agree	Moderately agree	Somewhat agree	Neutral (neither disagree nor agree)	Somewhat disagree	Moderately disagree	Strongly disagree
As a student at the University of Florida (UF), I find that UF Alerts are useful.	○	○	○	○	○	○	○
The UF Alert system enables me to access the most RECENT updates about emergency situations occurring on or near the University of Florida campus.	○	○	○	○	○	○	○
The UF Alert system enables me to access the most ACCURATE updates about emergency situations occurring on or near the University of Florida campus.	○	○	○	○	○	○	○

	Strongly agree	Moderately agree	Somewhat agree	Neutral (neither disagree nor agree)	Somewhat disagree	Moderately disagree	Strongly disagree
If I could, I would unsubscribe from the UF Alert email system.	○	○	○	○	○	○	○
The University of Florida needs an emergency notification system to inform students, faculty, and staff about emergency incidents.	○	○	○	○	○	○	○

Attitude

To what extent do you agree or disagree with the following statement?
Sending a UF Alert through each of the following channels a good idea.

	Strongly agree	Moderately agree	Somewhat agree	Neutral (neither disagree nor agree)	Somewhat disagree	Moderately disagree	Strongly disagree
The University of Florida homepage	○	○	○	○	○	○	○
Text messaging	○	○	○	○	○	○	○
Email	○	○	○	○	○	○	○
IP telephones and speakers (i.e. phones and speakers installed in classrooms)	○	○	○	○	○	○	○

	Strongly agree	Moderately agree	Somewhat agree	Neutral (neither disagree nor agree)	Somewhat disagree	Moderately disagree	Strongly disagree
Outdoor speakers in Turlington Plaza	○	○	○	○	○	○	○
Outdoor speakers in the Plaza of the Americas	○	○	○	○	○	○	○
Outdoor speakers in The Sun Terrace	○	○	○	○	○	○	○
Outdoor speakers around the Reitz Student Union lawn	○	○	○	○	○	○	○

	Strongly agree	Moderately agree	Somewhat agree	Neutral (neither disagree nor agree)	Somewhat disagree	Moderately disagree	Strongly disagree
Facebook	○	○	○	○	○	○	○
Twitter	○	○	○	○	○	○	○

	Strongly agree	Moderately agree	Somewhat agree	Neutral (neither disagree nor agree)	Somewhat disagree	Moderately disagree	Strongly disagree
RSS feed	○	○	○	○	○	○	○
University of Florida Rumor Control Hotline	○	○	○	○	○	○	○

Which of the following words reflect your perception of the UF Alert system? Select all that apply.

- ☐ Reliable
- ☐ Valuable
- ☐ Informative
- ☐ Clear
- ☐ Honest
- ☐ Useful
- ☐ Timely
- ☐ Efficient
- ☐ Straight-forward
- ☐ Important
- ☐ Detailed
- ☐ None of the above
- ☐ ________ Other

Which of the following words reflect your perception of the UF Alert system? Select all that apply.

- ☐ Worthless
- ☐ Uninformative
- ☐ Confusing
- ☐ Dishonest
- ☐ Unpleasant
- ☐ Annoying
- ☐ Unreliable
- ☐ Redundant
- ☐ Inefficient
- ☐ Scary
- ☐ Vague
- ☐ None of the above
- ☐ ________ Other

Behavioral Intention

To what extent do you agree or disagree with the following statements?

	Strongly agree	Moderately agree	Somewhat agree	Neutral (neither disagree nor agree)	Somewhat disagree	Moderately disagree	Strongly disagree
I intend to read all future UF Alert messages I receive.	○	○	○	○	○	○	○
I intend to "follow" UF Alerts on Twitter.	○	○	○	○	○	○	○
I intend to "like" UF Alerts on Facebook.	○	○	○	○	○	○	○
I intend to unsubscribe from UF Alert text messages.	○	○	○	○	○	○	○
I intend to ignore all future UF Alert messages.	○	○	○	○	○	○	○

Demographics

What additional methods could university officials use to provide emergency information to you while you are ON campus?

What additional methods could university officials use to provide emergency information to you while you are OFF campus?

What is the best method university officials could use to provide emergency information to you while you are ON campus?

What is the best method university officials could use to provide emergency information to you while you are OFF campus?

How could the University of Florida improve the UF Alert system?

What is your sex?

- ○ Male
- ○ Female
- ○ Intersex/Transexual/Genderqueer

Have you ever served on active duty in the U.S. Armed Forces, military Reserves, or National Guard? *Active Duty does not include training for the Reserves or National Guard, but DOES include activation, for example, for the Persian Gulf War.*

- ○ Yes, now on active duty
- ○ Yes, on active duty during the last 12 months, but not now
- ○ Yes, on active duty in the past, but not during the last 12 months
- ○ No, training for Reserves or National Guard only
- ○ No, never served in the military

Are you a member of a social fraternity or sorority?

- ○ Yes
- ○ No
- ○ I am in the process of pledging/rushing/recruitment this semester

What is your race? (One or more categories may be selected)

- ☐ White
- ☐ Black or African American
- ☐ American Indian or Alaska Native
- ☐ Asian Indian
- ☐ Chinese
- ☐ Filipino
- ☐ Japanese
- ☐ Korean
- ☐ Vietnamese
- ☐ Other Asian
- ☐ Native Hawaiian
- ☐ Guamanian or Chamorro
- ☐ Samoan
- ☐ Other Pacific Islander

Are you Hispanic, Latino/a, or Spanish Origin? (One or more categories may be selected)

- ☐ No, not of Hispanic, Latino/a, or Spanish origin
- ☐ Yes, Mexican, Mexican American, Chicano/a
- ☐ Yes, Puerto Rican
- ☐ Yes, Cuban
- ☐ Yes, Another Hispanic, Latino/a, or Spanish origin

How would you classify your sexual orientation?

- ○ Asexual
- ○ Bisexual/Bi

- Heterosexual/Straight
- Homosexual/Gay/Lesbian/Queer
- Unsure
- Decline to answer

What is your current relationship status?

- Married
- In a committed relationship (with a steady partner)
- Single (not dating)
- Dating
- Divorced
- Widowed
- Separated
- ____________ Other

What is your classification at the University of Florida?

- Freshman
- Sophomore
- Junior
- Senior
- Graduate Student
- Professional Student
- Non-degree seeking student
- I am not a student at the University of Florida

In which college is your current major?

- College of Agricultural and Life Sciences
- College of Business Administration
- College of Dentistry
- College of Design, Construction, and Planning
- College of Education
- College of Engineering
- College of Fine Arts
- College of Health and Human Performance
- College of Journalism and Communications
- College of Law
- College of Liberal Arts and Sciences
- College of Medicine
- College of Nursing
- College of Pharmacy
- College of Public Health and Health Professions
- College of Veterinary Medicine

Where do you currently live?

- On campus dormitory
- Off-campus dormitory
- Apartment
- House
- Other

Do you live with your parent(s) or guardian(s)?

- Yes
- No

Do you currently own any of the following?

	Yes	No
Cell phone with Internet capability (smart phone)	○	○
Cell phone without Internet capability	○	○
Desktop computer	○	○
Electronic book reader (e.g. Nook, Kindle)	○	○
Game console (e.g. X-Box, PlayStation, Nintendo Wii)	○	○
	Yes	No
Laptop computer	○	○
mp3 player (e.g. iPod)	○	○
Netbook computer	○	○
Portable gaming device (e.g. Sony PSP, Nintendo 3DS)	○	○
Tablet computer (e.g. iPad)	○	○

Do you have access to a high-speed Internet connection at your current residence?

- Yes
- No
- I'm not sure

Do you have an unlimited text message/SMS plan?

- Yes
- No
- I don't know

Approximately how many text/SMS messages do you send per day?

- 0-10
- 11-20
- 21-50
- More than 50
- I don't know

How many text/SMS messages do you receive per day?

- 0-10
- 11-20
- 21-50
- More than 50
- I don't know

Do you have any suggestions regarding how we can improve this survey?

Powered by Qualtrics
