# Supplementary material for: The Health Education Research Experience (HERE) program metadata dataset
Source: Data Brief. 2020 Jan 25;29:105180. doi: 10.1016/j.dib.2020.105180 (PMC7100622; doi:10.1016/j.dib.2020.105180)
Supplement: Multimedia component 3 [file mmc3.pdf]

[redacted]

Informed Consent
Protocol Title: Emerging Issues in Injury Prevention: Firearm Accessibility and Intimate Partner Violence
Please read this consent document carefully before you decide to participate in this study.

Purpose of the research study:
This study addresses university students' experiences with firearm accessibility and with intimate partner violence. The purpose of this study is to examine University of Florida students' experiences regarding firearm accessibility and intimate partner violence. This research will supplement the current health education literature and injury prevention literature regarding college students at American universities. We are also interested in how you complete this survey (e.g. on your computer, your phone, or a tablet computer like an iPad). As such, the survey program, Qualtrics, will collect technical information addressed in the Confidentiality Section below.

Role of Research in HSC 3102:
One of the primary responsibilities of Certified Health Education Specialists is to *Conduct Evaluation and Research Related to Health Education*. As such, one of the goals of HSC 3102 – Personal and Family Health -- is to familiarize you with the research process in health education. To familiarize you with the research process in health education, we have created online surveys and introspective journal entries related to the content in each module.

Earning Health Education Research Experience Points:
This module includes a survey AND a journal entry. For this module, you may choose to participate in EITHER activity to receive your Health Education Research Experience points (5 points). Deadlines for the this module's survey participation or journal entry are listed in the Sakai course website and correspond with the deadline for completing this module.

What you will be asked to do in the study:
You will be asked to take a 37-item questionnaire online through Qualtrics. In this study you will be asked about your experiences with firearm accessibility and with intimate partner violence. You will be asked to provide demographic information but will not be asked or required to provide personal identification information. The responses you provide are completely anonymous and cannot be connected with you at any time.

At the end of the survey, you will be directed to an external website which will collect your name and email address in order for the instructor to assign credit for participation in this study. If you choose to enter an email address in the external website form, you will receive a confirmation email for your records. If you choose to participate in the study and at the end of your participation you are not directed to the external website and/or do not receive a confirmation email, please contact [redacted] as soon as you encounter the technical difficulty.

Time required:
Approximately 20-30 minutes

Risks and Benefits:
There are minimal risks associated with this study. We do not anticipate that you will benefit directly by participating in this research.

Compensation:
You will receive Health Education Research Experience participation credit for this module in HSC 3102. The participation credit for this module is five (5) points of your total course grade.

Confidentiality:
We will not connect your name or email address to your responses. Your information will be assigned a code number. The PI, Co-PI, and Supervisor will not collect IP addresses, track IP addresses, or attach IP addresses to information. Your name will not be used in any report, presentation, or publication.

This survey contains a hidden item that collects information about your browser, browser version, operating system, screen resolution, flash version, java support version, and user agent from each device used to complete a survey. An example of the output created by Qualtrics for this item is below. (The output is the information that the researchers will be able to see when we analyze the results.)

Browser	Version	Operating System	Screen Resolution	Flash Version	Java Support	User Agent
Chrome	14.0.835.202	WOW64	1600x900	11.0.1	1	Mozilla/5.0 (Windows NT 6.1; WOW64) AppleWebKit/535.1 (KHTML, like Gecko) Chrome/14.0.835.202 Safari/535.1

This information identifies technical specifications of your device but cannot be used to identify you or your device.

Voluntary participation:
Your participation in this study is completely voluntary. There is no penalty for not participating. This survey contains several very sexually explicit terms, phrases, definitions, and questions that you may be uncomfortable reading or responding to. <u>You can decline to answer any questions or quit taking the survey at any time without any penalty from your current or any future instructor.</u> The survey software (Qualtrics) allows you to decline to answer any question to which you do not want to answer. The responses you provide are completely anonymous and cannot be connected with you at any time.

If you prefer to complete the journal entry for this module instead of this research, please close this window, return to the 3102 course website in Sakai and access the instructions for the module's journal entry located in the corresponding module page under the Course Materials tab.

Additional security:
The responses you provide are completely anonymous and cannot be connected with you at any time. The survey is delivered through Qualtrics. There is a minimal risk that security of any online data may be breached, but Qualtrics provides password protection (only the PI and Co-PI can access the data), hosts data on secure servers, and all results are firewall protected so it is highly unlikely that a security breach of the online data would occur or would result in an adverse consequence for you. The Qualtrics privacy statement can be located by clicking on the following link: http://www.qualtrics.com/privacy-statement

Right to withdraw from the study:
You have the right to withdraw from the study at anytime without consequence. You will still receive the participation credit (5 points) if you withdraw from the study before the conclusion of the survey. If you choose to participate in the study and at the end of your participation you are not directed to the external website, please contact [redacted] as soon as you encounter the technical difficulty.

Whom to contact if you have questions about the study:
[redacted]

Whom to contact about your rights as a research participant in the study:
IRB02 Office, [redacted] University of Florida, Gainesville, FL 32611-2250; [redacted].

Agreement:
I have read the procedure described above. I voluntarily agree to participate in the study.

- ○ Begin survey (I consent to participating in this study)
- ○ I do not want to participate in this study
- ○ I have already participated in this study

BRFSS (2004S21:firearms)

Browser Meta Info

#EditSection, BrowserInfoExplanation#
Browser: **Chrome**
Version: **79.0.3945.88**
Operating System: **Windows NT 10.0**
Screen Resolution: **1280x1024**
Flash Version: **-1**
Java Support: **0**
User Agent: **Mozilla/5.0 (Windows NT 10.0; Win64; x64) AppleWebKit/537.36 (KHTML, like Gecko) Chrome/79.0.3945.88 Safari/537.36**

The next questions are about firearms. We are asking these in a health survey because of our interest in firearm-related injuries. Please include weapons such as pistols, shotguns, and rifles; but not BB guns, starter pistols, or guns that cannot fire. Include those kept in a garage, outdoor storage area, or motor vehicle.

Are any firearms now kept in or around your home?

- Yes
- No
- I don't know

Are any of these firearms now loaded?

- Yes
- No
- I don't know

Are any of these loaded firearms also unlocked? By unlocked, we mean you do not need a key or combination to get the gun or to fire it. We don't count a safety as a lock.

- Yes
- No
- I don't know

Sexual Violence (BRFSS2007M17)

The next section will ask you some questions about different types of physical and/or sexual violence or other unwanted sexual experiences. This information will allow us to better understand the problem of violence and unwanted sexual contact and may help others in the future. This is a sensitive topic. Some people may feel uncomfortable with these questions. At the end of this section, phone numbers for organizations that can provide information and referral for these issues will be provided. Please keep in mind that you can skip any question you do not want to answer.

In the past 12 months, has anyone touched sexual parts of your body after you said or showed that you didn’t want them to, or without your consent (for example being groped or fondled)?

- Yes
- No
- I don't know
- Decline to answer

In the past 12 months, has anyone exposed you to unwanted sexual situations that did not involve physical touching? Examples include things like sexual harassment, someone exposing sexual parts of their body to you, being seen by a peeping Tom, or someone making you look at sexual photos or movies?

- Yes
- No
- I don't know
- Decline to answer

The next section will ask you questions about unwanted sex. Unwanted sex includes things like putting anything into your vagina ***[If female***], anus, or mouth or making you do these things to them after you said or showed that you didn't want to.

It includes times when you were unable to consent, for example, you were drunk or asleep, or you thought you would be hurt or punished if you refused.

Has anyone EVER had sex with you after you said or showed that you didn't want them to or without your consent?

- Yes
- No
- I don't know
- Decline to answer

Has this happened in the past 12 months?

- Yes
- No
- I don't know
- Decline to answer

Has anyone EVER ATTEMPTED to have sex with you after you said or showed that you didn't want to or without your consent, BUT SEX DID NOT OCCUR?

- Yes
- No
- I don't know
- Decline to answer

Has this happened in the past 12 months?

- Yes
- No
- I don't know
- Decline to answer

This topic may bring up past experiences that some people may wish to talk about. If you or someone you know would like to talk to a trained counselor, please call **1-800-656-HOPE (4673)**.

Intimate Partner Violence (BRFSS2007M18)

The next questions are about different types of violence in relationships with an intimate partner. Intimate partner means any current or former spouse, boyfriend, or girlfriend. Someone you were dating, or romantically or

sexually intimate with would also be considered an intimate partner. Please keep in mind that you can skip any question you do not want to answer.

Has an intimate partner EVER THREATENED you with physical violence? This includes threatening to hit, slap, push, kick, or hurt you in any way.

- Yes
- No
- I don't know
- Decline to answer

Has an intimate partner EVER ATTEMPTED physical violence against you? This includes times when they tried to hit, slap, push, kick, or otherwise hurt you, BUT THEY WERE NOT ABLE TO.

- Yes
- No
- I don't know
- Decline to answer

Has an intimate partner EVER hit, slapped, pushed, kicked, or hurt you in any way?

- Yes
- No
- I don't know
- Decline to answer

This topic may bring up past experiences that some people may wish to talk about. If you or someone you know would like to talk to a trained counselor, there is a toll-free and confidential intimate partner violence telephone hotline you can call. The number is **1- 800-799-SAFE (7233)**.

Demographics

Would you say that in general your health is---

- Excellent
- Very good
- Good
- Fair
- Poor

What is your age?

[ ]

What is your sex?

- ○ Male
- ○ Female

Considering all types of alcoholic beverages, how many times during the past 2 weeks did you have 5 or more drinks on one occasion?

[▾]

Considering all types of alcoholic beverages, how many times during the past 2 weeks did you have 4 or more drinks on one occasion?

- ○ 0
- ○ 1
- ○ 2
- ○ 3
- ○ 4
- ○ 5
- ○ 6
- ○ 7
- ○ 8
- ○ 9
- ○ 10 or more

What is your classification at the University of Florida?

- ○ Freshman
- ○ Sophomore
- ○ Junior
- ○ Senior
- ○ Graduate Student
- ○ Professional Student
- ○ Non-degree seeking student
- ○ I am not a student at the University of Florida

In which college is your current major?

- ○ College of Agricultural and Life Sciences
- ○ College of Business Administration
- ○ College of Dentistry
- ○ College of Design, Construction, and Planning
- ○ College of Education
- ○ College of Engineering

- College of Fine Arts
- College of Health and Human Performance
- College of Journalism and Communications
- College of Law
- College of Liberal Arts and Sciences
- College of Medicine
- College of Nursing
- College of Pharmacy
- College of Public Health and Health Professions
- College of Veterinary Medicine

How would you classify your sexual orientation?

- Asexual
- Bisexual/Bi
- Heterosexual/Straight
- Homosexual/Gay/Lesbian/Queer
- Unsure
- Decline to answer

What is your current relationship status?

- Married
- In a committed relationship (with a steady partner)
- Single (not dating)
- Dating
- Divorced
- Widowed
- Separated
- __________ Other

What is your current health insurance status?

- I am covered under my parents' insurance.
- I have health insurance through my job not associated with the University of Florida.
- I have health insurance through my spouse.
- I have health insurance through the University of Florida.
- I am not insured.
- I don't know.

Where do you currently live?

- On campus dormitory

- Off-campus dormitory
- Apartment
- House
- Other

What is your approximate grade point average (one decimal place)?

Are you a member of a social fraternity or sorority?

- Yes
- No
- I am in the process of pledging/rushing/recruitment this semester

Have you ever served on active duty in the U.S. Armed Forces, military Reserves, or National Guard? *Active Duty does not include training for the Reserves or National Guard, but DOES include activation, for example, for the Persian Gulf War.*

- Yes, now on active duty
- Yes, on active duty during the last 12 months, but not now
- Yes, on active duty in the past, but not during the last 12 months
- No, training for Reserves or National Guard only
- No, never served in the military

What is your race? (One or more categories may be selected)

- White
- Black or African American
- American Indian or Alaska Native
- Asian Indian
- Chinese
- Filipino
- Japanese
- Korean
- Vietnamese
- Other Asian
- Native Hawaiian
- Guamanian or Chamorro
- Samoan
- Other Pacific Islander

Are you Hispanic, Latino/a, or Spanish Origin? (One or more categories may be selected)

- ☐ No, not of Hispanic, Latino/a, or Spanish origin
- ☐ Yes, Mexican, Mexican American, Chicano/a
- ☐ Yes, Puerto Rican
- ☐ Yes, Cuban
- ☐ Yes, Another Hispanic, Latino/a, or Spanish origin

Do you have any comments regarding this survey or how we can improve this survey for future participants?

Powered by Qualtrics
