# Supplementary material for: The Health Education Research Experience (HERE) program metadata dataset
Source: Data Brief. 2020 Jan 25;29:105180. doi: 10.1016/j.dib.2020.105180 (PMC7100622; doi:10.1016/j.dib.2020.105180)
Supplement: Multimedia component 4 [file mmc4.pdf]

Geriatric Attitudes and Knowledge

Informed Consent
Protocol Title: Geriatric Attitudes and Knowledge
Please read this consent document carefully before you decide to participate in this study.

Purpose of the research study:
This study addresses how university students perceive older persons. The purpose of this study is to examine University of Florida students' attitudes and knowledge regarding geriatric-related care and experiences. This research will supplement the current health education literature and the growing body of aging literature. We are also interested in how you complete this survey (e.g. on your computer, your phone, or a tablet computer like an iPad). As such, the survey program, Qualtrics, will collect technical information addressed in the Confidentiality Section below.

Role of Research in HSC 3102:
One of the primary responsibilities of Certified Health Education Specialists is to *Conduct Evaluation and Research Related to Health Education*. As such, one of the goals of HSC 3102 – Personal and Family Health -- is to familiarize you with the research process in health education. To familiarize you with the research process in health education, we have created online surveys and introspective journal entries related to the content in each module.

Earning Health Education Research Experience Points:
This module includes a survey AND a journal entry. For this module, you may choose to participate in EITHER activity to receive your Health Education Research Experience points (5 points). Deadlines for this module's survey participation or journal entry are listed in the Sakai course website and correspond with the deadline for completing this module.

What you will be asked to do in the study:
You will be asked to take a 21-item questionnaire online through Qualtrics. In this study you will be asked about your attitudes and knowledge regarding various aspects of aging. You will be asked to provide demographic information but will not be asked or required to provide personal identification information.

At the end of the survey, you will be directed to an external participation website which will collect your name and email address in order for the instructor to assign credit for participation in this study. If you choose to enter an email address in the external website form, you will receive a confirmation email for your records. If you choose to participate in the study and at the end of your participation you are not directed to the external website and/or do not receive a confirmation email, please contact [redacted] as soon as you encounter the technical difficulty.

In order to remove any risk that the researchers or any others could re-connect your name to your responses (for example, by using time-date information) from this survey and the external participation website, we will access the participation list immediately upon closure of this Module. The researchers will remove all potentially identifying information, assign credit, and delete this information from our records and the Qualtrics server immediately after credit is entered into the grade book. The information collected within this survey instrument will be accessed and analyzed at a later time. With these security protections in place, it is very unlikely that a security breach would result in any adverse consequence for you, and anonymity will be maintained.

Time required:
Approximately 20-30 minutes

Risks and Benefits:
There are minimal risks associated with this study. We do not anticipate that you will benefit directly by participating in this research.

Compensation:
You will receive Health Education Research Experience participation credit for this module in HSC 3102. The participation credit for this module is five (5) points of your total course grade.

Confidentiality:
We will not connect your name or email address to your responses. Your information will be assigned a code number. The PI, Co-PI, and Supervisor will not track IP addresses or attach IP addresses to information. Your name will not be used in any report, presentation, or publication.

This survey contains a hidden item that collects information about your browser, browser version, operating system, screen resolution, flash version, java support version, and user agent from each device used to complete a survey. An example of the output created by Qualtrics for this item is below. (The output is the information that the researchers will be able to see when we analyze the results.)

Browser	Version	Operating System	Screen Resolution	Flash Version	Java Support	User Agent
Chrome	14.0.835.202	WOW64	1600x900	11.0.1	1	Mozilla/5.0 (Windows NT 6.1; WOW64) AppleWebKit/535.1 (KHTML, like Gecko) Chrome/14.0.835.202 Safari/535.1

This information identifies technical specifications of your device but cannot be used to identify you or your device.

Voluntary participation:
Your participation in this study is completely voluntary. There is no penalty for not participating. <u>You can decline to answer any questions or quit taking the survey at any time without any penalty from your current or any future instructor.</u> The survey software (Qualtrics) allows you to decline to answer any question to which you do not want to answer. The responses you provide are completely anonymous and cannot be connected with you at any time.

If you prefer to complete the journal entry for this module instead of this research, please close this window, return to the 3102 course website in Sakai and access the instructions for the module's journal entry located in the corresponding module page under the Course Materials tab.

Additional security information: This survey and the survey instrument used to collect information for assigning the HERE participation credit are both delivered through Qualtrics. Any information that could serve to reconnect the

two surveys, for instance information about when you took the surveys, and the order in which the surveys were taken by different individuals, will be deleted from both files prior to sending participation information to your instructor and prior to any analyses for research purposes.

There is a minimal risk that security of any online data may be breached, but Qualtrics provides password protection (only the PI and Co-PI can access the data), hosts data on secure servers, and all results are firewall protected so it is highly unlikely that a security breach of the online data would occur or would result in an adverse consequence for you. The Qualtrics privacy statement can be located by clicking on the following link: http://www.qualtrics.com/privacy-statement

Right to withdraw from the study:
You have the right to withdraw from the study at anytime without consequence. You will still receive the participation credit (5 points) if you withdraw from the study before the conclusion of the survey. If you choose to participate in the study and at the end of your participation you are not directed to the external website, please contact [redacted] as soon as you encounter the technical difficulty.

Whom to contact if you have questions about the study:
[redacted]

Whom to contact about your rights as a research participant in the study:
IRB02 [redacted] University of Florida, Gainesville, FL 32611-2250; [redacted].

Agreement:
I have read the procedure described above. I voluntarily agree to participate in the study.

- ◯ Begin survey (I consent to participating in this study)
- ◯ I do not want to participate in this study
- ◯ I have already participated in this study

Browser Meta Info

#EditSection, BrowserInfoExplanation#
Browser: **Chrome**
Version: **79.0.3945.88**
Operating System: **Windows NT 10.0**
Screen Resolution: **1280x1024**
Flash Version: **-1**
Java Support: **0**
User Agent: **Mozilla/5.0 (Windows NT 10.0; Win64; x64) AppleWebKit/537.36 (KHTML, like Gecko) Chrome/79.0.3945.88 Safari/537.36**

ATOP Kogan

To what extent do you agree or disagree with the following statements?

	Strongly agree	Agree	Slightly agree	Slightly disagree	Disagree	Strongly disagree

	Strongly agree	Agree	Slightly agree	Slightly disagree	Disagree	Strongly disagree
Most old people tend to let their homes become shabby and unattractive.	O	O	O	O	O	O
Most old people get set in their ways and are unable to change.	O	O	O	O	O	O
Most old people are irritable, grouchy, and unpleasant.	O	O	O	O	O	O
There is something different about most old people; it's hard to figure out what makes them tick.	O	O	O	O	O	O
Most old people make one feel ill at ease.	O	O	O	O	O	O
Most old people are constantly complaining about the behavior of the younger generation.	O	O	O	O	O	O
	Strongly agree	Agree	Slightly agree	Slightly disagree	Disagree	Strongly disagree
Most old people bore others by their insistence on talking about the "good old days".	O	O	O	O	O	O
Old people have too much power in business and politics.	O	O	O	O	O	O
There are a few exceptions, but in general most old people are pretty much alike.	O	O	O	O	O	O
It would probably be better is most old people lived in residential units with people of their own age.	O	O	O	O	O	O
Most old people should be more concerned with their personal appearance; they're too untidy.	O	O	O	O	O	O
If old people expect to be liked, their first step is to try to get rid of their irritating faults.	O	O	O	O	O	O
	Strongly agree	Agree	Slightly agree	Slightly disagree	Disagree	Strongly disagree
Most old people spend too much time prying into the affairs of others and giving unsought advice.	O	O	O	O	O	O

	Strongly agree	Agree	Slightly agree	Slightly disagree	Disagree	Strongly disagree
Most old people would prefer to quit work as soon as pensions or their children can support them.	O	O	O	O	O	O
In order to maintain a nice residential neighborhood, it would be best if too many old people did not live in it.	O	O	O	O	O	O
Most old people make excessive demands for love and reassurance.	O	O	O	O	O	O
It is foolish to claim that wisdom comes with old age.	O	O	O	O	O	O

To what extent do you agree or disagree with the following statements?

	Strongly agree	Agree	Slightly agree	Slightly disagree	Disagree	Strongly disagree
Most old people would prefer to continue working just as long as they possibly can rather than be dependent on anybody.	O	O	O	O	O	O
It would probably be better if most old people lived is residential units that also housed younger people.	O	O	O	O	O	O
Old people should have more power in business and politics.	O	O	O	O	O	O
Most old people are capable of adjustments when the situation demands it.	O	O	O	O	O	O
You can count on finding a nice residential neighborhood when there is a sizable number of old people living in it.	O	O	O	O	O	O
One seldom hears old people complaining about the behavior of the younger generation.	O	O	O	O	O	O

	Strongly agree	Agree	Slightly agree	Slightly disagree	Disagree	Strongly disagree
It is evident that most old people are very different from one another.	O	O	O	O	O	O
Most old people need no more love and reassurance than anyone else.	O	O	O	O	O	O

	Strongly agree	Agree	Slightly agree	Slightly disagree	Disagree	Strongly disagree
One of the most interesting and entertaining qualities of most old people is their accounts of their past experiences.	O	O	O	O	O	O
When you think about it, old people have the same faults as anybody else.	O	O	O	O	O	O
People grow wiser with the coming of old age.	O	O	O	O	O	O
Most old people are cheerful, agreeable, and good humored.	O	O	O	O	O	O
	Strongly agree	Agree	Slightly agree	Slightly disagree	Disagree	Strongly disagree
Most old people are really no different from anybody else; they're as easy to understand as younger people.	O	O	O	O	O	O
Most old people are very relaxing to be with.	O	O	O	O	O	O
Most old people seem to be quite clean and neat in their personal appearance.	O	O	O	O	O	O
Most old people can generally be counted on to maintain a clean, attractive home.	O	O	O	O	O	O
Most old people tend to keep to themselves and give advice only when asked.	O	O	O	O	O	O

Demographics

What is your sex?

- O Male
- O Female
- O Intersex/Transexual/Genderqueer

How often do you currently interact with someone over the age of 65?

- O Never
- O Less than Once a Month
- O Once a Month
- O 2-3 Times a Month
- O Once a Week
- O 2-3 Times a Week

- ○ Daily

Have you ever lived with an adult over 65 years old?

- ○ Yes
- ○ No
- ○ I'm not sure

Do you currently live with an adult over 65 years old?

- ○ Yes
- ○ No

Have you ever volunteered or worked with someone over the age of 65?

- ○ Yes
- ○ No

Have you ever been or are you currently a primary caretaker for someone over the age of 65? (This includes home-bound care, home health care, and hospitalized/medical care).

- ○ Yes
- ○ No

Are you a member of a social fraternity or sorority?

- ○ Yes
- ○ No
- ○ I am in the process of pledging/rushing/recruitment this semester

What is your race? (One or more categories may be selected)

- ☐ White
- ☐ Black or African American
- ☐ American Indian or Alaska Native
- ☐ Asian Indian
- ☐ Chinese
- ☐ Filipino
- ☐ Japanese
- ☐ Korean
- ☐ Vietnamese
- ☐ Other Asian
- ☐ Native Hawaiian
- ☐ Guamanian or Chamorro
- ☐ Samoan

- [ ] Other Pacific Islander

Are you Hispanic, Latino/a, or Spanish Origin? (One or more categories may be selected)

- [ ] No, not of Hispanic, Latino/a, or Spanish origin
- [ ] Yes, Mexican, Mexican American, Chicano/a
- [ ] Yes, Puerto Rican
- [ ] Yes, Cuban
- [ ] Yes, Another Hispanic, Latino/a, or Spanish origin

How would you classify your sexual orientation?

- ○ Asexual
- ○ Bisexual/Bi
- ○ Heterosexual/Straight
- ○ Homosexual/Gay/Lesbian/Queer
- ○ Unsure
- ○ Decline to answer

What is your current relationship status?

- ○ Married
- ○ In a committed relationship (with a steady partner)
- ○ Single (not dating)
- ○ Dating
- ○ Divorced
- ○ Widowed
- ○ Separated
- ○ ________ Other

What is your classification at the University of Florida?

- ○ Freshman
- ○ Sophomore
- ○ Junior
- ○ Senior
- ○ Graduate Student
- ○ Professional Student
- ○ Non-degree seeking student
- ○ I am not a student at the University of Florida

In which college is your current major?

- ○ College of Agricultural and Life Sciences
- ○ College of Business Administration

- College of Dentistry
- College of Design, Construction, and Planning
- College of Education
- College of Engineering
- College of Fine Arts
- College of Health and Human Performance
- College of Journalism and Communications
- College of Law
- College of Liberal Arts and Sciences
- College of Medicine
- College of Nursing
- College of Pharmacy
- College of Public Health and Health Professions
- College of Veterinary Medicine

How many courses in Health Education and Behavior have you completed at the University of Florida?

Are you a Health Education & Behavior major at the University of Florida?

- Yes
- No

Do you currently live with your parent(s) or guardian(s)?

- Yes
- No

Do you have any suggestions regarding how we can improve this survey?

Powered by Qualtrics
