# Supplementary material for: The Health Education Research Experience (HERE) program metadata dataset
Source: Data Brief. 2020 Jan 25;29:105180. doi: 10.1016/j.dib.2020.105180 (PMC7100622; doi:10.1016/j.dib.2020.105180)
Supplement: Multimedia component 5 [file mmc5.pdf]

Health Education Research Experience (HERE) Metadata Codebook

Semester

		Value	Count	Percent
Valid Values	0	Fall	4474	43.4%
	1	Spring	5122	49.7%
	2	Summer	718	7.0%

Year

		Value	Count	Percent
Valid Values	0	2012	7219	70.0%
	1	2013	3095	30.0%

SurveyTopic

		Value	Count	Percent
Valid Values	1	Nutrition Facts Labels	764	7.4%
	2	Obesity Campaigns	402	3.9%
	3	Emergency Notification Systems	777	7.5%
	4	Geriatric Attitudes and Knowledge	704	6.8%
	5	Hookah	355	3.4%
	6	Health Education/Promotion	739	7.2%
	7	Eating Attitudes and Objectification	1107	10.7%
	8	DistractedDriving	1097	10.6%
	9	Firearm Accessibility and Intimate Partner Violence	1026	9.9%

SurveyTopic

		Value	Count	Percent
	10	Technology, Health, and College Students	971	9.4%
	11	Mental Health	1060	10.3%
	12	HPV and Men	1028	10.0%
	13	Beliefs and Behaviors Related to Obesity and Mobile Apps and Internet Use	284	2.8%

Finished

		Value	Count	Percent
N	Valid	10314		
	Missing	0		
Labeled Values	0	No	133	1.3%
	1	Yes	10181	98.7%

InformedConsent

		Value	Count	Percent
Valid Values	0	Consent Given	10314	100.0%
	1	No Consent	0	0.0%
	2	Disqualified by prior participation	0	0.0%

Sex

		Value	Count	Percent
Valid Values	0	Female	7499	72.7%
	1	Male	2634	25.5%
	2	Intersex	14	0.1%
Missing Values	System		167	1.6%

AcademicClassification

		Value	Count	Percent
N	Valid	10180		
	Missing	134		
Labeled Values	0	Freshman	1192	11.6%
	1	Sophomore	2710	26.3%
	2	Junior	2692	26.1%
	3	Senior	2353	22.8%
	4	Graduate/Professional/Non-Degree Seeking Student	136	1.3%
	98	Not Collected	1097	10.6%
