# Supplementary material for: The Health Education Research Experience (HERE) program metadata dataset
Source: Data Brief. 2020 Jan 25;29:105180. doi: 10.1016/j.dib.2020.105180 (PMC7100622; doi:10.1016/j.dib.2020.105180)
Supplement: Multimedia component 6 [file mmc6.pdf]

HPV and Men

Q1.
Informed Consent
Protocol Title: HPV and Men
Please read this consent document carefully before you decide to participate in this study.

Purpose of the research study:
The purpose of this study is to identify human papillomavirus (HPV) related knowledge among a sample of college students. As this survey is about a sexually transmitted infection (STI), and individuals who engage in risky sexual practices (i.e. having unprotected sex, having sex with multiple partners) are at higher risk for contracting a STI, this survey will include several very sexually explicit terms, phrases, definitions, and questions that participants may feel uncomfortable reading. The purpose of these questions is to examine the relationship between HPV-related knowledge and risky sexual behaviors. The results of this study will add to the body of sexuality education literature and address how to best plan STI prevention programs for college students. We are also interested in how you complete this survey (e.g. on your computer, your phone, or a tablet computer like an iPad). As such, the survey program, Qualtrics, will collect technical information addressed in the Confidentiality Section below.

Role of Research in HSC 3102:
One of the primary responsibilities of Certified Health Education Specialists is to *Conduct Evaluation and Research Related to Health Education*. As such, one of the goals of HSC 3102 – Personal and Family Health -- is to familiarize you with the research process in health education. To familiarize you with the research process in health education, we have created online surveys and introspective journal entries related to the content in each module.

Earning Health Education Research Experience Points:
This module includes a survey AND a journal entry. For this module, you may choose to participate in EITHER activity to receive your Health Education Research Experience points (5 points). Deadlines for the this module's survey participation or journal entry are listed in the Sakai course website and correspond with the deadline for completing this module.

What you will be asked to do in the study:
You will be asked to take a 55-item questionnaire online through Qualtrics. In this study you will be asked your opinions about HPV and HPV vaccinations. You will be asked to provide demographic information but will not be asked or required to provide personal identification information. This survey contains several very sexually explicit terms, phrases, and definitions that you may be uncomfortable reading or responding to. <u>You can decline to answer any questions or quit taking the survey at any time without any penalty from your current or any future instructor.</u> The responses you provide are completely anonymous and cannot be connected with you at any time.

At the end of the survey, you will be directed to an external website which will collect your name and email address in order for the instructor to assign credit for participation in this study. If you choose to enter an email address in the external website form, you will receive a confirmation email for your records. If you choose to

participate in the study and at the end of your participation you are not directed to the external website and/or do not receive a confirmation email, please contact [redacted] as soon as you encounter the technical difficulty.

Time required:
Approximately 20-30 minutes

Risks and Benefits:
There are minimal risks associated with this study. We do not anticipate that you will benefit directly by participating in this research.

Compensation:
You will receive Health Education Research Experience participation credit for this module in HSC 3102. The participation credit for this module is five (5) points of your total course grade.

Confidentiality:
We will not connect your name or email address to your responses. Your information will be assigned a code number. The PI, Co-PI, and Supervisor will not collect IP addresses, track IP addresses, or attach IP addresses to information. Your name will not be used in any report, presentation, or publication.

This survey contains a hidden item that collects information about your browser, browser version, operating system, screen resolution, flash version, java support version, and user agent from each device used to complete a survey. An example of the output created by Qualtrics for this item is below. (The output is the information that the researchers will be able to see when we analyze the results.)

Browser	Version	Operating System	Screen Resolution	Flash Version	Java Support	User Agent
Chrome	14.0.835.202	WOW64	1600x900	11.0.1	1	Mozilla/5.0 (Windows NT 6.1; WOW64) AppleWebKit/535.1 (KHTML, like Gecko) Chrome/14.0.835.202 Safari/535.1

This information identifies technical specifications of your device but cannot be used to identify you or your device.

Voluntary participation:
Your participation in this study is completely voluntary. There is no penalty for not participating. This survey contains several very sexually explicit terms, phrases, definitions, and questions that you may be uncomfortable reading or responding to. <u>You can decline to answer any questions or quit taking the survey at any time without any penalty from your current or any future instructor.</u> The survey software (Qualtrics) allows you to decline to

answer any question to which you do not want to answer. The responses you provide are completely anonymous and cannot be connected with you at any time.

If you prefer to complete the journal entry for this module instead of this research, please close this window, return to the 3102 course website in Sakai and access the instructions for the module's journal entry located in the corresponding module page under the Course Materials tab.

Additional security:
The responses you provide are completely anonymous and cannot be connected with you at any time. The survey is delivered through Qualtrics. There is a minimal risk that security of any online data may be breached, but Qualtrics provides password protection (only the PI and Co-PI can access the data), hosts data on secure servers, and all results are firewall protected so it is highly unlikely that a security breach of the online data would occur or would result in an adverse consequence for you. The Qualtrics privacy statement can be located by clicking on the following link: http://www.qualtrics.com/privacy-statement

Right to withdraw from the study:
You have the right to withdraw from the study at anytime without consequence. You will still receive the participation credit (5 points) if you withdraw from the study before the conclusion of the survey. If you choose to participate in the study and at the end of your participation you are not directed to the external website, please contact [redacted] as soon as you encounter the technical difficulty.

Whom to contact if you have questions about the study:
[redacted]

Whom to contact about your rights as a research participant in the study:
IRB02 Office, [redacted], University of Florida, Gainesville, FL 32611-2250; [redacted].

Agreement:
I have read the procedure described above. I voluntarily agree to participate in the study.

- ○ Begin survey (I consent to participating in this study)
- ○ I do not want to participate in this study
- ○ I have already participated in this study

Q2. Browser Meta Info

#EditSection, BrowserInfoExplanation#
Browser: **Chrome**
Version: **79.0.3945.88**
Operating System: **Windows NT 10.0**
Screen Resolution: **1280x1024**
Flash Version: **-1**
Java Support: **0**
User Agent: **Mozilla/5.0 (Windows NT 10.0; Win64; x64) AppleWebKit/537.36 (KHTML, like Gecko) Chrome/79.0.3945.88 Safari/537.36**

Age Verification

Q3. Are you 18 years of age or older?

- Yes
- No

Informed Consent

Q4. Most sexually active people in the United States will have HPV at some time in their lives.

- True
- False
- I don't know

Q5. There is a cure for HPV.

- True
- False
- I don't know

Q6. Only women can get HPV.

- True
- False
- I don't know

Q7. HPV does not cause any symptoms.

- True
- False
- I don't know

Q8. Females between the ages of 9 and 26 can get the Gardasil vaccination series to protect against some types of HPV.

- True
- False
- I don't know

Q9. Females between the ages of 9 and 26 can get the Cervarix vaccination series to protect against some types of HPV.

- True
- False

- I don't know

Q10. Males between the ages of 9 and 26 can get the Gardasil vaccination series to protect against some types of HPV.

- True
- False
- I don't know

Q11. Males between the ages of 9 and 26 can get the Cervarix vaccination series to protect against some types of HPV.

- True
- False
- I don't know

Q12. Gardasil is available for females at the University of Florida Student Health Center.

- True
- False
- I don't know

Q13. Cervarix is available for females at the University of Florida Student Health Center.

- True
- False
- I don't know

Q14. Gardasil is available for males at the University of Florida Student Health Center.

- True
- False
- I don't know

Q15. Cervarix is available for males at the University of Florida Student Health Center.

- True
- False
- I don't know

Q16. Gardasil treats HPV.

- True
- False

- I don't know

Q17. Cervarix treats HPV.

- True
- False
- I don't know

Q18. HPV can be passed from person to person through vaginal sex.

- True
- False
- I don't know

Q19. HPV can be passed from person to person through anal sex.

- True
- False
- I don't know

Q20. HPV can be passed from person to person through oral sex.

- True
- False
- I don't know

Q21. HPV can be treated with antibiotics.

- True
- False
- I don't know

Q22. HPV is the same as herpes.

- True
- False
- I don't know

Q23. HPV is the same as HIV.

- True
- False
- I don't know

Q24. HPV can cause genital warts in men.

- True
- False
- I don't know

Q25. HPV can cause testicular cancer in men.

- True
- False
- I don't know

Q26. HPV can cause penile cancer in men.

- True
- False
- I don't know

Q27. HPV can cause anal cancer in men.

- True
- False
- I don't know

Q28. HPV can cause cervical cancer in men.

- True
- False
- I don't know

Q29. HPV can cause head and neck cancers in men.

- True
- False
- I don't know

Q30. Are you a Health Education major at the University of Florida?

- Yes
- No

Q31. Have you ever completed a Human Sexuality course/seminar/workshop?

- Yes

- ○ No

Q32. What is your sex?

- ○ Male
- ○ Female
- ○ Intersex/Transexual/Genderqueer

Q33. Have you ever served on active duty in the U.S. Armed Forces, military Reserves, or National Guard? *Active Duty does not include training for the Reserves or National Guard, but DOES include activation, for example, for the Persian Gulf War.*

- ○ Yes, now on active duty
- ○ Yes, on active duty during the last 12 months, but not now
- ○ Yes, on active duty in the past, but not during the last 12 months
- ○ No, training for Reserves or National Guard only
- ○ No, never served in the military

Q34. Are you a member of a social fraternity or sorority?

- ○ Yes
- ○ No
- ○ I am in the process of pledging/rushing/recruitment this semester

Q35. What is your race? (One or more categories may be selected)

- ☐ White
- ☐ Black or African American
- ☐ American Indian or Alaska Native
- ☐ Asian Indian
- ☐ Chinese
- ☐ Filipino
- ☐ Japanese
- ☐ Korean
- ☐ Vietnamese
- ☐ Other Asian
- ☐ Native Hawaiian
- ☐ Guamanian or Chamorro
- ☐ Samoan
- ☐ Other Pacific Islander

Q36. Are you Hispanic, Latino/a, or Spanish Origin? (One or more categories may be selected)

- ☐ No, not of Hispanic, Latino/a, or Spanish origin
- ☐ Yes, Mexican, Mexican American, Chicano/a
- ☐ Yes, Puerto Rican
- ☐ Yes, Cuban
- ☐ Yes, Another Hispanic, Latino/a, or Spanish origin

Q37. How would you classify your sexual orientation?

- ○ Asexual
- ○ Bisexual/Bi
- ○ Heterosexual/Straight
- ○ Homosexual/Gay/Lesbian/Queer
- ○ Unsure
- ○ Decline to answer

Q38. What is your current relationship status?

- ○ Married
- ○ In a committed relationship (with a steady partner)
- ○ Single (not dating)
- ○ Dating
- ○ Divorced
- ○ Widowed
- ○ Separated
- ○ ______ Other

Q39. What is your classification at the University of Florida?

- ○ Freshman
- ○ Sophomore
- ○ Junior
- ○ Senior
- ○ Graduate Student
- ○ Professional Student
- ○ Non-degree seeking student
- ○ I am not a student at the University of Florida

Q40. In which college is your current major?

- ○ College of Agricultural and Life Sciences
- ○ College of Business Administration
- ○ College of Dentistry
- ○ College of Design, Construction, and Planning

- College of Education
- College of Engineering
- College of Fine Arts
- College of Health and Human Performance
- College of Journalism and Communications
- College of Law
- College of Liberal Arts and Sciences
- College of Medicine
- College of Nursing
- College of Pharmacy
- College of Public Health and Health Professions
- College of Veterinary Medicine

Q41. Where do you currently live?

- On campus dormitory
- Off-campus dormitory
- Apartment
- House
- Other

Q42. Do you live with your parent(s) or guardian(s)?

- Yes
- No

Q43. What is your current health insurance status?

- I am covered under my parents' insurance.
- I have health insurance through my job not associated with the University of Florida.
- I have health insurance through my spouse.
- I have health insurance through the University of Florida.
- I am not insured.
- I don't know.

Q44. Have you received the Gardasil vaccine series?

- Yes, I have received all three vaccines
- Not yet; I have received two of the vaccines
- Not yet; I have received one vaccine
- No, I have not received any of the Gardasil vaccines
- I'm not sure

Q45. Do you intend to receive the Gardasil vaccine series?

- Yes, I have already received one vaccine
- Yes, I have already received two vaccines
- I have already received all three vaccines
- No, but I intend to begin the vaccine series in the next 30 days
- No, but I intend to begin the vaccine series in the next six months
- No, but I intend to begin the vaccine series at some in the future but not in the next six months
- No, I do not intend to begin the vaccine series
- I'm not sure

Q46. Have you ever had penile-vaginal intercourse?

- Yes
- No
- I'm not sure.
- Decline to answer

Q47. Have you ever performed oral sex?

- Yes
- No
- I'm not sure.
- Decline to answer

Q48. Have you ever received oral sex?

- Yes
- No
- I'm not sure.
- Decline to answer

Q49. Have you ever performed anal sex?

- Yes
- No
- I'm not sure.
- Decline to answer

Q50. Have you ever received anal sex?

- Yes
- No
- I'm not sure.

- Decline to answer

Q51. Within the last 12 months with how many partners have you oral sex?

- 0
- 1-2
- 3-5
- 6-9
- 10 or more
- Decline to answer

Q52. Within the last 12 months, with how many partners have you had penile-vaginal intercourse?

- 0
- 1-2
- 3-5
- 6-9
- 10 or more
- Decline to answer

Q53. Within the last 12 months, with how many partners have you had anal sex?

- 0
- 1-2
- 3-5
- 6-9
- 10 or more
- Decline to answer

Q54. Within the last 12 months, how often did you or your partner(s) use a male condom during oral sex?

- Always
- Most of the time
- Sometimes
- Rarely
- Never
- I did not have oral sex in the last 12 months
- I have never had oral sex
- Decline to answer

Q55. Within the last 12 months, how often did you or your partner(s) use a male condom during penile-vaginal intercourse?

- Always
- Most of the time
- Sometimes
- Rarely
- Never
- I did not have penile-vaginal intercourse in the last 12 months
- I have never had penile-vaginal intercourse
- Decline to answer

Q56. Within the last 12 months, how often did you or your partner(s) use a male condom during anal sex?

- Always
- Most of the time
- Sometimes
- Rarely
- Never
- I did not have anal sex in the last 12 months
- I have never had anal sex
- Decline to answer

Q57. Within the last 12 months, how often did you or your partner(s) use a female condom during oral sex?

- Always
- Most of the time
- Sometimes
- Rarely
- Never
- I did not have oral sex in the last 12 months
- I have never had oral sex
- Decline to answer

Q58. Within the last 12 months, how often did you or your partner(s) use a female condom during penile-vaginal intercourse?

- Always
- Most of the time
- Sometimes
- Rarely
- Never
- I did not have penile-vaginal intercourse in the last 12 months
- I have never had penile-vaginal intercourse
- Decline to answer

Q59. Within the last 12 months, how often did you or your partner(s) use a female condom during anal sex?

- ○ Always
- ○ Most of the time
- ○ Sometimes
- ○ Rarely
- ○ Never
- ○ I did not have anal sex in the last 12 months
- ○ I have never had anal sex
- ○ Decline to answer

Q60. Do you have any comments about this survey? Do you have any suggestions regarding how we can improve this survey?

Powered by Qualtrics
