# Supplementary material for: The Health Education Research Experience (HERE) program metadata dataset
Source: Data Brief. 2020 Jan 25;29:105180. doi: 10.1016/j.dib.2020.105180 (PMC7100622; doi:10.1016/j.dib.2020.105180)
Supplement: Multimedia component 7 [file mmc7.pdf]

Hookah Smoking Knowledge, Attitudes, and Behaviors of College Students

Informed Consent
Protocol Title: Hookah Smoking Knowledge, Attitudes, and Behaviors of College Students
Please read this consent document carefully before you decide to participate in this study.

Purpose of the research study:
This study addresses how university students perceive hookah smoking, heart disease, and lung diseases. The purpose of this study is to examine University of Florida students' attitudes and knowledge regarding hookah smoking, heart disease, and lung diseases. This research will supplement current health education literature. We are also interested in how you complete this survey (e.g. on your computer, your phone, or a tablet computer like an iPad). As such, the survey program, Qualtrics, will collect technical information addressed in the Confidentiality Section below.

Role of Research in HSC 3102:
One of the primary responsibilities of Certified Health Education Specialists is to *Conduct Evaluation and Research Related to Health Education*. As such, one of the goals of HSC 3102 – Personal and Family Health -- is to familiarize you with the research process in health education. To familiarize you with the research process in health education, we have created online surveys and introspective journal entries related to the content in each module.

Earning Health Education Research Experience Points:
This module includes a survey AND a journal entry. For this module, you may choose to participate in EITHER activity to receive your Health Education Research Experience points (5 points). Deadlines for this module's survey participation or journal entry are listed in the Sakai course website and correspond with the deadline for completing this module.

What you will be asked to do in the study:
You will be asked to take a 28-item questionnaire online through Qualtrics. In this study you will be asked about: 1) your previous and current cigarette smoking behaviors, 2) your perceived susceptibility to lung cancer, cardiovascular disease, and oral cancer, 3)your perceptions of the severity of lung cancer, cardiovascular disease, and oral cancer, and 4) your previous, current, and future hookah smoking behaviors. You will be asked to provide demographic information but will not be asked or required to provide personal identification information.

At the end of the survey, you will be directed to an external participation website that will collect your name and email address in order for the instructor to assign credit for participation in this study. If you choose to enter an email address in the external website form, you will receive a confirmation email for your records. If you choose to participate in the study and at the end of your participation you are not directed to the external website and/or do not receive a confirmation email, please contact [redacted] as soon as you encounter the technical difficulty.

In order to remove any risk that the researchers or any others could re-connect your name to your responses (for example, by using time-date information) from this survey and the external participation website, we will access the participation list immediately upon closure of this Module. The researchers will remove all potentially identifying

information, assign credit, and delete this information from our records and the Qualtrics server immediately after credit is entered into the grade book. The information collected within this survey instrument will be accessed and analyzed at a later time. With these security protections in place, it is very unlikely that a security breach would result in any adverse consequence for you, and anonymity will be maintained.

Time required:
Approximately 20-30 minutes

Risks and Benefits:
There are minimal risks associated with this study. We do not anticipate that you will benefit directly by participating in this research.

Compensation:
You will receive Health Education Research Experience participation credit for this module in HSC 3102. The participation credit for this module is five (5) points of your total course grade.

Confidentiality:
We will not connect your name or email address to your responses. Your information will be assigned a code number. The PI, Co-PI, and Supervisor will not track IP addresses or attach IP addresses to information. Your name will not be used in any report, presentation, or publication.

This survey contains a hidden item that collects information about your browser, browser version, operating system, screen resolution, flash version, java support version, and user agent from each device used to complete a survey. An example of the output created by Qualtrics for this item is below. (The output is the information that the researchers will be able to see when we analyze the results.)

Browser	Version	Operating System	Screen Resolution	Flash Version	Java Support	User Agent
Chrome	14.0.835.202	WOW64	1600x900	11.0.1	1	Mozilla/5.0 (Windows NT 6.1; WOW64) AppleWebKit/535.1 (KHTML, like Gecko) Chrome/14.0.835.202 Safari/535.1

This information identifies technical specifications of your device but cannot be used to identify you or your device.

Voluntary participation:
Your participation in this study is completely voluntary. There is no penalty for not participating. You can decline to answer any questions or quit taking the survey at any time without any penalty from your current or any future instructor. The survey software (Qualtrics) allows you to decline to answer any question to which you do not want to answer.

If you prefer to complete the journal entry for this module instead of this research, please close this window, return to the 3102 course website in Sakai and access the instructions for the module's journal entry located in the corresponding module page under the Course Materials tab.

Additional security information: This survey and the survey instrument used to collect information for assigning the HERE participation credit are both delivered through Qualtrics. Any information that could serve to reconnect the two surveys, for instance, information about when you took the surveys, and the order in which the surveys were taken by different individuals, will be deleted from both files prior to sending participation information to your instructor and prior to any analyses for research purposes.

There is a minimal risk that security of any online data may be breached, but Qualtrics provides password protection (only the PI and Co-PI can access the data), hosts data on secure servers, and all results are firewall protected so it is highly unlikely that a security breach of the online data would occur or would result in an adverse consequence for you. The Qualtrics privacy statement can be located by clicking on the following link: http://www.qualtrics.com/privacy-statement

Right to withdraw from the study:
You have the right to withdraw from the study at anytime without consequence. You will still receive the participation credit (5 points) if you withdraw from the study before the conclusion of the survey. If you choose to participate in the study and at the end of your participation you are not directed to the external website, please contact [redacted] as soon as you encounter the technical difficulty.
Whom to contact if you have questions about the study:
[redacted]

Whom to contact about your rights as a research participant in the study:
IRB02 Office, [redacted], University of Florida, Gainesville, FL 32611-2250; [redacted].

Agreement:
I have read the procedure described above. I voluntarily agree to participate in the study.

- ○ Begin survey (I consent to participating in this study)
- ○ I do not want to participate in this study
- ○ I have already participated in this study

Browser Meta Info

#EditSection, BrowserInfoExplanation#
Browser: **Chrome**
Version: **79.0.3945.88**
Operating System: **Windows NT 10.0**
Screen Resolution: **1280x1024**
Flash Version: **-1**
Java Support: **0**

User Agent: **Mozilla/5.0 (Windows NT 10.0; Win64; x64) AppleWebKit/537.36 (KHTML, like Gecko) Chrome/79.0.3945.88 Safari/537.36**

Percent of adults aged 18 years and over smoking cigarettes: NHIS 2008

Have you smoked at least 100 cigarettes in your entire life?

- Yes
- No

Do you now smoke cigarettes everyday, some days, or not at all?

- Everyday
- Some days
- Not at all

Hookah: Champion (1984)

To what extent do you agree or disagree with each of the following statements?

	Strongly Agree	Agree	Neither Agree nor Disagree	Disagree	Strongly Disagree
My chances of getting lung cancer are great.	○	○	○	○	○
My physical health makes it more likely that I will get lung cancer.	○	○	○	○	○
I feel that my chances of getting lung cancer in the future are good.	○	○	○	○	○
There is a good possibility that I will get lung cancer.	○	○	○	○	○
I worry a lot about getting lung cancer.	○	○	○	○	○
Within the next year I will get lung cancer.	○	○	○	○	○

To what extent do you agree or disagree with each of the following statements?

	Strongly Agree	Agree	Neither Agree nor Disagree	Disagree	Strongly Disagree
My chances of getting cardiovascular disease are great.	○	○	○	○	○
My physical health makes it more likely that I will get cardiovascular disease.	○	○	○	○	○
I feel that my chances of getting cardiovascular disease in the future are good.	○	○	○	○	○

	Strongly Agree	Agree	Neither Agree nor Disagree	Disagree	Strongly Disagree
There is a good possibility that I will get cardiovascular disease.	O	O	O	O	O
I worry a lot about getting cardiovascular disease.	O	O	O	O	O
Within the next year I will get cardiovascular disease.	O	O	O	O	O

To what extent do you agree or disagree with each of the following statements?

	Strongly Agree	Agree	Neither Agree nor Disagree	Disagree	Strongly Disagree
My chances of getting oral cancer are great.	O	O	O	O	O
My physical health makes it more likely that I will get oral cancer.	O	O	O	O	O
I feel that my chances of getting oral cancer in the future are good.	O	O	O	O	O
There is a good possibility that I will get oral cancer.	O	O	O	O	O
I worry a lot about getting oral cancer.	O	O	O	O	O
Within the next year I will get oral cancer.	O	O	O	O	O

Percieved Severity

To what extent do you agree or disagree with each of the following statements?

	Strongly Agree	Agree	Neither Agree nor Disagree	Disagree	Strongly Disagree
I believe that lung cancer is severe.	O	O	O	O	O
I believe that lung cancer is serious.	O	O	O	O	O
I believe that lung cancer is significant.	O	O	O	O	O

To what extent do you agree or disagree with each of the following statements?

	Strongly Agree	Agree	Neither Agree nor Disagree	Disagree	Strongly Disagree
I believe that cardiovascular disease is severe.	O	O	O	O	O
I believe that cardiovascular disease is serious.	O	O	O	O	O

	Strongly Agree	Agree	Neither Agree nor Disagree	Disagree	Strongly Disagree
I believe that cardiovascular disease is significant.	○	○	○	○	○

To what extent do you agree or disagree with each of the following statements?

	Strongly Agree	Agree	Neither Agree nor Disagree	Disagree	Strongly Disagree
I believe that oral cancer is severe.	○	○	○	○	○
I believe that oral cancer is serious.	○	○	○	○	○
I believe that oral cancer is significant.	○	○	○	○	○

Hookah/Waterpipe Use

The following questions will ask you about smoking hookah and your prior hookah smoking behavior. Hookah (also called a waterpipe) is used to pass charcoal heated air through a tobacco mixture and ultimately through a water-filled chamber. Here is a picture of a hookah/waterpipe.

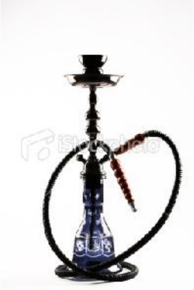

Have you ever smoked hookah (even one or two inhalations)?

○ Yes
○ No

Do you currently smoke hookah (at least once in the previous month)?

○ Yes
○ No

Did you smoke hookah at least once a month for three consecutive months in the past?

○ Yes
○ No

Do you think you can quit hookah smoking anytime you want?

○ Yes

○ No

Do you intend to quit hookah smoking?

○ Not at all
○ In the next month
○ In the next 6 months
○ In the future

Why have you smoked hookah?

Demographics

What is your sex?

○ Male
○ Female
○ Intersex/Transexual/Genderqueer

Are you a member of a social fraternity or sorority?

○ Yes
○ No
○ I am in the process of pledging/rushing/recruitment this semester

What is your race? (One or more categories may be selected)

☐ White
☐ Black or African American
☐ American Indian or Alaska Native
☐ Asian Indian
☐ Chinese
☐ Filipino
☐ Japanese
☐ Korean
☐ Vietnamese
☐ Other Asian
☐ Native Hawaiian
☐ Guamanian or Chamorro
☐ Samoan
☐ Other Pacific Islander

Are you Hispanic, Latino/a, or Spanish Origin? (One or more categories may be selected)

- [ ] No, not of Hispanic, Latino/a, or Spanish origin
- [ ] Yes, Mexican, Mexican American, Chicano/a
- [ ] Yes, Puerto Rican
- [ ] Yes, Cuban
- [ ] Yes, Another Hispanic, Latino/a, or Spanish origin

How would you classify your sexual orientation?

- ○ Asexual
- ○ Bisexual/Bi
- ○ Heterosexual/Straight
- ○ Homosexual/Gay/Lesbian/Queer
- ○ Unsure
- ○ Decline to answer

What is your current relationship status?

- ○ Married
- ○ In a committed relationship (with a steady partner)
- ○ Single (not dating)
- ○ Dating
- ○ Divorced
- ○ Widowed
- ○ Separated
- ○ ________ Other

What is your classification at the University of Florida?

- ○ Freshman
- ○ Sophomore
- ○ Junior
- ○ Senior
- ○ Graduate Student
- ○ Professional Student
- ○ Non-degree seeking student'
- ○ I am not a student at the University of Florida

In which college is your current major?

- ○ College of Agricultural and Life Sciences
- ○ College of Business Administration
- ○ College of Dentistry

- College of Design, Construction, and Planning
- College of Education
- College of Engineering
- College of Fine Arts
- College of Health and Human Performance
- College of Journalism and Communications
- College of Law
- College of Liberal Arts and Sciences
- College of Medicine
- College of Nursing
- College of Pharmacy
- College of Public Health and Health Professions
- College of Veterinary Medicine

How many courses in Health Education and Behavior have you completed at the University of Florida?

Are you a Health Education & Behavior major at the University of Florida?

- Yes
- No

Do you currently live with your parent(s) or guardian(s)?

- Yes
- No

Do you have any suggestions regarding how we can improve this survey?

Powered by Qualtrics
