# Supplementary material for: The Health Education Research Experience (HERE) program metadata dataset
Source: Data Brief. 2020 Jan 25;29:105180. doi: 10.1016/j.dib.2020.105180 (PMC7100622; doi:10.1016/j.dib.2020.105180)
Supplement: Multimedia component 8 [file mmc8.pdf]

Mental Health-Related Information Sources and College Students

Informed Consent
Protocol Title: Mental Health-Related Information Sources and College Students
Please read this consent document carefully before you decide to participate in this study.

Purpose of the research study:
This study addresses how university students perceive the credibility of mental health and mental illness Internet sources. The purpose of this study is to examine University of Florida students' perceptions and use Internet-based mental health resources. This research will supplement the current health education literature regarding college students at American universities. We are also interested in how you complete this survey (e.g. on your computer, your phone, or a tablet computer like an iPad). As such, the survey program, Qualtrics, will collect technical information addressed in the Confidentiality Section below.

Role of Research in HSC 3102:
One of the primary responsibilities of Certified Health Education Specialists is to *Conduct Evaluation and Research Related to Health Education*. As such, one of the goals of HSC 3102 – Personal and Family Health -- is to familiarize you with the research process in health education. To familiarize you with the research process in health education, we have created online surveys and introspective journal entries related to the content in each module.

Earning Health Education Research Experience Points:
This module includes a survey AND a journal entry. For this module, you may choose to participate in EITHER activity to receive your Health Education Research Experience points (5 points). Deadlines for the this module's survey participation or journal entry are listed in the Sakai course website and correspond with the deadline for completing this module.

What you will be asked to do in the study:
You will be asked to take a 34-item questionnaire online through Qualtrics. In this study you will be asked about your perceptions regarding Internet-based mental health resources. You will be asked to provide demographic information but will not be asked or required to provide personal identification information. The responses you provide are completely anonymous and cannot be connected with you at any time.

At the end of the survey, you will be directed to an external website which will collect your name and email address in order for the instructor to assign credit for participation in this study. If you choose to enter an email address in the external website form, you will receive a confirmation email for your records. If you choose to participate in the study and at the end of your participation you are not directed to the external website and/or do not receive a confirmation email, please contact [redacted] as soon as you encounter the technical difficulty.

Time required:
Approximately 20-30 minutes

Risks and Benefits:
There are minimal risks associated with this study. We do not anticipate that you will benefit directly by participating in this research.

Compensation:
You will receive Health Education Research Experience participation credit for this module in HSC 3102. The participation credit for this module is five (5) points of your total course grade.

Confidentiality:
We will not connect your name or email address to your responses. Your information will be assigned a code number. The PI, Co-PI, and Supervisor will not collect IP addresses, track IP addresses, or attach IP addresses to information. Your name will not be used in any report, presentation, or publication.

This survey contains a hidden item that collects information about your browser, browser version, operating system, screen resolution, flash version, java support version, and user agent from each device used to complete a survey. An example of the output created by Qualtrics for this item is below. (The output is the information that the researchers will be able to see when we analyze the results.)

Browser	Version	Operating System	Screen Resolution	Flash Version	Java Support	User Agent
Chrome	14.0.835.202	WOW64	1600x900	11.0.1	1	Mozilla/5.0 (Windows NT 6.1; WOW64) AppleWebKit/535.1 (KHTML, like Gecko) Chrome/14.0.835.202 Safari/535.1

This information identifies technical specifications of your device but cannot be used to identify you or your device.

Voluntary participation:
Your participation in this study is completely voluntary. There is no penalty for not participating. <u>You can decline to answer any questions or quit taking the survey at any time without any penalty from your current or any future instructor.</u> The survey software (Qualtrics) allows you to decline to answer any question to which you do not want to answer. The responses you provide are completely anonymous and cannot be connected with you at any time.

If you prefer to complete the journal entry for this module instead of this research, please close this window, return to the 3102 course website in Sakai and access the instructions for the module's journal entry located in the corresponding module page under the Course Materials tab.

Additional security:
The responses you provide are completely anonymous and cannot be connected with you at any time. The survey is delivered through Qualtrics. There is a minimal risk that security of any online data may be breached, but Qualtrics provides password protection (only the PI and Co-PI can access the data), hosts data on secure servers, and all results are firewall protected so it is highly unlikely that a security breach of the online data would occur or would result in an adverse consequence for you. The Qualtrics privacy statement can be located by clicking on the following link: http://www.qualtrics.com/privacy-statement

Right to withdraw from the study:
You have the right to withdraw from the study at anytime without consequence. You will still receive the participation credit (5 points) if you withdraw from the study before the conclusion of the survey. If you choose to participate in the study and at the end of your participation you are not directed to the external website, please contact [redacted] as soon as you encounter the technical difficulty.

Whom to contact if you have questions about the study:
[redacted]

Whom to contact about your rights as a research participant in the study:
IRB02 Office, [redacted] University of Florida, Gainesville, FL 32611-2250; [redacted]

Agreement:
I have read the procedure described above. I voluntarily agree to participate in the study.

- ○ Begin survey (I consent to participating in this study)
- ○ I do not want to participate in this study
- ○ I have already participated in this study

Emotional Support and Life Satisfaction (BRFSS: 2011)

Browser Meta Info

#EditSection, BrowserInfoExplanation#

Browser: **Chrome**
Version: **79.0.3945.88**
Operating System: **Windows NT 10.0**
Screen Resolution: **1280x1024**
Flash Version: **-1**
Java Support: **0**
User Agent: **Mozilla/5.0 (Windows NT 10.0; Win64; x64) AppleWebKit/537.36 (KHTML, like Gecko) Chrome/79.0.3945.88 Safari/537.36**

How often do you get the social and emotional support you need?

- ○ Always
- ○ Usually
- ○ Sometimes
- ○ Rarely
- ○ Never

In general, how satisfied are you with your life?

- ○ Very Satisfied
- ○ Satisfied
- ○ Dissatisfied
- ○ Very Dissatisfied

Social Media

On which social media sites do you have a profile or account?

	Yes	No
Blogger	○	○
Facebook	○	○
FlickR	○	○
Foursquare	○	○
Google Plus+	○	○
	Yes	No
MySpace	○	○
LinkedIn	○	○
LiveJournal	○	○
Second Life	○	○
Twitter	○	○
	Yes	No
Ning	○	○
YouTube	○	○
Other: Please List ______	○	○
I do not have any social media profiles	○	○

Which source do you FIRST consult when searching for MENTAL HEALTH information?

- Campus newspaper
- Facebook page
- Friend (who is not also a health educator, nurse, or physician)
- Google
- Health Educator (who is not also your parent, relative or a friend)
- Internet website (other than Google, Facebook, Twitter, and YouTube)
- Magazine
- Mobile App
- Nurse (who is not also your parent, relative, or friend)
- Parent(s)
- Physician (who is not also your parent, relative, or friend)
- Professor (who is not also your parent, relative, or friend)
- A relative other than your parent
- Television
- Twitter
- The University of Florida Counseling and Wellness Center
- YouTube
- It depends on what kind of information I am searching for.
- Other

Which source do you FIRST consult when searching for MENTAL ILLNESS information?

- Campus newspaper
- Facebook page
- Friend (who is not also a health educator, nurse, or physician)
- Google
- Health Educator (who is not also your parent, relative or a friend)
- Internet website (other than Google, Facebook, Twitter, and YouTube)
- Magazine
- Mobile App
- Nurse (who is not also your parent, relative, or friend)
- Parent(s)
- Physician (who is not also your parent, relative, or friend)
- Professor (who is not also your parent, relative, or friend)
- A relative other than your parent
- Television
- Twitter
- The University of Florida Counseling and Wellness Center

- YouTube
- It depends on what kind of information I am searching for.
- Other ______

Have you ever searched the Internet for information about any of the following topics?

	Yes	No
Adjustment to college life	○	○
Alcohol addiction	○	○
Anger management	○	○
Anticipation of graduation	○	○
Body image dissatisfaction	○	○
Changing majors	○	○
Child care issues	○	○
Conflict with parent(s)	○	○
	Yes	No
Death of a family member	○	○
Death of a friend	○	○
Depression	○	○
Eating disorders	○	○
End of semester stress	○	○
Engagement	○	○
Fight with boyfriend/girlfriend/ significant other	○	○
Financial difficulties	○	○
	Yes	No
Food addiction	○	○
Gender identity	○	○
Graduate school search	○	○
Illegal drug addiction	○	○
Increased course workload	○	○
Job search	○	○
Legal issues	○	○
Marriage	○	○
	Yes	No
Math anxiety	○	○
Parental divorce	○	○
Post-Traumatic Stress Disorder	○	○
Prescription drug addiction	○	○
Procrastination	○	○
Public speaking	○	○
Relaxation	○	○
Roommate conflict	○	○

	Yes	No
	Yes	No
Self-mutilation	○	○
Sexual assault	○	○
Sexual harassment	○	○
Sexual orientation	○	○
Suicide prevention	○	○
Test anxiety	○	○
Time management	○	○
Workplace conflict	○	○

To what extent do you think YouTube is a credible source for information on the following topics?

	Not at all credible	Slightly credible	Somewhat credible	Very credible	Completely credible
Addiction (Alcohol, Drug, Food)	○	○	○	○	○
Anxiety Disorders	○	○	○	○	○
Attention Deficit Hyperactivity Disorder (ADHD, ADD)	○	○	○	○	○
Body Image	○	○	○	○	○
Death of a friend or family member	○	○	○	○	○
Depression	○	○	○	○	○
Eating Disorders	○	○	○	○	○
Sexual Assault	○	○	○	○	○
Suicide Prevention	○	○	○	○	○

To what extent do you think FACEBOOK is a credible source for information on the following topics?

	Not at all credible	Slightly credible	Somewhat credible	Very credible	Completely credible
Addiction (Alcohol, Drug, Food)	○	○	○	○	○
Anxiety Disorders	○	○	○	○	○
Attention Deficit Hyperactivity Disorder (ADHD, ADD)	○	○	○	○	○
Body Image	○	○	○	○	○
Death of a friend or family member	○	○	○	○	○
Depression	○	○	○	○	○
Eating Disorders	○	○	○	○	○
Sexual Assault	○	○	○	○	○
Suicide Prevention	○	○	○	○	○

To what extent do you think AN APP is a credible source for information on the following topics?

	Not at all credible	Slightly credible	Somewhat credible	Very credible	Completely credible

	Not at all credible	Slightly credible	Somewhat credible	Very credible	Completely credible
Addiction (Alcohol, Drug, Food)	○	○	○	○	○
Anxiety Disorders	○	○	○	○	○
Attention Deficit Hyperactivity Disorder (ADHD, ADD)	○	○	○	○	○
Body Image	○	○	○	○	○
Death of a friend or family member	○	○	○	○	○
Depression	○	○	○	○	○
Eating Disorders	○	○	○	○	○
Sexual Assault	○	○	○	○	○
Suicide Prevention	○	○	○	○	○

To what extent do you think TWITTER is a credible source for information on the following topics?

	Not at all credible	Slightly credible	Somewhat credible	Very credible	Completely credible
Addiction (Alcohol, Drug, Food)	○	○	○	○	○
Anxiety Disorders	○	○	○	○	○
Attention Deficit Hyperactivity Disorder (ADHD, ADD)	○	○	○	○	○
Body Image	○	○	○	○	○
Death of a friend or family member	○	○	○	○	○
Depression	○	○	○	○	○
Eating Disorders	○	○	○	○	○
Sexual Assault	○	○	○	○	○
Suicide Prevention	○	○	○	○	○

Within the last 30 days, have you searched for information on the Internet related to your own mental health or mental illness?

- ○ Yes
- ○ No
- ○ I'm not sure

What topic did you search?

____

Within the last 30 days, have you searched for information on the Internet related to someone else's mental health or mental illness?

- ○ Yes
- ○ No
- ○ I'm not sure

What topic did you search?

Within the last 30 days, have you used your cell phone to search the Internet for mental health or mental illness information?

- Yes
- No
- I'm not sure

What topic did you search?

Demographics(+Module 24 items regarding Veterans' Health)

What is your age?

What is your sex?

- Male
- Female

Are you a member of a social fraternity or sorority?

- Yes
- No
- I am in the process of pledging/rushing/recruitment this semester

Have you ever served on active duty in the U.S. Armed Forces, military Reserves, or National Guard? *Active Duty does not include training for the Reserves or National Guard, but DOES include activation, for example, for the Persian Gulf War.*

- Yes, now on active duty
- Yes, on active duty during the last 12 months, but not now
- Yes, on active duty in the past, but not during the last 12 months
- No, training for Reserves or National Guard only
- No, never served in the military

Did you ever serve in a combat or war zone?

- Yes
- No

What is your race? (One or more categories may be selected)

- [ ] White
- [ ] Black or African American
- [ ] American Indian or Alaska Native
- [ ] Asian Indian
- [ ] Chinese
- [ ] Filipino
- [ ] Japanese
- [ ] Korean
- [ ] Vietnamese
- [ ] Other Asian
- [ ] Native Hawaiian
- [ ] Guamanian or Chamorro
- [ ] Samoan
- [ ] Other Pacific Islander

Are you Hispanic, Latino/a, or Spanish Origin? (One or more categories may be selected)

- [ ] No, not of Hispanic, Latino/a, or Spanish origin
- [ ] Yes, Mexican, Mexican American, Chicano/a
- [ ] Yes, Puerto Rican
- [ ] Yes, Cuban
- [ ] Yes, Another Hispanic, Latino/a, or Spanish origin

Has a doctor or other health professional ever told you that you have depression, anxiety, or post traumatic stress disorder (PTSD)?

- ○ Yes
- ○ No

How many children less than 18 years of age live in your household? (To answer this question, please type a number in the box below.)

[ ]

What is your classification at the University of Florida?

- ○ Freshman
- ○ Sophomore
- ○ Junior
- ○

Senior

- Graduate Student
- Professional Student
- Non-degree seeking student
- I am not a student at the University of Florida

In which college is your current major?

- College of Agricultural and Life Sciences
- College of Business Administration
- College of Dentistry
- College of Design, Construction, and Planning
- College of Education
- College of Engineering
- College of Fine Arts
- College of Health and Human Performance
- College of Journalism and Communications
- College of Law
- College of Liberal Arts and Sciences
- College of Medicine
- College of Nursing
- College of Pharmacy
- College of Public Health and Health Professions
- College of Veterinary Medicine

How would you classify your sexual orientation?

- Asexual
- Bisexual/Bi
- Heterosexual/Straight
- Homosexual/Gay/Lesbian/Queer
- Unsure
- Decline to answer

What is your current relationship status?

- Married
- In a committed relationship (with a steady partner)
- Single (not dating)
- Dating
- Divorced

- Widowed
- Separated
- Other

What is your current health insurance status?

- I am covered under my parents' insurance.
- I have health insurance through my job not associated with the University of Florida.
- I have health insurance through my spouse.
- I have health insurance through the University of Florida.
- I am not insured.
- I don't know.

Where do you currently live?

- On campus dormitory
- Off-campus dormitory
- Apartment
- House
- Other

What is your approximate grade point average (one decimal place)?

Do you have any comments regarding this survey or how we can improve this survey for future participants?
