# Supplementary material for: The Health Education Research Experience (HERE) program metadata dataset
Source: Data Brief. 2020 Jan 25;29:105180. doi: 10.1016/j.dib.2020.105180 (PMC7100622; doi:10.1016/j.dib.2020.105180)
Supplement: Multimedia component 9 [file mmc9.pdf]

University Student Perceptions and Behaviors Regarding Nutrition Facts Labels

Informed Consent
Protocol Title: University Student Perceptions and Behaviors Regarding Nutrition Facts Labels
Please read this consent document carefully before you decide to participate in this study.

Purpose of the research study:
The purpose of this study is to examine University of Florida students' knowledge of the Food and Drug Administration Nutrition Facts Label and gain a better understanding of students' label reading behaviors. This research will supplement the current literature on the health and nutritional literacy of college students at American universities. We are also interested in how you complete this survey (e.g. on your computer, your phone, or a tablet computer like an iPad). As such, the survey program, Qualtrics, will collect technical information addressed in the Confidentiality Section below.

Role of Research in HSC 3102:
One of the primary responsibilities of Certified Health Education Specialists is to *Conduct Evaluation and Research Related to Health Education*. As such, one of the goals of HSC 3102 – Personal and Family Health -- is to familiarize you with the research process in health education. To familiarize you with the research process in health education, we have created online surveys and introspective journal entries related to the content in each module.

Earning Health Education Research Experience Points:
This module includes a survey AND a journal entry. For this module, you may choose to participate in EITHER activity to receive your Health Education Research Experience points (5 points). Deadlines for the this module's survey participation or journal entry are listed in the Sakai course website and correspond with the deadline for completing this module.

What you will be asked to do in the study:
You will be asked to take a 59-item questionnaire online through Qualtrics. In this study you will be asked your opinions about the accuracy and readability of Nutrition Facts food labels. You will be presented with an image of a sample Nutrition Facts Label and be asked to click on the area that you first look for information. You will also be asked to answer questions based on sample Nutrition Facts Labels. You will be asked to provide demographic information but will not be asked or required to provide personal identification information. The responses you provide are completely anonymous and cannot be connected with you at any time.

At the end of the survey, you will be directed to an external website which will collect your name in order for the instructor to assign credit for participation in this study. If you choose to participate in the study and at the end of your participation you are not directed to the external website, please contact [redacted] as soon as you encounter the technical difficulty.

Time required:
Approximately 20-30 minutes

Risks and Benefits:
There are minimal risks associated with this study. We do not anticipate that you will benefit directly by participating in this research.

Compensation:
You will receive Health Education Research Experience participation credit for this module in HSC 3102. The participation credit for this module is five (5) points of your total course grade.

Confidentiality:
We will not connect your name or email address to your responses. Your information will be assigned a code number. The PI, Co-PI, and Supervisor will not collect IP addresses, track IP addresses, or attach IP addresses to information. Your name will not be used in any report, presentation, or publication.

This survey contains a hidden item that collects information about your browser, browser version, operating system, screen resolution, flash version, java support version, and user agent from each device used to complete a survey. An example of the output created by Qualtrics for this item is below. (The output is the information that the researchers will be able to see when we analyze the results.)

Browser	Version	Operating System	Screen Resolution	Flash Version	Java Support	User Agent
Chrome	14.0.835.202	WOW64	1600x900	11.0.1	1	Mozilla/5.0 (Windows NT 6.1; WOW64) AppleWebKit/535.1 (KHTML, like Gecko) Chrome/14.0.835.202 Safari/535.1

This information identifies technical specifications of your device but cannot be used to identify you or your device.

Voluntary participation:
Your participation in this study is completely voluntary. There is no penalty for not participating. You can decline to answer any questions or quit taking the survey at any time without any penalty from your current or any future instructor. The survey software (Qualtrics) allows you to decline to answer any question to which you do not want to answer. The responses you provide are completely anonymous and cannot be connected with you at any time.

If you prefer to complete the journal entry for this module instead of this research, please close this window, return to the 3102 course website in Sakai and access the instructions for the module's journal entry located in the corresponding module page under the Course Materials tab.

Additional security:
The responses you provide are completely anonymous and cannot be connected with you at any time. The survey is delivered through Qualtrics. There is a minimal risk that security of any online data may be breached, but Qualtrics provides password protection (only the PI and Co-PI can access the data), hosts data on secure servers, and all results are firewall protected so it is highly unlikely that a security breach of the online data would occur or would result in an adverse consequence for you. The Qualtrics privacy statement can be located by clicking on the following link: http://www.qualtrics.com/privacy-statement

Right to withdraw from the study:
You have the right to withdraw from the study at anytime without consequence. You will still receive the participation credit (5 points) if you withdraw from the study before the conclusion of the survey. If you choose to participate in the study and at the end of your participation you are not directed to the external website, please contact [redacted] as soon as you encounter the technical difficulty.

Whom to contact if you have questions about the study:
[redacted]

Whom to contact about your rights as a research participant in the study:
IRB02 Office, [redacted], University of Florida, Gainesville, FL 32611-2250; [redacted]

Agreement:
I have read the procedure described above. I voluntarily agree to participate in the study.

- ○ Begin survey (I consent to participating in this study)
- ○ I do not want to participate in this study
- ○ I have already participated in this study

Original Question Block

Where is the first place you look on a Nutrition Facts Label? Click the label where you first look.

Nutrition Facts

Serving Size 1 cup (228g)
Servings Per Container 2

Amount Per Serving

Calories 250	Calories from Fat 110
	% Daily Value*
Total Fat 12g	18%
Saturated Fat 3g	15%
Trans Fat 1.5g	
Cholesterol 30mg	10%
Sodium 470mg	20%
Total Carbohydrate 31g	10%
Dietary Fiber 0g	0%
Sugars 5g	
Protein 5g	
Vitamin A	4%
Vitamin C	2%
Calcium	20%
Iron	4%

* Percent Daily Values are based on a 2,000 calorie diet. Your Daily Values may be higher or lower depending on your calorie needs:

	Calories:	2,000	2,500
Total Fat	Less than	65g	80g
Sat Fat	Less than	20g	25g
Cholesterol	Less than	300mg	300mg
Sodium	Less than	2,400mg	2,400mg
Total Carbohydrate		300g	375g
Dietary Fiber		25g	30g

Browser Meta Info

#EditSection, BrowserInfoExplanation#
Browser: **Chrome**
Version: **79.0.3945.88**
Operating System: **Windows NT 10.0**
Screen Resolution: **1280x1024**
Flash Version: **-1**
Java Support: **0**
User Agent: **Mozilla/5.0 (Windows NT 10.0; Win64; x64) AppleWebKit/537.36 (KHTML, like Gecko) Chrome/79.0.3945.88 Safari/537.36**

Nutrient information provided in the "Nutrition Facts" label is accurate.

- Strongly Disagree
- Disagree
- No Opinion
- Agree
- Strongly Agree

Nutritional claims such as "low fiber" and "no fat," that appear on the front of the food packages are truthful.

- Strongly Disagree

- Disagree
- No Opinion
- Agree
- Strongly Agree

The "Nutrition Facts" label is easy to understand.

- Strongly Disagree
- Disagree
- No Opinion
- Agree
- Strongly Agree

Which of the following do you most often look for on a food label?

- Fat
- Fiber
- Nutrient Value
- Cholesterol
- Sugars
- Sodium
- Calories

Food Label Items: NHANES Flexible Consumer Behavior Survey Module

Many food packages contain an expiration date such as "use by" or "sell by". How often do you use the expiration date when deciding to buy a food product?

- Always
- Most of the time
- Sometimes
- Rarely
- Never

Some food packages contain health claims about the benefits of nutrients or foods (For example, "Diets low in sodium may reduce the risk of high blood pressure", or "Diets rich in calcium may reduce your risk of osteoporosis"). How often do you use this kind of health claim when deciding to buy a food product?

- Always
- Most of the time
- Sometimes
- Rarely
- Never

For the next five questions, please refer to this picture.

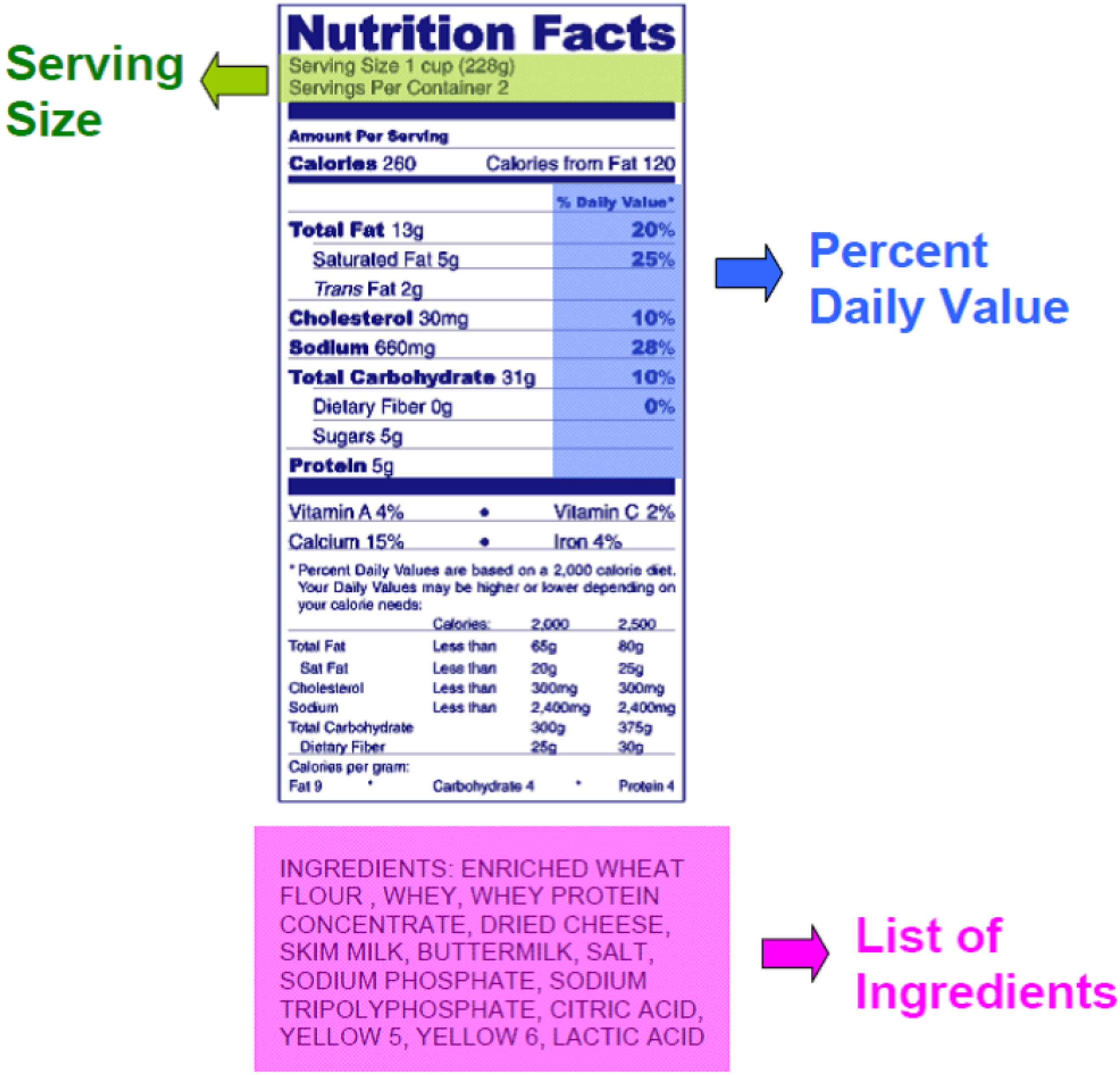

The "Nutrition Facts panel" of a food label is everything in this picture except the list of ingredients in pink. How often do you use the Nutrition Facts panel when deciding to buy a food product?

- ○ Always
- ○ Most of the time
- ○ Sometimes
- ○ Rarely
- ○ Never

How about the *list of ingredients*? How often do you use the *list of ingredients* on a food label {such as the part colored in pink in the picture above} when deciding to buy a food product?

- ○ Always

- Most of the time
- Sometimes
- Rarely
- Never

How about the information on the *serving size*? How often do you use the information on the *serving size* on a food label {such as the part colored in green in the picture above} when deciding to buy a food product?

- Always
- Most of the time
- Sometimes
- Rarely
- Never

How about the information on the *percent daily value*? How often do you use information on the *percent daily value* {such as the part colored in blue in the picture above} when deciding to buy a food product?

- Always
- Most of the time
- Sometimes
- Rarely
- Never

In thinking about the *serving size* on this food label, what does serving size mean to you? You may give more than one answer.

- [ ] The amount of this food that people should eat
- [ ] The amount of this food that people usually eat
- [ ] Something that makes it easier to compare foods
- [ ] Other

How often do you look for nutrition information on the food label when you buy *snack items* like chips, popcorn, or pretzels?

- Always
- Most of the time
- Sometimes
- Rarely
- Never

How often do you look for nutrition information on the food label when you buy *breakfast cereals?*

- Always
- Most of the time

- Sometimes
- Rarely
- Never

How often do you look for nutrition information on the food label when you buy *salad dressings?*

- Always
- Most of the time
- Sometimes
- Rarely
- Never

How often do you look for nutrition information on the food label when you buy *raw meat, poultry, or fish?*

- Always
- Most of the time
- Sometimes
- Rarely
- Never

How often do you look for nutrition information on the food label when you buy *processed meat* products like hot dogs or bologna?

- Always
- Most of the time
- Sometimes
- Rarely
- Never

How often do you look for nutrition information on the food label when you buy *bread?*

- Always
- Most of the time
- Sometimes
- Rarely
- Never

What is the reason or reasons that you check the food label when deciding to buy a food product? You may give more than one answer.

- [ ] To watch my weight and/or lose weight
- [ ] A family member is trying to watch their weight and/or lose weight
- [ ] To watch for diabetes, high triglycerides, high cholesterol, high blood pressure or other health conditions
- [ ] A family member has a health condition (for example, diabetes, high triglycerides, high cholesterol, high blood pressure, etc)

- I am allergic to certain food(s)
- To avoid certain ingredients (such as MSG, high fructose corn syrup, color dyes, artificial preservatives, or hydrogenated oils, etc
- To increase certain nutrients in my diet (such as fiber, calcium, etc)
- To compare which brand/food is better/healthier
- To make better/healthier choices for me and my family
- Other

What is the reason or reasons that you rarely or never check the food label when deciding to buy a food product? You may give more than one answer.

- I don't have the time
- The print is too small for me to read
- I'm satisfied with my health so there is no need for me to check
- I have a good diet so there is no need to check labels
- I usually buy foods that I'm used to, so I don't feel that I need to check labels
- I buy what I like, I don't care about the labels
- I don't think the food labels are important to me
- I won't know what to look for even if I read the labels
- I can't read English that well
- Other

Food Security

Does your current household include a child or children under the age of 17?

- Yes
- No

"I worried whether my food would run out before I got money to buy more."

Was that often true, sometimes true, or never true for you in the last 12 months?

- Often true
- Sometimes true
- Never true

"The food that I bought just didn't last and I didn't have money to get more."

Was that often true, sometimes true, or never true for you in the last 12 months?

- Often true
- Sometimes true
- Never true

"I couldn't afford to eat balanced meals."

Was that often true, sometimes true, or never true for you in the last 12 months?

- Often true
- Sometimes true
- Never true

In the last 12 months, did you ever cut the size of your meals or skip meals because there wasn't enough money for food?

- Yes
- No

How often did this happen?

- Almost every month
- Some months but not every month
- In only 1 or 2 months

In the last 12 months, did you ever eat less than you felt you should because there wasn't enough money for food?

- Yes
- No

In the last 12 months, were you ever hungry, but didn't eat, because there wasn't enough money for food?

- Yes
- No

In the last 12 months, did you lose weight because there wasn't enough money for food?

- Yes
- No

In the last 12 months did you or other adults in your household ever not eat for a whole day because there wasn't enough money for food?

- Yes
- No

How often did this happen?

- Almost every month
- Some months but not every month
- Only 1 or 2 months

Demographic Information

What is your sex?

- Male
- Female

What is your enrollment status?

- Full-time
- Part-time
- Other

Are you an international student?

- Yes
- No

Are you concerned about how you will finance your educational expenses (i.e. Tuition, books, supplies, lab fees, and similar items) this semester?

- Yes
- No

What type of financial resource are you PRIMARILY using to finance your educational expenses (i.e. Tuition, books, supplies, lab fees, and similar items) this semester?

- Family resources (financial assistance from parents, guardians, or other relatives)
- Grant(s)
- Loan(s)
- Fellowship(s)
- Credit card(s)
- Personal savings
- Work-study program
- University employment
- Employment outside of the University of Florida
- Scholarship(s)
- Military funding
- Other ____________

Have you ever read, heard, or been taught information about how to use food labels?

- Yes
- No

What is your class rank?

- Freshman
- Sophomore
- Junior
- Senior
- Graduate student
- Non-degree seeking student

What month were you born?

What year were you born?

Please indicate your height in inches.

Please indicate your weight in pounds. (rounded to nearest whole number)

Are you currently on a university meal plan?

- Yes
- No

Which meal plan?

- 5 day access (resident)
- 7 day access (resident)
- 14 meal plan (resident)
- 40 meal plan (commuter)
- 60 meal plan (commuter)

What is your MOTHER'S highest level of education?

- Less than High School
- High School Degree/GED
- Some College
- Associates Degree (2 yr college degree)

- Bachelor Degree (4 yr college degree)
- Masters Degree
- Doctoral Degree
- Professional Degree (M.D., J.D.)

What is your FATHER'S highest level of education?

- Less than High School
- High School Degree/GED
- Some College
- Associates Degree (2 yr college degree)
- Bachelor Degree (4 yr college degree)
- Masters Degree
- Doctoral Degree
- Professional Degree (M.D., J.D.)

Have you smoked cigarettes in the last 30 days?

- Yes
- No

For this question, one drink of alcohol is defined as a 12 oz. can or bottle of beer or wine cooler, a 5 oz. glass of wine, or a 1.5 oz. shot of liquor straight or in a mixed drink.

In the last 30 days, have you had 5 or more drinks (males) or 4 or more drinks (females) of alcohol in about 2 hours?

- Yes
- No

The Surgeon General recommends at least 30 minutes of physical activity 5 times per week. Do you meet this recommendation?

- Yes
- No

Do you take nutritional supplements?

- Yes
- No

Are you a Health Education major at the University of Florida?

- Yes
- No

What is your race?

- White/Caucasian
- African American
- Asian
- Native American
- Pacific Islander
- Other

How would you classify your sexual orientation?

- Asexual
- Bisexual/Bi
- Heterosexual/Straight
- Homosexual/Gay/Lesbian/Queer
- Unsure
- Decline to answer

What is your current relationship status?

- Married
- In a committed relationship (with a steady partner)
- Single (not dating)
- Dating
- Divorced
- Widowed
- Separated
- Other

Where do you currently live?

- On campus dormitory
- Off-campus dormitory
- Apartment
- House
- Other

Do you live with your parent(s) or guardian(s)?

- Yes
- No

What is your current health insurance status?

- I am covered under my parents' insurance.
- I have health insurance through my job not associated with the University of Florida.
- I have health insurance through my spouse.
- I have health insurance through the University of Florida.
- I am not insured.
- I don't know.
