# Supplementary material for: The Health Education Research Experience (HERE) program metadata dataset
Source: Data Brief. 2020 Jan 25;29:105180. doi: 10.1016/j.dib.2020.105180 (PMC7100622; doi:10.1016/j.dib.2020.105180)
Supplement: Multimedia component 10 [file mmc10.pdf]

[redacted]

**Informed Consent**
Protocol Title: Obesity Campaigns
Please read this consent document carefully before you decide to participate in this study.

**Purpose of the research study:**
The purpose of this study is to identify reactions to obesity-related images among a sample of college sutdents. More specifically, this study will look at how different images and messages motivate and increase self-efficacy to change behaviors related to obesity. The results of this study will add to the bodies of obesity-related media campaign literature and address how to best deliver obesity-related images to students at a university level. We are also interested in how you complete this survey (e.g. on your computer, your phone, or a tablet computer like an iPad). As such, the survey program, Qualtrics, will collect technical information addressed in the Confidentiality Section below.

**Role of Research in HSC 3102:**
One of the primary responsibilities of Certified Health Education Specialists is to *Conduct Evaluation and Research Related to Health Education*. As such, one of the goals of HSC 3102 – Personal and Family Health -- is to familiarize you with the research process in health education. To familiarize you with the research process in health education, we have created online surveys and introspective journal entries related to the content in each module.

**Earning Health Education Research Experience Points:**
This module includes a survey AND a journal entry. For this module, you may choose to participate in EITHER activity to receive your Health Education Research Experience points (5 points). Deadlines for this module's survey participation or journal entry are listed in the Sakai course website and correspond with the deadline for completing this module.

**What you will be asked to do in this study:**
You will be asked to take a 109-item questionnaire online through Qualtrics. In this study you will be asked about your attitudes and perceptions toward print advertisements addressing obesity. You will be asked to provide demographic information but will not be asked or required to to provide personal identification information.

At the end of the survey, you will be directed to an external participation website which will collect your name and email address in order for the instructor to assign credit for participation in this study. If you choose to enter an email address in the external website form, you will receive a confirmation email for your records. If you choose to participate in the study and at the end of your participation you are not directed to the external website and/or do not receive a confirmation email, please contact [redacted] as soon as you encounter the technical difficulty.

In order to remove any risk that the researchers or any others could re-connect your name to your responses (for example, by using time-date information) from this survey and the external participation website, we will access the participation list immediately upon closure of this Module. The researchers will remove all potentially identifying information from the files related to this study, assign credit, and delete this information from our records and the Qualtrics server immediately after credit is entered into the grade book. The information collected within this survey instrument will be accessed and analyzed at a later time. With these security protections in place, it is very unlikely that a security breach would result in any adverse consequence for you, and anonymity will be maintained.

**Time required:**
Approximately 20-30 minutes

**Risks and Benefits:**
There are minimal risks associated with this study. We do not anticipate that you will benefit directly by participating in this research.

**Compensation:**
You will receive Health Education Research Experience participation credit for this module in HSC 3102. The participation credit for this module is five (5) points of your total course grade.

**Confidentiality:**
We will not connect your name or email address to your responses. Your information will be assigned a code number. The PI, Co-PI, and Supervisor will not track IP addresses or attach IP addresses to information. Your name will not be used in any report, presentation, or publication.

This survey contains a hidden item that collects information about your browser, browser version, operating system, screen resolution, flash version, java support version, and user agent from each device used to complete a survey. An example of the output created by Qualtrics for this item is below. (The output is the information that the researchers will be able to see when we analyze the results.)

| Browser | Version | Operating System | Screen Resolution | Flash Version | Java Support | User Agent |
|---|---|---|---|---|---|---|
| Chrome | 14.0.835.202 | WOW64 | 1600x900 | 11.0.1 | 1 | Mozilla/5.0 (Windows NT 6.1; WOW64) AppleWebKit/535.1 (KHTML, like Gecko) Chrome/14.0.835.202 Safari/535.1 |

This information identifies technical specifications of your device but cannot be used to identify you or your device.

**Voluntary participation:**
Your participation in this study is completely voluntary. There is no penalty for not participating. You can decline to answer any questions or quit taking the survey at any time without any penalty from your current or any future instructor. The survey software (Qualtrics) allows you to decline to answer any question to which you do not want to answer. The responses you provide are completely anonymous and cannot be connected with you at any time.

If you prefer to complete the journal entry for this module instead of this research, please close this window, return to the 3102 course website in Sakai and access the instructions for the module's journal entry located in the corresponding module page under the Course Materials tab.

**Additional security information:** This survey and the survey instrument used to collect information for assigning the HERE participation credit are both delivered through Qualtrics. Any information that could serve to reconnect the two surveys, for instance information about when you took the surveys, and the order in which the surveys were taken by different individuals, will be deleted from both files prior to sending participation information to your instructor and prior to any analyses for research purposes.

There is a minimal risk that security of any online data may be breached, but Qualtrics provides password protection (only the PI and Co-PI can access the data), hosts data on secure servers, and all results are firewall protected so it is highly unlikely that a security breach of the online data would occur or would result in an adverse consequence for you. The Qualtrics privacy statement can be located by clicking on the following link: http://www.qualtrics.com/privacy-statement

**Right to withdraw from the study:**
You have the right to withdraw from the study at anytime without consequence. You will still receive the participation credit (5 points) if you withdraw from the study before the conclusion of the survey. If you choose to participate in the study and at the end of your participation you are not directed to the external website, please contact [redacted] [redacted] as soon as you encounter the technical difficulty.

**Whom to contact if you have questions about the study:**
[redacted]

**Whom to contact about your rights as a research participant in the study:**
IRB02 Office, [redacted], University of Florida, Gainesville, FL 32611-2250; [redacted].

**Agreement:**
**I have read the procedure described above. I voluntarily agree to participate in the study.**

- Begin survey (I consent to participating in this study)
- I do not want to participate in this study
- I have already participated in this study

**Browser Meta Info**

*#EditSection, BrowserInfoExplanation#*
Browser: **Chrome**
Version: **79.0.3945.88**
Operating System: **Windows NT 10.0**
Screen Resolution: **1280x1024**
Flash Version: **-1**
Java Support: **0**
User Agent: **Mozilla/5.0 (Windows NT 10.0; Win64; x64) AppleWebKit/537.36 (KHTML, like Gecko) Chrome/79.0.3945.88 Safari/537.36**

**Advertisements**

**Use the image below to answer the following questions**

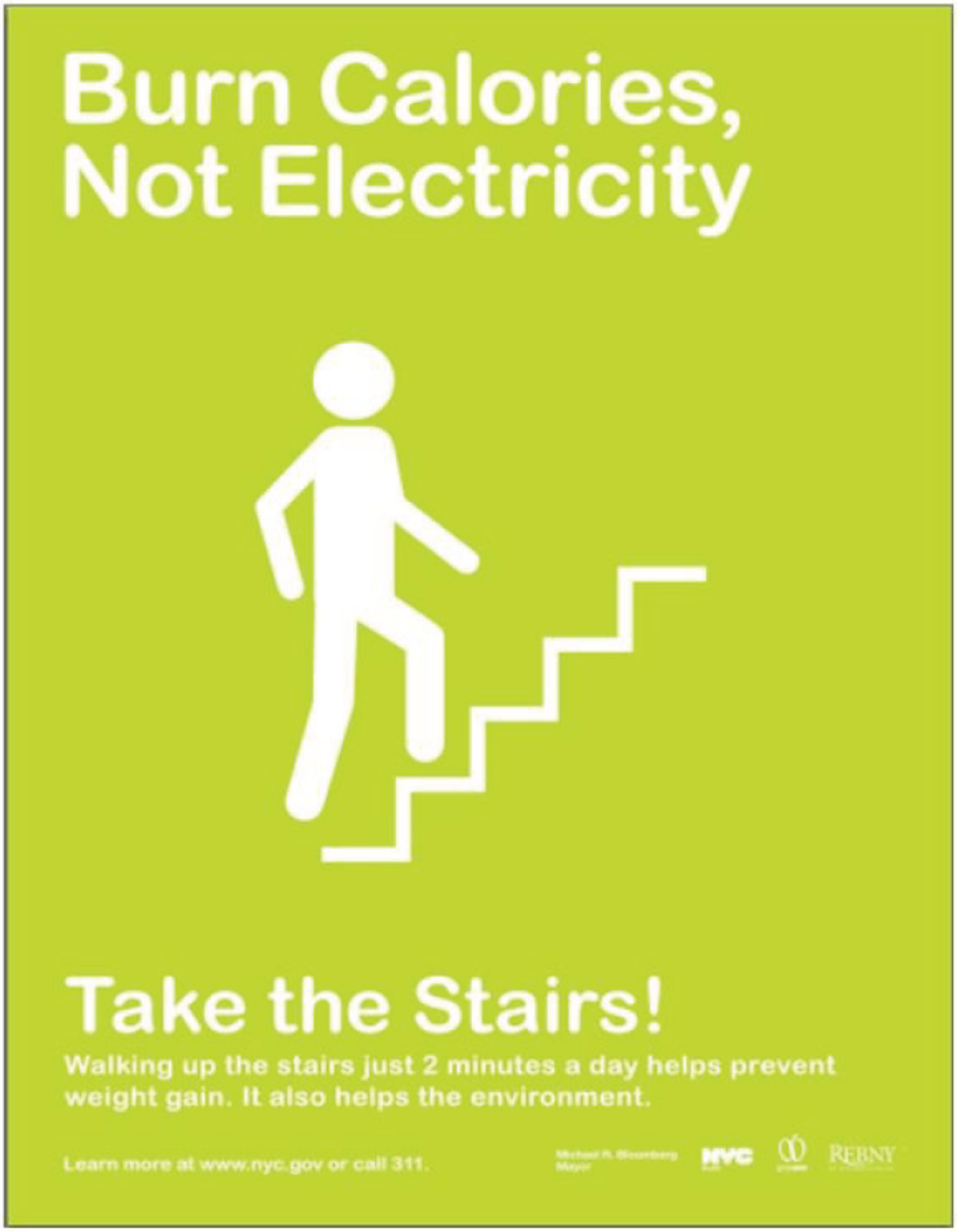


**How would you describe the message above?**

| | Strongly Disagree | Disagree | Neither Agree nor Disagree | Agree | Strongly Agree |
|---|---|---|---|---|---|
| Helpful | ○ | ○ | ○ | ○ | ○ |
| Informative | ○ | ○ | ○ | ○ | ○ |
| Important | ○ | ○ | ○ | ○ | ○ |
| Motivating | ○ | ○ | ○ | ○ | ○ |
| Credible Information | ○ | ○ | ○ | ○ | ○ |
| Appropriate | ○ | ○ | ○ | ○ | ○ |
| Stigmatizing | ○ | ○ | ○ | ○ | ○ |
| Useful | ○ | ○ | ○ | ○ | ○ |

**How would you describe the photo above?**

| | Strongly Disagree | Disagree | Neither Agree nor Disagree | Agree | Strongly Agree |
|---|---|---|---|---|---|

| | Strongly Disagree | Disagree | Neither Agree nor Disagree | Agree | Strongly Agree |
|---|---|---|---|---|---|
| Helpful | ○ | ○ | ○ | ○ | ○ |
| Informative | ○ | ○ | ○ | ○ | ○ |
| Important | ○ | ○ | ○ | ○ | ○ |
| Motivating | ○ | ○ | ○ | ○ | ○ |
| Credible Information | ○ | ○ | ○ | ○ | ○ |
| Appropriate | ○ | ○ | ○ | ○ | ○ |
| Stigmatizing | ○ | ○ | ○ | ○ | ○ |
| Useful | ○ | ○ | ○ | ○ | ○ |

**Are you more likely to take the stairs after seeing this image?**

- ○ Definitely will not
- ○ Probably will not
- ○ Don't know
- ○ Probably will
- ○ Definitely will

**Please indicate how much you agree with the following statement: I intend to take the stairs more often after seeing this image.**

- ○ Strongly Disagree
- ○ Disagree
- ○ Neither Agree nor Disagree
- ○ Agree
- ○ Strongly Agree

**If you would try to take the stairs more often, would you be able to do this?**

- ○ Definitely Not
- ○ Probably not
- ○ Maybe
- ○ Probably yes
- ○ Definitely yes

**Use the image below to answer the following questions**

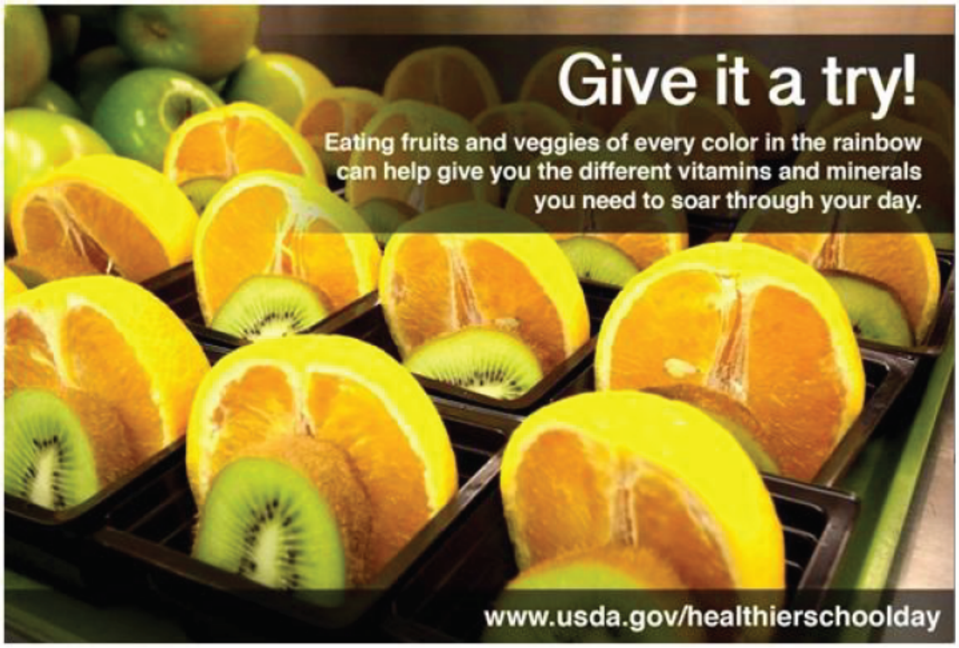


**How would you describe the message above?**

| | Strongly Disagree | Disagree | Neither Agree nor Disagree | Agree | Strongly Agree |
|---|---|---|---|---|---|
| Helpful | ○ | ○ | ○ | ○ | ○ |
| Informative | ○ | ○ | ○ | ○ | ○ |
| Important | ○ | ○ | ○ | ○ | ○ |
| Motivating | ○ | ○ | ○ | ○ | ○ |
| Credible Information | ○ | ○ | ○ | ○ | ○ |
| Appropriate | ○ | ○ | ○ | ○ | ○ |
| Stigmatizing | ○ | ○ | ○ | ○ | ○ |
| Useful | ○ | ○ | ○ | ○ | ○ |

**How would you describe the photo above?**

| | Strongly Disagree | Disagree | Neither Agree nor Disagree | Agree | Strongly Agree |
|---|---|---|---|---|---|
| Helpful | ○ | ○ | ○ | ○ | ○ |
| Informative | ○ | ○ | ○ | ○ | ○ |
| Important | ○ | ○ | ○ | ○ | ○ |
| Motivating | ○ | ○ | ○ | ○ | ○ |
| Credible Information | ○ | ○ | ○ | ○ | ○ |
| Appropriate | ○ | ○ | ○ | ○ | ○ |
| Stigmatizing | ○ | ○ | ○ | ○ | ○ |
| Useful | ○ | ○ | ○ | ○ | ○ |

**Are you more likely to eat more fruits and vegetables with different colors after seeing this image?**

○ Definitely will not

○ Probably will not

○ Don't know

- ○ Probably will
- ○ Definitely will

**Please indicate how much you agree with the following statement: I intend to eat more fruits and vegetables with various colors after seeing this image?**

- ○ Strongly Disagree
- ○ Disagree
- ○ Neither Agree nor Disagree
- ○ Agree
- ○ Strongly Agree

**If you would try to eat more fruits and vegetables with different colors, would you be able to do this?**

- ○ Definitely Not
- ○ Probably not
- ○ Maybe
- ○ Probably yes
- ○ Definitely yes

**Use the image below to answer the following questions**

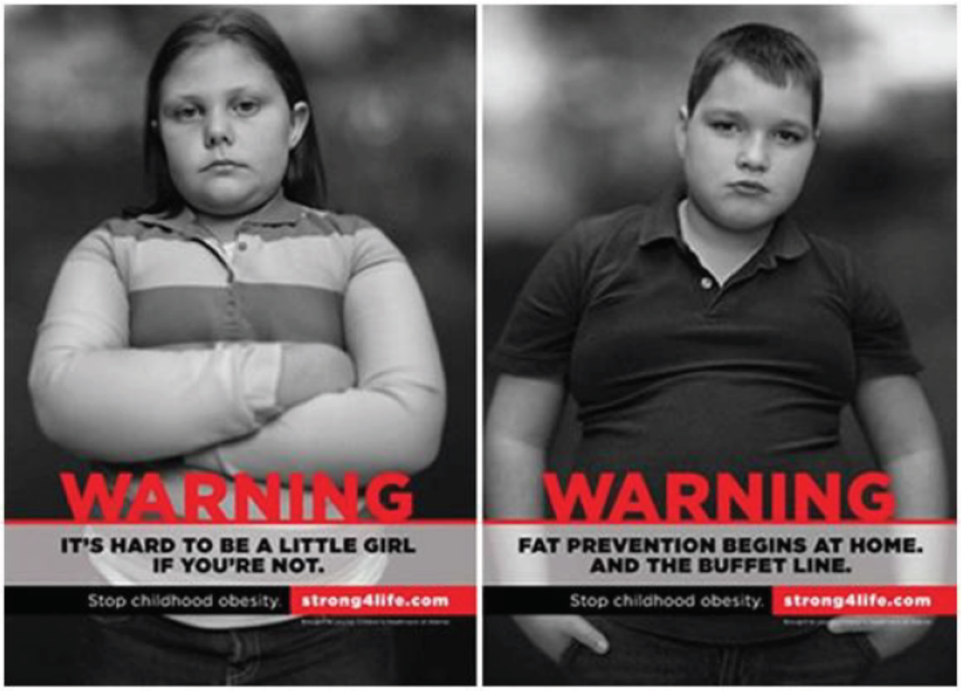


**How would you describe the message above?**

| | Strongly Disagree | Disagree | Neither Agree nor Disagree | Agree | Strongly Agree |
|---|---|---|---|---|---|
| Helpful | ○ | ○ | ○ | ○ | ○ |
| Informative | ○ | ○ | ○ | ○ | ○ |
| Important | ○ | ○ | ○ | ○ | ○ |

| | Strongly Disagree | Disagree | Neither Agree nor Disagree | Agree | Strongly Agree |
|---|---|---|---|---|---|
| Motivating | ○ | ○ | ○ | ○ | ○ |
| Credible Information | ○ | ○ | ○ | ○ | ○ |
| Appropriate | ○ | ○ | ○ | ○ | ○ |
| Stigmatizing | ○ | ○ | ○ | ○ | ○ |
| Useful | ○ | ○ | ○ | ○ | ○ |

**How would you describe the photo above?**

| | Strongly Disagree | Disagree | Neither Agree nor Disagree | Agree | Strongly Agree |
|---|---|---|---|---|---|
| Helpful | ○ | ○ | ○ | ○ | ○ |
| Informative | ○ | ○ | ○ | ○ | ○ |
| Important | ○ | ○ | ○ | ○ | ○ |
| Motivating | ○ | ○ | ○ | ○ | ○ |
| Credible Information | ○ | ○ | ○ | ○ | ○ |
| Appropriate | ○ | ○ | ○ | ○ | ○ |
| Stigmatizing | ○ | ○ | ○ | ○ | ○ |
| Useful | ○ | ○ | ○ | ○ | ○ |

**Are you more likely to take preventive steps to prevent obesity after seeing this image?**

- ○ Definitely will not
- ○ Probably will not
- ○ Don't know
- ○ Probably will
- ○ Definitely will

**Please indicate how much you agree with the following statement: I intend to take steps to prevent obesity after seeing this image.**

- ○ Strongly Disagree
- ○ Disagree
- ○ Neither Agree nor Disagree
- ○ Agree
- ○ Strongly Agree

**If you would try to take steps to prevent obesity, would you be able to do this?**

- ○ Definitely Not
- ○ Probably not
- ○ Maybe
- ○ Probably yes
- ○ Definitely yes

**Use the image below to answer the following questions**

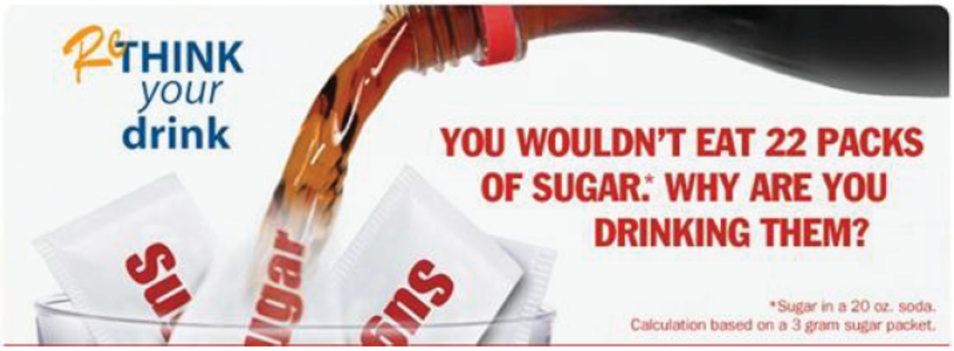


**How would you describe the message above?**

| | Strongly Disagree | Disagree | Neither Agree nor Disagree | Agree | Strongly Agree |
|---|---|---|---|---|---|
| Helpful | ○ | ○ | ○ | ○ | ○ |
| Informative | ○ | ○ | ○ | ○ | ○ |
| Important | ○ | ○ | ○ | ○ | ○ |
| Motivating | ○ | ○ | ○ | ○ | ○ |
| Credible Information | ○ | ○ | ○ | ○ | ○ |
| Appropriate | ○ | ○ | ○ | ○ | ○ |
| Stigmatizing | ○ | ○ | ○ | ○ | ○ |
| Useful | ○ | ○ | ○ | ○ | ○ |

**How would you describe the photo above?**

| | Strongly Disagree | Disagree | Neither Agree nor Disagree | Agree | Strongly Agree |
|---|---|---|---|---|---|
| Helpful | ○ | ○ | ○ | ○ | ○ |
| Informative | ○ | ○ | ○ | ○ | ○ |
| Important | ○ | ○ | ○ | ○ | ○ |
| Motivating | ○ | ○ | ○ | ○ | ○ |
| Credible Information | ○ | ○ | ○ | ○ | ○ |
| Appropriate | ○ | ○ | ○ | ○ | ○ |
| Stigmatizing | ○ | ○ | ○ | ○ | ○ |
| Useful | ○ | ○ | ○ | ○ | ○ |

**Are you more likely to think about changing what you drink after seeing this image?**

- ○ Definitely will not
- ○ Probably will not
- ○ Don't know
- ○ Probably will
- ○ Definitely will

**Please indicate how much you agree with the following statement: I intend to change what I'm drinking for a healthier option after seeing this image.**

○

Strongly Disagree

- Disagree
- Neither Agree nor Disagree
- Agree
- Strongly Agree

**If you would try to change what you drink, would you be able to do this?**

- Definitely Not
- Probably not
- Maybe
- Probably yes
- Definitely yes

**Use the image below to answer the following questions**

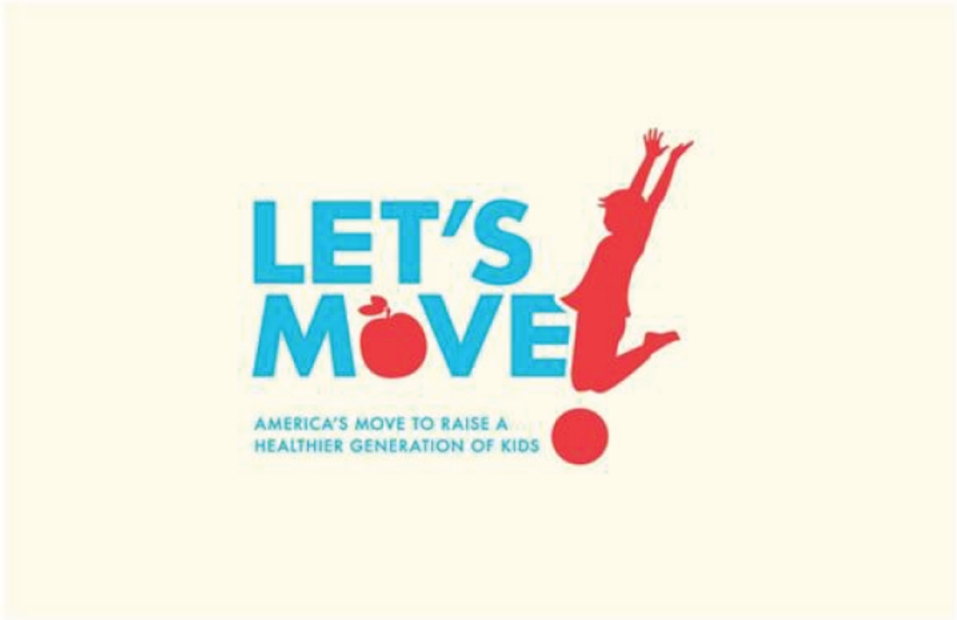


**How would you describe the message above?**

| | Strongly Disagree | Disagree | Neither Agree nor Disagree | Agree | Strongly Agree |
|---|---|---|---|---|---|
| Helpful | ○ | ○ | ○ | ○ | ○ |
| Informative | ○ | ○ | ○ | ○ | ○ |
| Important | ○ | ○ | ○ | ○ | ○ |
| Motivating | ○ | ○ | ○ | ○ | ○ |
| Credible Information | ○ | ○ | ○ | ○ | ○ |
| Appropriate | ○ | ○ | ○ | ○ | ○ |
| Stigmatizing | ○ | ○ | ○ | ○ | ○ |
| Useful | ○ | ○ | ○ | ○ | ○ |

**How would you describe the photo above?**

| | Strongly Disagree | Disagree | Neither Agree nor Disagree | Agree | Strongly Agree |
|---|---|---|---|---|---|
| Helpful | ○ | ○ | ○ | ○ | ○ |
| Informative | ○ | ○ | ○ | ○ | ○ |
| Important | ○ | ○ | ○ | ○ | ○ |
| Motivating | ○ | ○ | ○ | ○ | ○ |
| Credible Information | ○ | ○ | ○ | ○ | ○ |
| Appropriate | ○ | ○ | ○ | ○ | ○ |
| Stigmatizing | ○ | ○ | ○ | ○ | ○ |
| Useful | ○ | ○ | ○ | ○ | ○ |

**Are you more likely to get moving after seeing this image?**

- ○ Definitely will not
- ○ Probably will not
- ○ Don't know
- ○ Probably will
- ○ Definitely will

**Please indicate how much you agree with the following statement: I intend to start moving more after seeing this image.**

- ○ Strongly Disagree
- ○ Disagree
- ○ Neither Agree nor Disagree
- ○ Agree
- ○ Strongly Agree

**If you would try to move more each day, would you be able to do this?**

- ○ Definitely Not
- ○ Probably not
- ○ Maybe
- ○ Probably yes
- ○ Definitely yes

**Use the image below to answer the following questions**

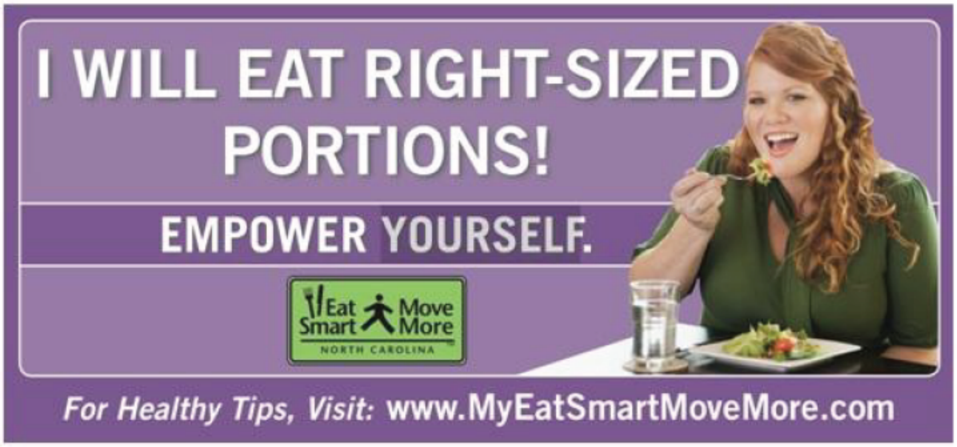


**How would you describe the message above?**

| | Strongly Disagree | Disagree | Neither Agree nor Disagree | Agree | Strongly Agree |
|---|---|---|---|---|---|
| Helpful | ○ | ○ | ○ | ○ | ○ |
| Informative | ○ | ○ | ○ | ○ | ○ |
| Important | ○ | ○ | ○ | ○ | ○ |
| Motivating | ○ | ○ | ○ | ○ | ○ |
| Credible Information | ○ | ○ | ○ | ○ | ○ |
| Appropriate | ○ | ○ | ○ | ○ | ○ |
| Stigmatizing | ○ | ○ | ○ | ○ | ○ |
| Useful | ○ | ○ | ○ | ○ | ○ |

**How would you describe the photo above?**

| | Strongly Disagree | Disagree | Neither Agree nor Disagree | Agree | Strongly Agree |
|---|---|---|---|---|---|
| Helpful | ○ | ○ | ○ | ○ | ○ |
| Informative | ○ | ○ | ○ | ○ | ○ |
| Important | ○ | ○ | ○ | ○ | ○ |
| Motivating | ○ | ○ | ○ | ○ | ○ |
| Credible Information | ○ | ○ | ○ | ○ | ○ |
| Appropriate | ○ | ○ | ○ | ○ | ○ |
| Stigmatizing | ○ | ○ | ○ | ○ | ○ |
| Useful | ○ | ○ | ○ | ○ | ○ |

**Are you more likely to eat right-sized portions after seeing this image?**

- ○ Definitely will not
- ○ Probably will not
- ○ Don't know
- ○ Probably will
- ○ Definitely will

**Please indicate how much you agree with the following statement: I intend to start eating right-sized portions after seeing this image.**

- Strongly Disagree
- Disagree
- Neither Agree nor Disagree
- Agree
- Strongly Agree

**If you would try eat right-sized portions, would you be able to do this?**

- Definitely Not
- Probably not
- Maybe
- Probably yes
- Definitely yes

**Use the image below to answer the following questions**

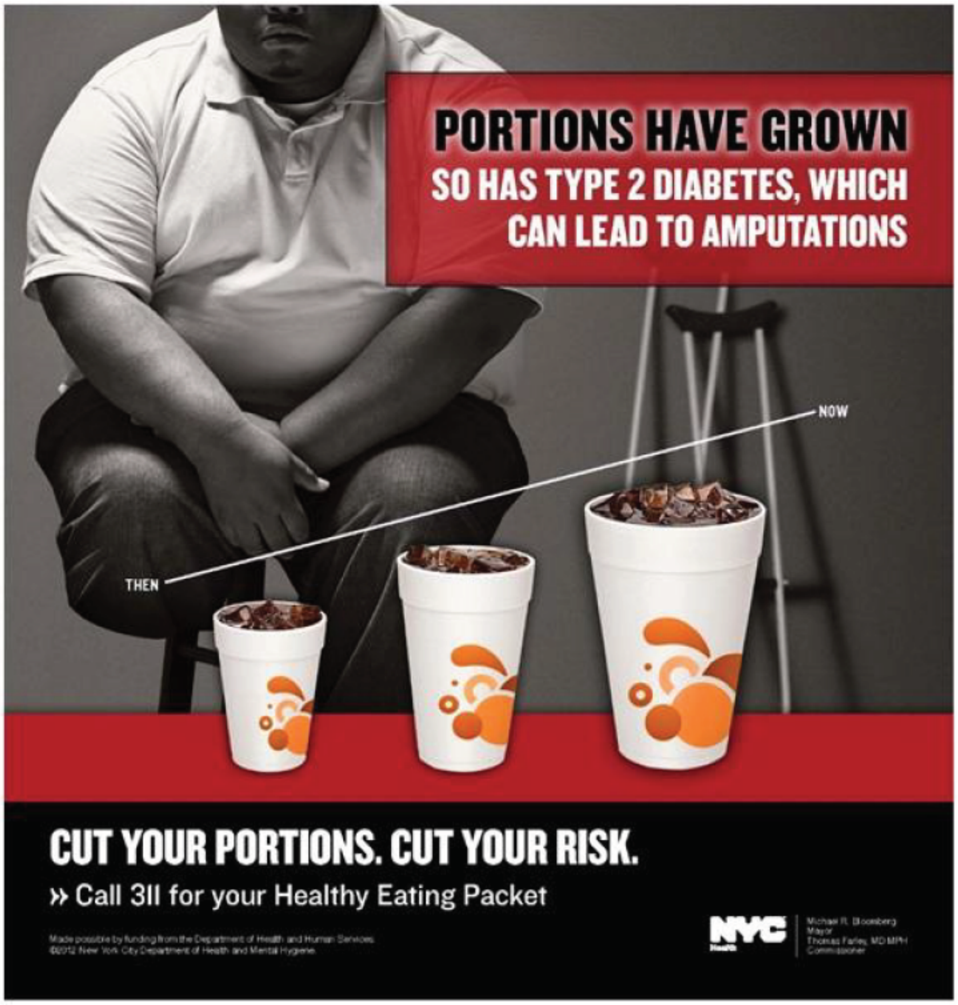


**How would you describe the message above?**

| | Strongly Disagree | Disagree | Neither Agree nor Disagree | Agree | Strongly Agree |
|---|---|---|---|---|---|
| Helpful | ○ | ○ | ○ | ○ | ○ |

| | Strongly Disagree | Disagree | Neither Agree nor Disagree | Agree | Strongly Agree |
|---|---|---|---|---|---|
| Informative | ○ | ○ | ○ | ○ | ○ |
| Important | ○ | ○ | ○ | ○ | ○ |
| Motivating | ○ | ○ | ○ | ○ | ○ |
| Credible Information | ○ | ○ | ○ | ○ | ○ |
| Appropriate | ○ | ○ | ○ | ○ | ○ |
| Stigmatizing | ○ | ○ | ○ | ○ | ○ |
| Useful | ○ | ○ | ○ | ○ | ○ |

**How would you describe the photo above?**

| | Strongly Disagree | Disagree | Neither Agree nor Disagree | Agree | Strongly Agree |
|---|---|---|---|---|---|
| Helpful | ○ | ○ | ○ | ○ | ○ |
| Informative | ○ | ○ | ○ | ○ | ○ |
| Important | ○ | ○ | ○ | ○ | ○ |
| Motivating | ○ | ○ | ○ | ○ | ○ |
| Credible Information | ○ | ○ | ○ | ○ | ○ |
| Appropriate | ○ | ○ | ○ | ○ | ○ |
| Stigmatizing | ○ | ○ | ○ | ○ | ○ |
| Useful | ○ | ○ | ○ | ○ | ○ |

**Are you more likely to cut your portion sizes after seeing this image?**

- ○ Definitely will not
- ○ Probably will not
- ○ Don't know
- ○ Probably will
- ○ Definitely will

**Please indicate how much you agree with the following statement: I intend to cut my portion sizes after seeing this image.**

- ○ Strongly Disagree
- ○ Disagree
- ○ Neither Agree nor Disagree
- ○ Agree
- ○ Strongly Agree

**If you would try eat smaller portions, would you be able to do this?**

- ○ Definitely Not
- ○ Probably not
- ○ Maybe
- ○ Probably yes

○ Definitely yes

**Use the image below to answer the following questions**

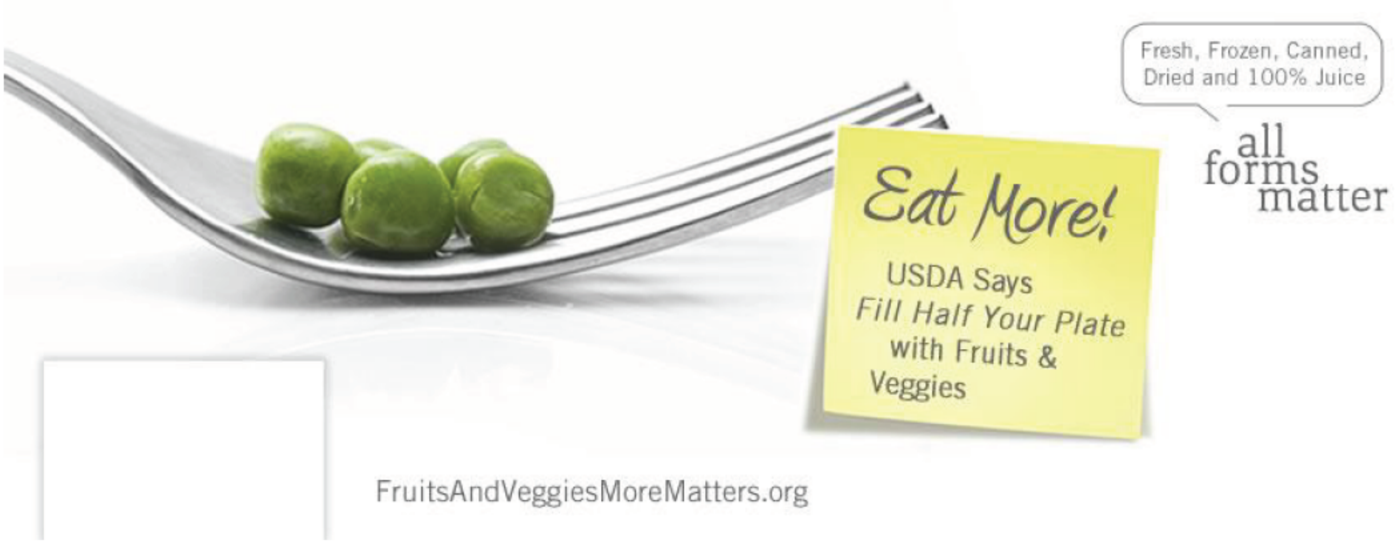


**How would you describe the message above?**

| | Strongly Disagree | Disagree | Neither Agree nor Disagree | Agree | Strongly Agree |
|---|---|---|---|---|---|
| Helpful | ○ | ○ | ○ | ○ | ○ |
| Informative | ○ | ○ | ○ | ○ | ○ |
| Important | ○ | ○ | ○ | ○ | ○ |
| Motivating | ○ | ○ | ○ | ○ | ○ |
| Credible Information | ○ | ○ | ○ | ○ | ○ |
| Appropriate | ○ | ○ | ○ | ○ | ○ |
| Stigmatizing | ○ | ○ | ○ | ○ | ○ |
| Useful | ○ | ○ | ○ | ○ | ○ |

**How would you describe the photo above?**

| | Strongly Disagree | Disagree | Neither Agree nor Disagree | Agree | Strongly Agree |
|---|---|---|---|---|---|
| Helpful | ○ | ○ | ○ | ○ | ○ |
| Informative | ○ | ○ | ○ | ○ | ○ |
| Important | ○ | ○ | ○ | ○ | ○ |
| Motivating | ○ | ○ | ○ | ○ | ○ |
| Credible Information | ○ | ○ | ○ | ○ | ○ |
| Appropriate | ○ | ○ | ○ | ○ | ○ |
| Stigmatizing | ○ | ○ | ○ | ○ | ○ |
| Useful | ○ | ○ | ○ | ○ | ○ |

**Are you more likely to make your plate half fruits and veggies after seeing this image?**

○ Definitely will not

○ Probably will not

○

Don't know

- Probably will
- Definitely will

**Please indicate how much you agree with the following statement: I intend to make half my plate fruits and veggies after seeing this image.**

- Strongly Disagree
- Disagree
- Neither Agree nor Disagree
- Agree
- Strongly Agree

**If you would try to make half your plate fruits and veggies, would you be able to do this?**

- Definitely Not
- Probably not
- Maybe
- Probably yes
- Definitely yes

**Use the image below to answer the following questions**

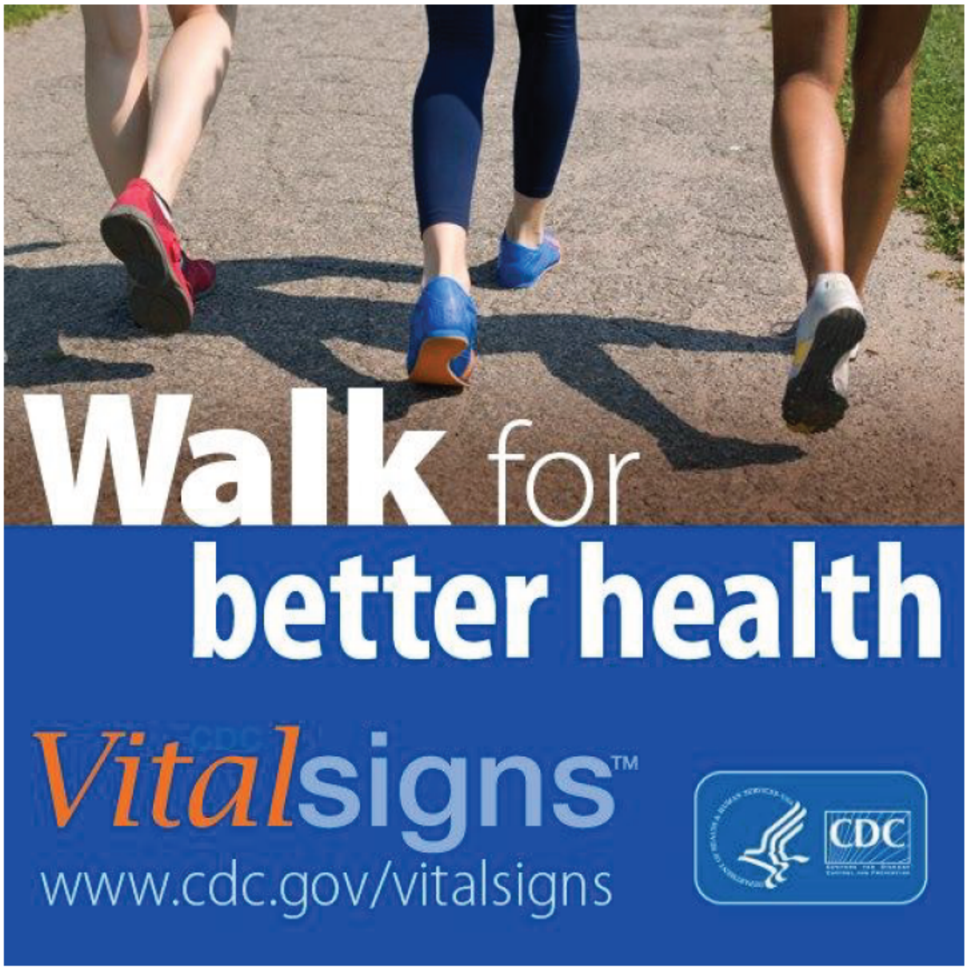


**How would you describe the message above?**

| | Strongly Disagree | Disagree | Neither Agree nor Disagree | Agree | Strongly Agree |
|---|---|---|---|---|---|
| Helpful | ◯ | ◯ | ◯ | ◯ | ◯ |
| Informative | ◯ | ◯ | ◯ | ◯ | ◯ |
| Important | ◯ | ◯ | ◯ | ◯ | ◯ |
| Motivating | ◯ | ◯ | ◯ | ◯ | ◯ |
| Credible Information | ◯ | ◯ | ◯ | ◯ | ◯ |
| Appropriate | ◯ | ◯ | ◯ | ◯ | ◯ |
| Stigmatizing | ◯ | ◯ | ◯ | ◯ | ◯ |
| Useful | ◯ | ◯ | ◯ | ◯ | ◯ |

**How would you describe the photo above?**

| | Strongly Disagree | Disagree | Neither Agree nor Disagree | Agree | Strongly Agree |
|---|---|---|---|---|---|
| Helpful | ◯ | ◯ | ◯ | ◯ | ◯ |
| Informative | ◯ | ◯ | ◯ | ◯ | ◯ |
| Important | ◯ | ◯ | ◯ | ◯ | ◯ |
| Motivating | ◯ | ◯ | ◯ | ◯ | ◯ |
| Credible Information | ◯ | ◯ | ◯ | ◯ | ◯ |
| Appropriate | ◯ | ◯ | ◯ | ◯ | ◯ |
| Stigmatizing | ◯ | ◯ | ◯ | ◯ | ◯ |

| | Strongly Disagree | Disagree | Neither Agree nor Disagree | Agree | Strongly Agree |
|---|---|---|---|---|---|
| Useful | ○ | ○ | ○ | ○ | ○ |

**Are you more likely to start or walk more after seeing this image?**

- ○ Definitely will not
- ○ Probably will not
- ○ Don't know
- ○ Probably will
- ○ Definitely will

**Please indicate how much you agree with the following statement: I intend to start walking or walk more after seeing this image.**

- ○ Strongly Disagree
- ○ Disagree
- ○ Neither Agree nor Disagree
- ○ Agree
- ○ Strongly Agree

**If you would try to start or walk more, would you be able to do this?**

- ○ Definitely Not
- ○ Probably not
- ○ Maybe
- ○ Probably yes
- ○ Definitely yes

**Use the image below to answer the following questions**

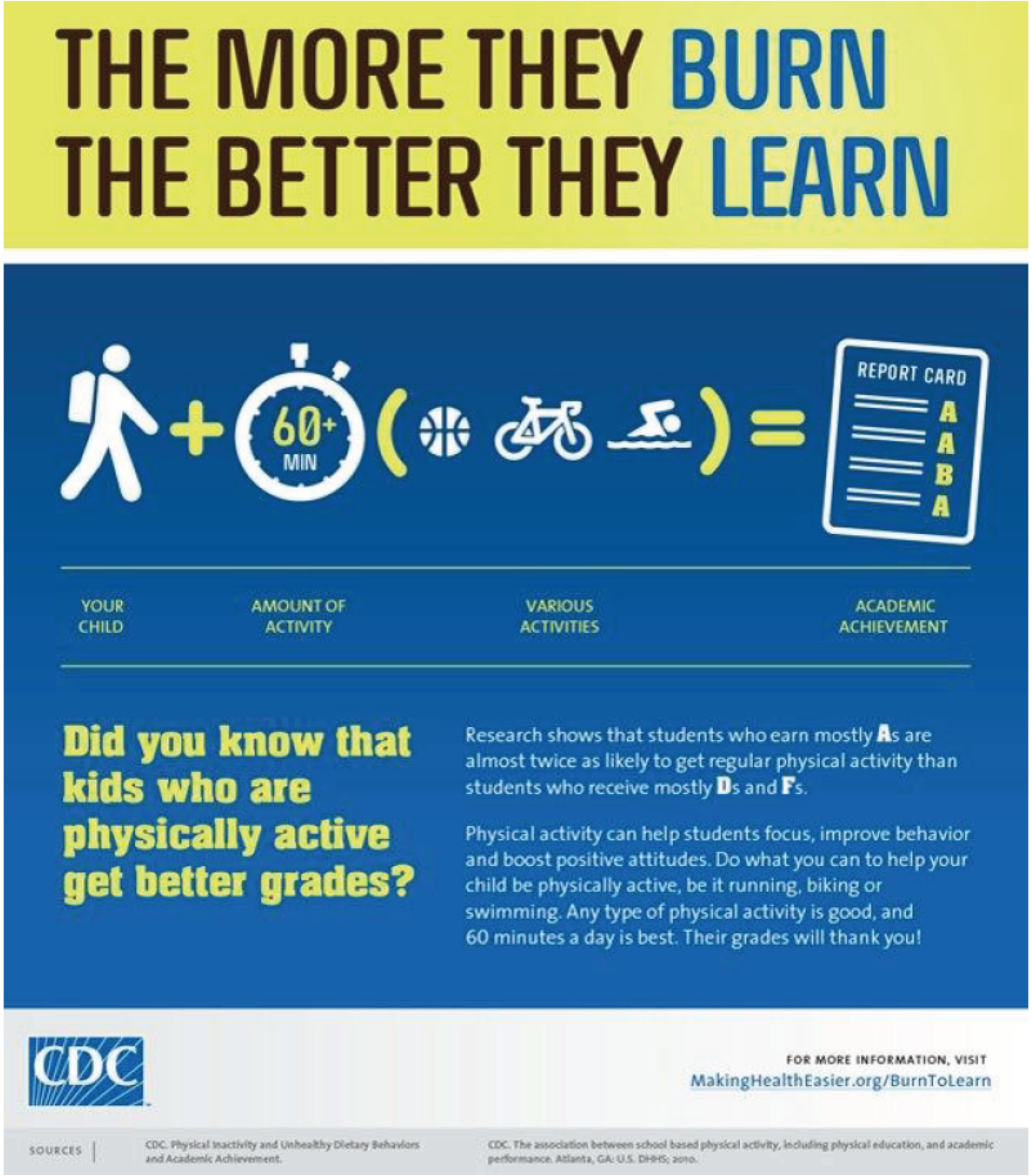


**How would you describe the message above?**

| | Strongly Disagree | Disagree | Neither Agree nor Disagree | Agree | Strongly Agree |
|---|---|---|---|---|---|
| Helpful | ○ | ○ | ○ | ○ | ○ |
| Informative | ○ | ○ | ○ | ○ | ○ |
| Important | ○ | ○ | ○ | ○ | ○ |
| Motivating | ○ | ○ | ○ | ○ | ○ |
| Credible Information | ○ | ○ | ○ | ○ | ○ |
| Appropriate | ○ | ○ | ○ | ○ | ○ |
| Stigmatizing | ○ | ○ | ○ | ○ | ○ |
| Useful | ○ | ○ | ○ | ○ | ○ |

**How would you describe the photo above?**

| | Strongly Disagree | Disagree | Neither Agree nor Disagree | Agree | Strongly Agree |
|---|---|---|---|---|---|
| Helpful | ○ | ○ | ○ | ○ | ○ |

| | Strongly Disagree | Disagree | Neither Agree nor Disagree | Agree | Strongly Agree |
|---|---|---|---|---|---|
| Informative | ○ | ○ | ○ | ○ | ○ |
| Important | ○ | ○ | ○ | ○ | ○ |
| Motivating | ○ | ○ | ○ | ○ | ○ |
| Credible Information | ○ | ○ | ○ | ○ | ○ |
| Appropriate | ○ | ○ | ○ | ○ | ○ |
| Stigmatizing | ○ | ○ | ○ | ○ | ○ |
| Useful | ○ | ○ | ○ | ○ | ○ |

**Are you more likely to be physical active after seeing this image?**

- ○ Definitely will not
- ○ Probably will not
- ○ Don't know
- ○ Probably will
- ○ Definitely will

**Please indicate how much you agree with the following statement: I intend to be physical active after seeing this image.**

- ○ Strongly Disagree
- ○ Disagree
- ○ Neither Agree nor Disagree
- ○ Agree
- ○ Strongly Agree

**If you would try to be physical active, would you be able to do this?**

- ○ Definitely Not
- ○ Probably not
- ○ Maybe
- ○ Probably yes
- ○ Definitely yes

**Use the image below to answer the following questions**

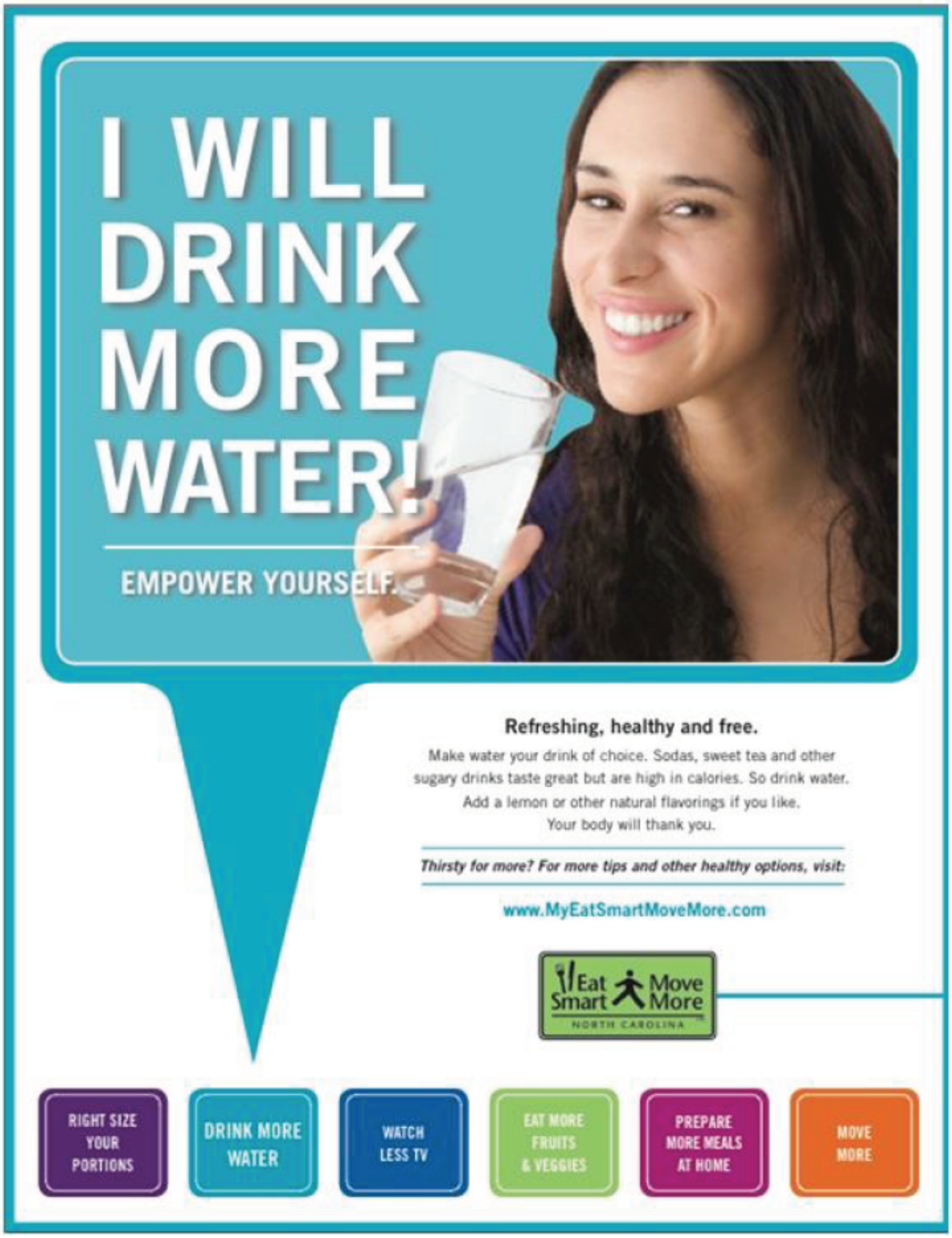


**How would you describe the message above?**

| | Strongly Disagree | Disagree | Neither Agree nor Disagree | Agree | Strongly Agree |
|---|---|---|---|---|---|
| Helpful | ○ | ○ | ○ | ○ | ○ |
| Informative | ○ | ○ | ○ | ○ | ○ |
| Important | ○ | ○ | ○ | ○ | ○ |
| Motivating | ○ | ○ | ○ | ○ | ○ |
| Credible Information | ○ | ○ | ○ | ○ | ○ |
| Appropriate | ○ | ○ | ○ | ○ | ○ |
| Stigmatizing | ○ | ○ | ○ | ○ | ○ |
| Useful | ○ | ○ | ○ | ○ | ○ |

**How would you describe the photo above?**

| | Strongly Disagree | Disagree | Neither Agree nor Disagree | Agree | Strongly Agree |
|---|---|---|---|---|---|

| | Strongly Disagree | Disagree | Neither Agree nor Disagree | Agree | Strongly Agree |
|---|---|---|---|---|---|
| Helpful | ○ | ○ | ○ | ○ | ○ |
| Informative | ○ | ○ | ○ | ○ | ○ |
| Important | ○ | ○ | ○ | ○ | ○ |
| Motivating | ○ | ○ | ○ | ○ | ○ |
| Credible Information | ○ | ○ | ○ | ○ | ○ |
| Appropriate | ○ | ○ | ○ | ○ | ○ |
| Stigmatizing | ○ | ○ | ○ | ○ | ○ |
| Useful | ○ | ○ | ○ | ○ | ○ |

**Are you more likely to drink more water after seeing this image?**

- ○ Definitely will not
- ○ Probably will not
- ○ Don't know
- ○ Probably will
- ○ Definitely will

**Please indicate how much you agree with the following statement: I intend to drink more water after seeing this image.**

- ○ Strongly Disagree
- ○ Disagree
- ○ Neither Agree nor Disagree
- ○ Agree
- ○ Strongly Agree

**If you would try to drink more water, would you be able to do this?**

- ○ Definitely Not
- ○ Probably not
- ○ Maybe
- ○ Probably yes
- ○ Definitely yes

**Use the image below to answer the following questions**

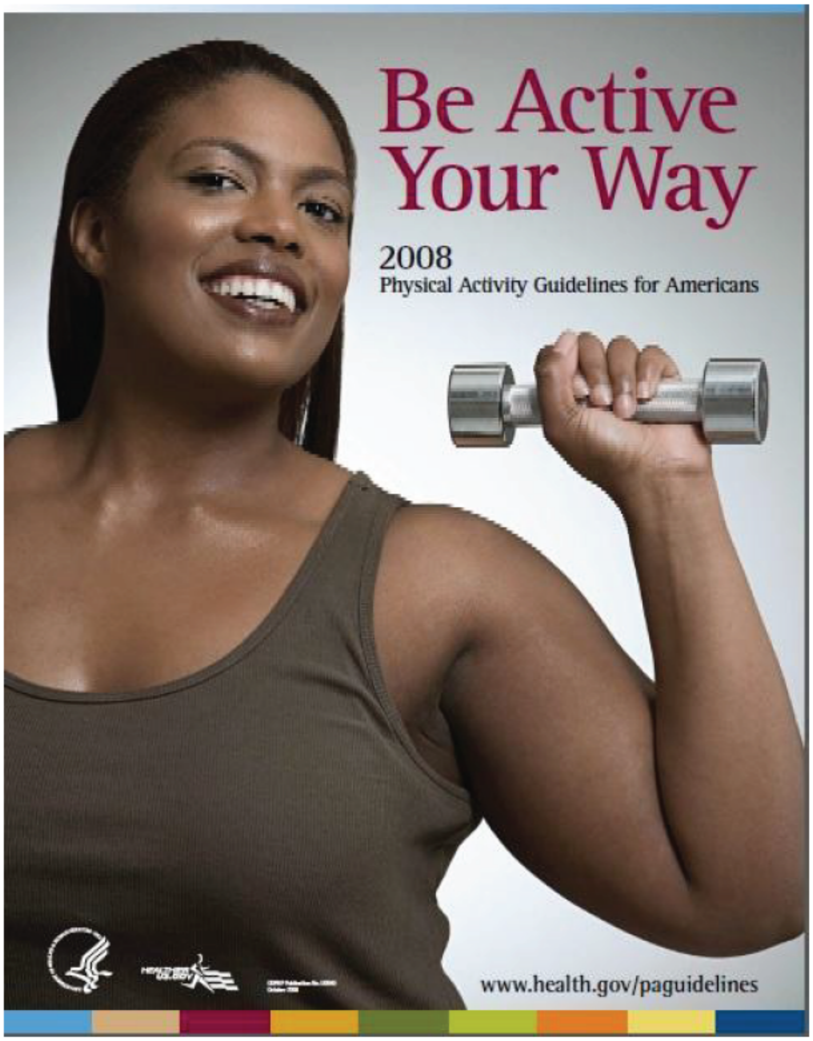


**How would you describe the message above?**

| | Strongly Disagree | Disagree | Neither Agree nor Disagree | Agree | Strongly Agree |
|---|---|---|---|---|---|
| Helpful | ○ | ○ | ○ | ○ | ○ |
| Informative | ○ | ○ | ○ | ○ | ○ |
| Important | ○ | ○ | ○ | ○ | ○ |
| Motivating | ○ | ○ | ○ | ○ | ○ |
| Credible Information | ○ | ○ | ○ | ○ | ○ |
| Appropriate | ○ | ○ | ○ | ○ | ○ |
| Stigmatizing | ○ | ○ | ○ | ○ | ○ |
| Useful | ○ | ○ | ○ | ○ | ○ |

**How would you describe the photo above?**

| | Strongly Disagree | Disagree | Neither Agree nor Disagree | Agree | Strongly Agree |
|---|---|---|---|---|---|
| Helpful | ○ | ○ | ○ | ○ | ○ |
| Informative | ○ | ○ | ○ | ○ | ○ |
| Important | ○ | ○ | ○ | ○ | ○ |
| Motivating | ○ | ○ | ○ | ○ | ○ |
| Credible Information | ○ | ○ | ○ | ○ | ○ |

| | Strongly Disagree | Disagree | Neither Agree nor Disagree | Agree | Strongly Agree |
|---|---|---|---|---|---|
| Appropriate | ○ | ○ | ○ | ○ | ○ |
| Stigmatizing | ○ | ○ | ○ | ○ | ○ |
| Useful | ○ | ○ | ○ | ○ | ○ |

**Are you more likely to be active after seeing this image?**

- ○ Definitely will not
- ○ Probably will not
- ○ Don't know
- ○ Probably will
- ○ Definitely will

**Please indicate how much you agree with the following statement: I intend to be active after seeing this image.**

- ○ Strongly Disagree
- ○ Disagree
- ○ Neither Agree nor Disagree
- ○ Agree
- ○ Strongly Agree

**If you would try to be active, would you be able to do this?**

- ○ Definitely Not
- ○ Probably not
- ○ Maybe
- ○ Probably yes
- ○ Definitely yes

**Use the image below to answer the following questions**

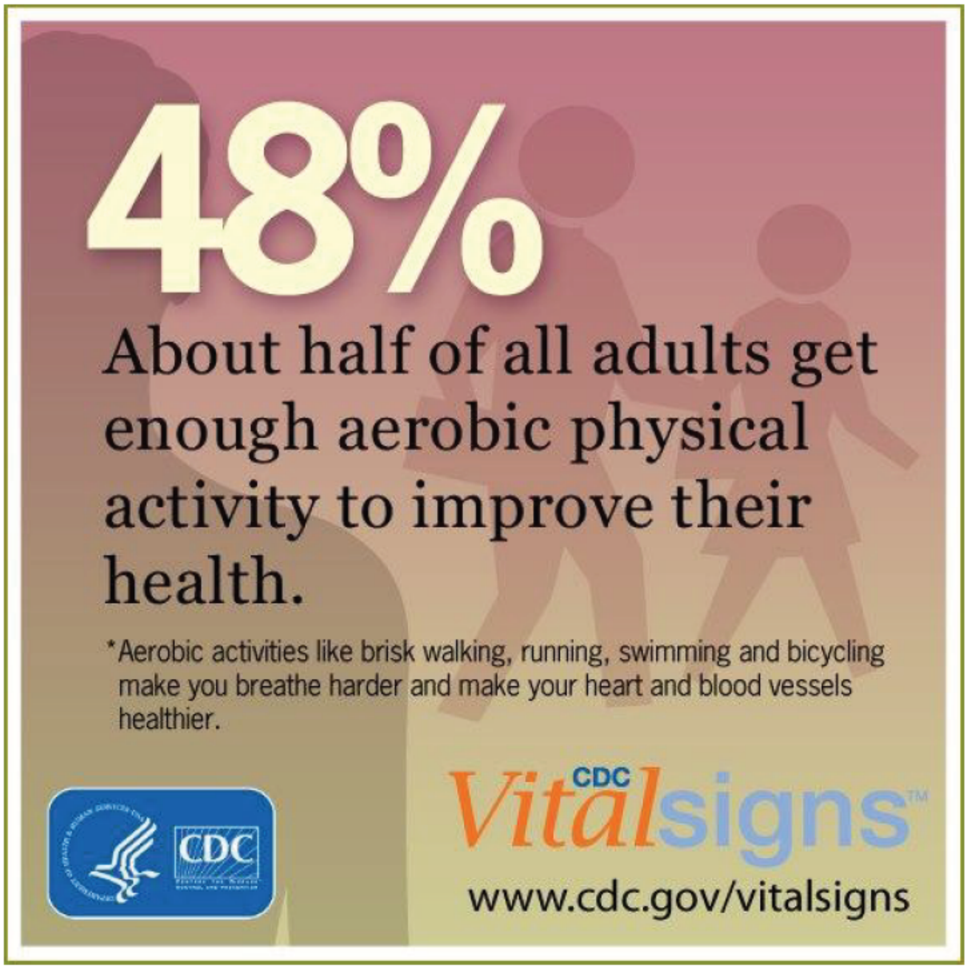


**How would you describe the message above?**

| | Strongly Disagree | Disagree | Neither Agree nor Disagree | Agree | Strongly Agree |
|---|---|---|---|---|---|
| Helpful | ○ | ○ | ○ | ○ | ○ |
| Informative | ○ | ○ | ○ | ○ | ○ |
| Important | ○ | ○ | ○ | ○ | ○ |
| Motivating | ○ | ○ | ○ | ○ | ○ |
| Credible Information | ○ | ○ | ○ | ○ | ○ |
| Appropriate | ○ | ○ | ○ | ○ | ○ |
| Stigmatizing | ○ | ○ | ○ | ○ | ○ |
| Useful | ○ | ○ | ○ | ○ | ○ |

**How would you describe the photo above?**

| | Strongly Disagree | Disagree | Neither Agree nor Disagree | Agree | Strongly Agree |
|---|---|---|---|---|---|
| Helpful | ○ | ○ | ○ | ○ | ○ |
| Informative | ○ | ○ | ○ | ○ | ○ |
| Important | ○ | ○ | ○ | ○ | ○ |
| Motivating | ○ | ○ | ○ | ○ | ○ |
| Credible Information | ○ | ○ | ○ | ○ | ○ |
| Appropriate | ○ | ○ | ○ | ○ | ○ |
| Stigmatizing | ○ | ○ | ○ | ○ | ○ |

| | Strongly Disagree | Disagree | Neither Agree nor Disagree | Agree | Strongly Agree |
|---|---|---|---|---|---|
| Useful | ○ | ○ | ○ | ○ | ○ |

**Are you more likely to participate in aerobic exercise after seeing this image?**

- ○ Definitely will not
- ○ Probably will not
- ○ Don't know
- ○ Probably will
- ○ Definitely will

**Please indicate how much you agree with the following statement: I intend to engage in aerobic exercise after seeing this image.**

- ○ Strongly Disagree
- ○ Disagree
- ○ Neither Agree nor Disagree
- ○ Agree
- ○ Strongly Agree

**If you would try to engage in aerobic exercise, would you be able to do this?**

- ○ Definitely Not
- ○ Probably not
- ○ Maybe
- ○ Probably yes
- ○ Definitely yes

**Use the image below to answer the following questions**

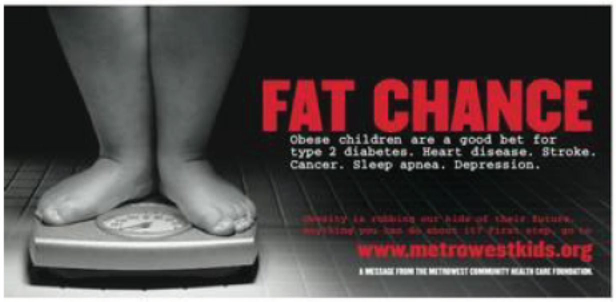


**How would you describe the message above?**

| | Strongly Disagree | Disagree | Neither Agree nor Disagree | Agree | Strongly Agree |
|---|---|---|---|---|---|
| Helpful | ○ | ○ | ○ | ○ | ○ |
| Informative | ○ | ○ | ○ | ○ | ○ |
| Important | ○ | ○ | ○ | ○ | ○ |

| | Strongly Disagree | Disagree | Neither Agree nor Disagree | Agree | Strongly Agree |
|---|---|---|---|---|---|
| Motivating | ○ | ○ | ○ | ○ | ○ |
| Credible Information | ○ | ○ | ○ | ○ | ○ |
| Appropriate | ○ | ○ | ○ | ○ | ○ |
| Stigmatizing | ○ | ○ | ○ | ○ | ○ |
| Useful | ○ | ○ | ○ | ○ | ○ |

**How would you describe the photo above?**

| | Strongly Disagree | Disagree | Neither Agree nor Disagree | Agree | Strongly Agree |
|---|---|---|---|---|---|
| Helpful | ○ | ○ | ○ | ○ | ○ |
| Informative | ○ | ○ | ○ | ○ | ○ |
| Important | ○ | ○ | ○ | ○ | ○ |
| Motivating | ○ | ○ | ○ | ○ | ○ |
| Credible Information | ○ | ○ | ○ | ○ | ○ |
| Appropriate | ○ | ○ | ○ | ○ | ○ |
| Stigmatizing | ○ | ○ | ○ | ○ | ○ |
| Useful | ○ | ○ | ○ | ○ | ○ |

**Are you more likely to take steps to prevent obesity after seeing this image?**

○ Definitely will not

○ Probably will not

○ Don't know

○ Probably will

○ Definitely will

**Please indicate how much you agree with the following statement: I intend to take steps to prevent obesity after seeing this image.**

○ Strongly Disagree

○ Disagree

○ Neither Agree nor Disagree

○ Agree

○ Strongly Agree

**If you would try to take steps to prevent obesity, would you be able to do this?**

○ Definitely Not

○ Probably not

○ Maybe

○ Probably yes

○ Definitely yes

Use the image below to answer the following questions

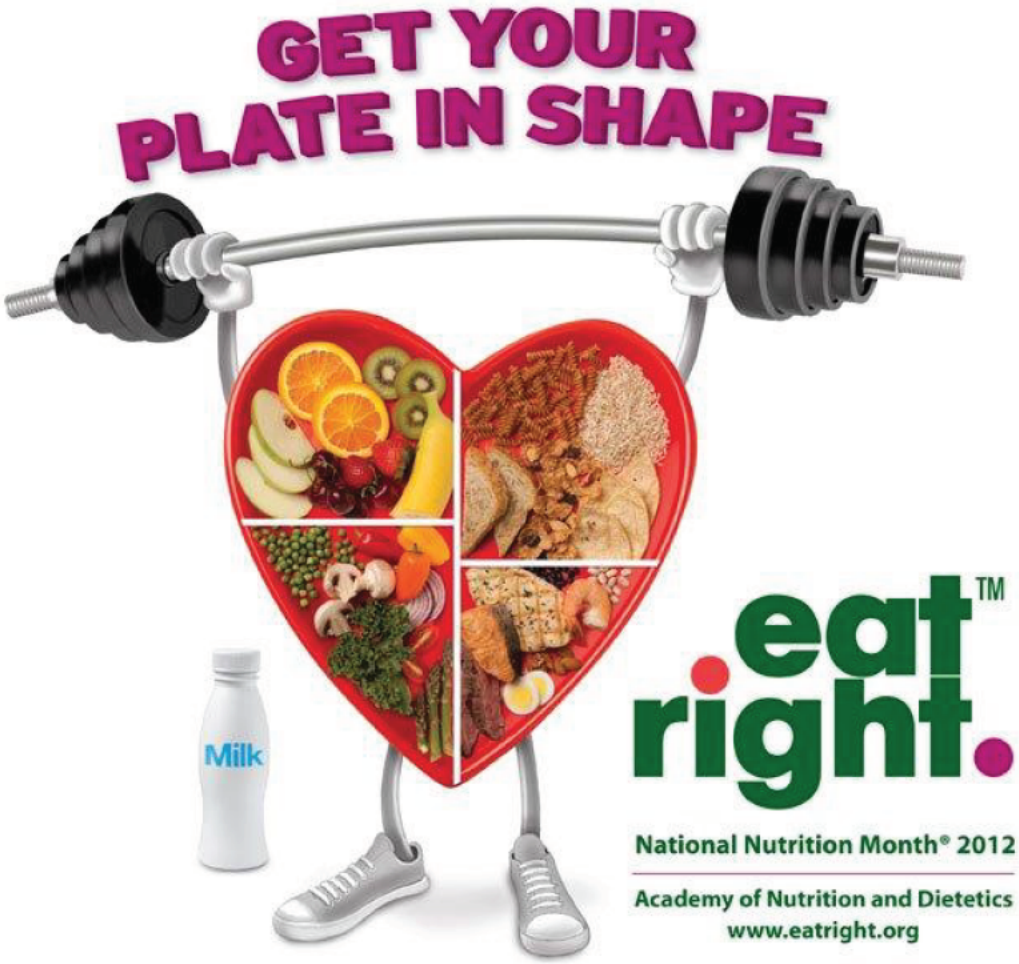


**How would you describe the message above?**

| | Strongly Disagree | Disagree | Neither Agree nor Disagree | Agree | Strongly Agree |
|---|---|---|---|---|---|
| Helpful | ○ | ○ | ○ | ○ | ○ |
| Informative | ○ | ○ | ○ | ○ | ○ |
| Important | ○ | ○ | ○ | ○ | ○ |
| Motivating | ○ | ○ | ○ | ○ | ○ |
| Credible Information | ○ | ○ | ○ | ○ | ○ |
| Appropriate | ○ | ○ | ○ | ○ | ○ |
| Stigmatizing | ○ | ○ | ○ | ○ | ○ |
| Useful | ○ | ○ | ○ | ○ | ○ |

**How would you describe the photo above?**

| | Strongly Disagree | Disagree | Neither Agree nor Disagree | Agree | Strongly Agree |
|---|---|---|---|---|---|
| Helpful | ○ | ○ | ○ | ○ | ○ |
| Informative | ○ | ○ | ○ | ○ | ○ |
| Important | ○ | ○ | ○ | ○ | ○ |
| Motivating | ○ | ○ | ○ | ○ | ○ |
| Credible Information | ○ | ○ | ○ | ○ | ○ |

| | Strongly Disagree | Disagree | Neither Agree nor Disagree | Agree | Strongly Agree |
|---|---|---|---|---|---|
| Appropriate | ○ | ○ | ○ | ○ | ○ |
| Stigmatizing | ○ | ○ | ○ | ○ | ○ |
| Useful | ○ | ○ | ○ | ○ | ○ |

**Are you more likely to change your plate and eat right after seeing this image?**

- ○ Definitely will not
- ○ Probably will not
- ○ Don't know
- ○ Probably will
- ○ Definitely will

**Please indicate how much you agree with the following statement: I intend to change my plate and eat right after seeing this image.**

- ○ Strongly Disagree
- ○ Disagree
- ○ Neither Agree nor Disagree
- ○ Agree
- ○ Strongly Agree

**If you would try to change your plate and eat right, would you be able to do this?**

- ○ Definitely Not
- ○ Probably not
- ○ Maybe
- ○ Probably yes
- ○ Definitely yes

**Use the image below to answer the following questions**

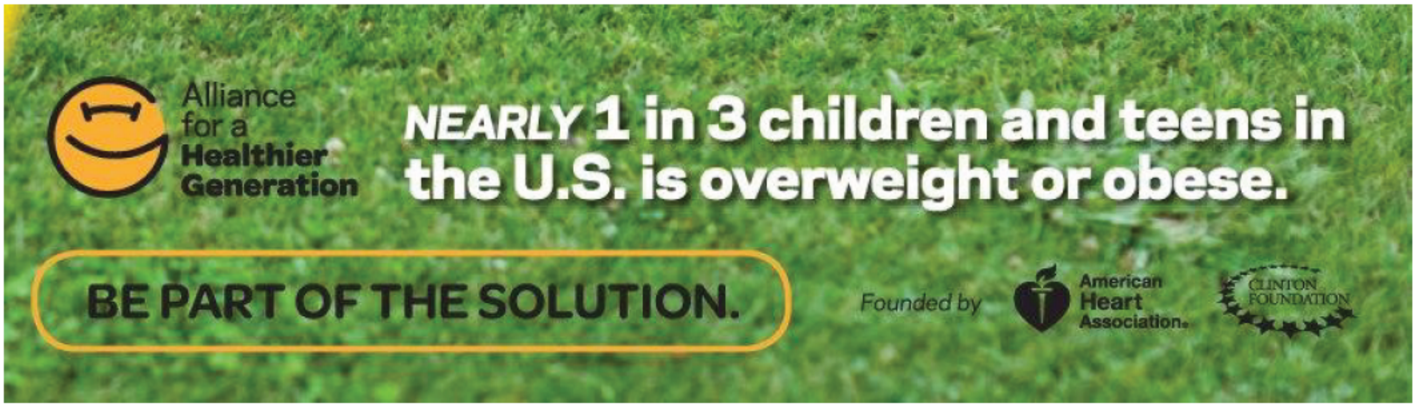


**How would you describe the message above?**

| | Strongly Disagree | Disagree | Neither Agree nor Disagree | Agree | Strongly Agree |
|---|---|---|---|---|---|
| Helpful | ○ | ○ | ○ | ○ | ○ |
| Informative | ○ | ○ | ○ | ○ | ○ |
| Important | ○ | ○ | ○ | ○ | ○ |
| Motivating | ○ | ○ | ○ | ○ | ○ |
| Credible Information | ○ | ○ | ○ | ○ | ○ |
| Appropriate | ○ | ○ | ○ | ○ | ○ |
| Stigmatizing | ○ | ○ | ○ | ○ | ○ |
| Useful | ○ | ○ | ○ | ○ | ○ |

**How would you describe the photo above?**

| | Strongly Disagree | Disagree | Neither Agree nor Disagree | Agree | Strongly Agree |
|---|---|---|---|---|---|
| Helpful | ○ | ○ | ○ | ○ | ○ |
| Informative | ○ | ○ | ○ | ○ | ○ |
| Important | ○ | ○ | ○ | ○ | ○ |
| Motivating | ○ | ○ | ○ | ○ | ○ |
| Credible Information | ○ | ○ | ○ | ○ | ○ |
| Appropriate | ○ | ○ | ○ | ○ | ○ |
| Stigmatizing | ○ | ○ | ○ | ○ | ○ |
| Useful | ○ | ○ | ○ | ○ | ○ |

**Are you more likely to take steps to prevent obesity after seeing this image?**

- ○ Definitely will not
- ○ Probably will not
- ○ Don't know
- ○ Probably will
- ○ Definitely will

**Please indicate how much you agree with the following statement: I intend to take steps to prevent obesity after seeing this image.**

- ○ Strongly Disagree
- ○ Disagree
- ○ Neither Agree nor Disagree
- ○ Agree
- ○ Strongly Agree

**If you would try to take steps to prevent obesity, would you be able to do this?**

- ○ Definitely Not
- ○ Probably not
- ○ Maybe

- Probably yes
- Definitely yes

**What is your sex?**

- Male
- Female
- Intersex/Transexual/Genderqueer

**Which one or more of the following would you say is your race?**

- White
- Black or African American
- Asian
- Native Hawaiian or Other Pacific Islander
- American Indian or Alaska Native
- Other

**What is your classification at the University of Florida?**

- Freshman
- Sophomore
- Junior
- Senior
- Graduate Student
- Professional Student
- Non-degree seeking student
- I am not a student at the University of Florida

**In which college is your current major?**

- College of Agricultural and Life Sciences
- College of Business Administration
- College of Dentistry
- College of Design, Construction, and Planning
- College of Education
- College of Engineering
- College of Fine Arts
- College of Health and Human Performance
- College of Journalism and Communication
- College of Law

College of Liberal Arts and Sciences

- ○ College of Medicine
- ○ College of Nursing
- ○ College of Pharmacy
- ○ College of Public Health and Health Professions
- ○ College of Veterinary Medicine

**What is your age?**

[0] Years

**About how tall are you without shoes? (ft and inches)**

[0] Ft

[0] Inches

**About how much do you weigh without shoes?**

[0] Pounds

**Do you have any suggestions regarding how we can improve this survey?**
