# Supplementary material for: The Health Education Research Experience (HERE) program metadata dataset
Source: Data Brief. 2020 Jan 25;29:105180. doi: 10.1016/j.dib.2020.105180 (PMC7100622; doi:10.1016/j.dib.2020.105180)
Supplement: Multimedia component 11 [file mmc11.pdf]

[redacted]

Informed Consent
Protocol Title: Survey on Distracted Driving Attitudes and Behaviors
Please read this consent document carefully before you decide to participate in this study.

Purpose of the research study:
This study addresses how university students perceive distracted driving. The purpose of this study is to examine University of Florida students' attitudes and behaviors regarding distracted driving. This research will supplement the current health education literature and injury prevention literature regarding college students at American universities. We are also interested in how you complete this survey (e.g. on your computer, your phone, or a tablet computer like an iPad). As such, the survey program, Qualtrics, will collect technical information addressed in the Confidentiality Section below.

Role of Research in HSC 3102:
One of the primary responsibilities of Certified Health Education Specialists is to *Conduct Evaluation and Research Related to Health Education*. As such, one of the goals of HSC 3102 – Personal and Family Health -- is to familiarize you with the research process in health education. To familiarize you with the research process in health education, we have created online surveys and introspective journal entries related to the content in each module.

Earning Health Education Research Experience Points:
This module includes a survey AND a journal entry. For this module, you may choose to participate in EITHER activity to receive your Health Education Research Experience points (5 points). Deadlines for the this module's survey participation or journal entry are listed in the Sakai course website and correspond with the deadline for completing this module.

What you will be asked to do in the study:
You will be asked to take a 75-item questionnaire online through Qualtrics. In this study you will be asked about your attitudes regarding distracted driving and your behaviors regarding distracted driving. You will be asked to provide demographic information but will not be asked or required to provide personal identification information. The responses you provide are completely anonymous and cannot be connected with you at any time.

At the end of the survey, you will be directed to an external website which will collect your name and email address in order for the instructor to assign credit for participation in this study. If you choose to enter an email address in the external website form, you will receive a confirmation email for your records. If you choose to participate in the study and at the end of your participation you are not directed to the external website and/or do not receive a confirmation email, please contact [redacted] as soon as you encounter the technical difficulty.

Time required:
Approximately 20-30 minutes

Risks and Benefits:
There are minimal risks associated with this study. We do not anticipate that you will benefit directly by participating in this research.

Compensation:
You will receive Health Education Research Experience participation credit for this module in HSC 3102. The participation credit for this module is five (5) points of your total course grade.

Confidentiality:
We will not connect your name or email address to your responses. Your information will be assigned a code number. The PI, Co-PI, and Supervisor will not collect IP addresses, track IP addresses, or attach IP addresses to information. Your name will not be used in any report, presentation, or publication.

This survey contains a hidden item that collects information about your browser, browser version, operating system, screen resolution, flash version, java support version, and user agent from each device used to complete a survey. An example of the output created by Qualtrics for this item is below. (The output is the information that the researchers will be able to see when we analyze the results.)

Browser	Version	Operating System	Screen Resolution	Flash Version	Java Support	User Agent
Chrome	14.0.835.202	WOW64	1600x900	11.0.1	1	Mozilla/5.0 (Windows NT 6.1; WOW64) AppleWebKit/535.1 (KHTML, like Gecko) Chrome/14.0.835.202 Safari/535.1

This information identifies technical specifications of your device but cannot be used to identify you or your device.

Voluntary participation:
Your participation in this study is completely voluntary. There is no penalty for not participating. This survey contains several very sexually explicit terms, phrases, definitions, and questions that you may be uncomfortable reading or responding to. You can decline to answer any questions or quit taking the survey at any time without any penalty from your current or any future instructor. The survey software (Qualtrics) allows you to decline to answer any question to which you do not want to answer. The responses you provide are completely anonymous and cannot be connected with you at any time.

If you prefer to complete the journal entry for this module instead of this research, please close this window, return to the 3102 course website in Sakai and access the instructions for the module's journal entry located in the corresponding module page under the Course Materials tab.

Additional security:
The responses you provide are completely anonymous and cannot be connected with you at any time. The survey is delivered through Qualtrics. There is a minimal risk that security of any online data may be breached, but Qualtrics provides password protection (only the PI and Co-PI can access the data), hosts data on secure servers, and all results are firewall protected so it is highly unlikely that a security breach of the online data would occur or would result in an adverse consequence for you. The Qualtrics privacy statement can be located by clicking on the following link: http://www.qualtrics.com/privacy-statement

Right to withdraw from the study:
You have the right to withdraw from the study at anytime without consequence. You will still receive the participation credit (5 points) if you withdraw from the study before the conclusion of the survey. If you choose to participate in the study and at the end of your participation you are not directed to the external website, please contact [redacted] as soon as you encounter the technical difficulty.

Whom to contact if you have questions about the study:
[redacted]

Whom to contact about your rights as a research participant in the study:
IRB02 Office, [redacted], University of Florida, Gainesville, FL 32611-2250; [redacted].

Agreement:
I have read the procedure described above. I voluntarily agree to participate in the study.

- ◯ Begin survey (I consent to participating in this study)
- ◯ I do not want to participate in this study
- ◯ I have already participated in this study

Age Verification

Are you 18 years old or older?

- Yes
- No

In which of these age categories do you fall?

- Under 18
- 18-34
- 35 and older

Browser Meta Info

#EditSection, BrowserInfoExplanation#
Browser: **Chrome**
Version: **79.0.3945.88**
Operating System: **Windows NT 10.0**
Screen Resolution: **1280x1024**
Flash Version: **-1**
Java Support: **0**
User Agent: **Mozilla/5.0 (Windows NT 10.0; Win64; x64) AppleWebKit/537.36 (KHTML, like Gecko) Chrome/79.0.3945.88 Safari/537.36**

Driver Verification

Are you a licensed Driver?

- Yes
- No

Type of Phone Service

Do you use a cell phone and a landline phone for your personal telephone service or do you exclusively use a landline phone or cell phone?

- Use landline only
- Use cell phone only
- Use both landline phone and cell phone
- Decline to answer

General Driving Information

How often do you drive a motor vehicle, regardless of whether it is for work or for personal use?

- Almost every day (or more)
- Few days a week
- Few days a month
- Few days a year
- Never

- Other
  [ ]

Is the vehicle you drive most often a car, van, motorcycle, sport utility vehicle, pickup truck, or other type of truck?

- Car
- Van or minivan
- Motorcycle
- Pickup truck
- Sport Utility Vehicle
- Other truck
  [ ]
- Other
  [ ]
- Don't know

Perceptions of Enforcement

When you pass a driver stopped by the police IN THE DAYTIME, what do you think the stop was most likely for?

- Speeding
- Seat Belt Violation
- Drunk Driving
- Reckless Driving
- Cell phone use
- Texting or sending emails while driving
- Registration violation
- Other
  [ ]

When you pass a driver stopped by the police IN THE NIGHTTIME, what do you think the stop was most likely for?

- Speeding
- Seat Belt Violation
- Drunk Driving
- Reckless Driving
- Cell phone use
- Texting or sending emails while driving
- Registration violation
- Other
  [ ]

Ownership of Mobile Electronics

Do you CURRENTLY own any of the following devices?

	Yes	No	Mixed/Shared Use
A cell phone	○	○	○
A 'smartphone' such as a Droid, iPhone, or Blackberry	○	○	○
A pager or beeper	○	○	○
A portable music player, such as a CD player, iPod, or Zune	○	○	○
A portable navigation system, such as TomTom or Garmin	○	○	○
A navigation system built into the vehicle, such as OnStar or Sync	○	○	○
A laptop computer with cellular internet access, such as with Sprint or Verizon	○	○	○
A hands-free headset for your cell phone, such as one that plugs into the phone or works wirelessly	○	○	○

Frequency of Distracted Driving

For the next set of questions, you will be asked how often do you do different activities while you are driving.

How often do you talk to other passengers in the vehicle?

- ○ On all driving trips
- ○ On most driving trips
- ○ On some driving trips
- ○ Rarely
- ○ Never

How often do you eat or drink?

- ○ On all driving trips
- ○ On most driving trips
- ○ On some driving trips
- ○ Rarely
- ○ Never

How often do you make or accept phone calls?

- ○ On all driving trips
- ○ On most driving trips
- ○ On some driving trips
- ○ Rarely

- Never

How often do you read, such as a book, newspaper, iPad, or Kindle?

- On all driving trips
- On most driving trips
- On some driving trips
- Rarely
- Never

How often do you read emails or text messages?

- On all driving trips
- On most driving trips
- On some driving trips
- Rarely
- Never

How often do you send text messages or emails?

- On all driving trips
- On most driving trips
- On some driving trips
- Rarely
- Never

How often do you interact with children in the back seat?

- On all driving trips
- On most driving trips
- On some driving trips
- Rarely
- Never

How often do you do personal grooming, such as put on make-up, shave, or look at yourself in the mirror?

- On all driving trips
- On most driving trips
- On some driving trips
- Rarely
- Never

How often do you adjust the car radio?

- On all driving trips
- On most driving trips
- On some driving trips
- Rarely
- Never

How often do you change CDs, DVDs, or tapes?

- On all driving trips
- On most driving trips
- On some driving trips
- Rarely
- Never

How often do you use a portable music player with headphones on?

- On all driving trips
- On most driving trips
- On some driving trips
- Rarely
- Never
- Never, I do not own a portable music player.

How often do you use a portable music player with external speakers or with the car's speakers?

- On all driving trips
- On most driving trips
- On some driving trips
- Rarely
- Never
- Never, I do not own a portable music player.

How often do you use your Smartphone for driving directions?

- On all driving trips
- On most driving trips
- On some driving trips
- Rarely
- Never
- Never, I do not own a Smartphone.

How often do you use a navigation system for driving directions?

- On all driving trips
- On most driving trips
- On some driving trips
- Rarely
- Never
- Never, I do not own a navigation system.

Answering and Making Cell Phone Calls While Driving

When you RECEIVE a phone call while you are driving, how often do you ANSWER the call?

- On all driving trips
- On most driving trips
- On some driving trips
- Rarely
- Never

What are the reasons you are more likely to ANSWER a call while driving?

- I answer all calls
- Who is calling
- How important I think the call is
- Availability of the phone
- Call is work-related
- Call is personal or social
- Call is routine or expected
- Call is unexpected
- Call is from someone I know
- Call is from a number I don't recognize
- Non-stressful traffic conditions
- Good weather conditions
- Traveling at a low speed
- Time of day
- Boredom
- In need of directions or other information
- Personal safety
- If state law allows
- No police officers in sight
- Tired (talking keeps me awake)

- [ ] Other

When you answer a call while driving, do you USUALLY...

- ( ) Answer and continue to drive while completing the conversation
- ( ) Answer and promptly pull over to a safe location
- ( ) Answer and inform the caller you will call back later
- ( ) Pull over to a safe location first and then speak to the caller
- ( ) Hand the phone to a passenger to answer if you have one
- ( ) Other

Which of the following do you USUALLY do when you answer a call while driving?

- ( ) Hold the phone in your hand
- ( ) Squeeze the phone between your ear and shoulder
- ( ) Use a hands-free earpiece
- ( ) Use a built-in-car system (OnStar, Sync, or built-in Bluetooth)
- ( ) Use the cell phone's speakerphone feature
- ( ) Varies
- ( ) Other

When you are driving, how often are you willing to MAKE a phone call?

- ( ) On all driving trips
- ( ) On most driving trips
- ( ) On some driving trips
- ( ) Rarely
- ( ) Never

What are the reasons you are more likely to MAKE a call while driving?

- [ ] Who I'm calling
- [ ] How important/urgent I think the call is
- [ ] Availability of the phone
- [ ] Work-related
- [ ] Personal or social
- [ ] Non-stressful traffic conditions
- [ ] Good weather conditions
- [ ] Traveling at a low speed
- [ ] Time of day

- [ ] Boredom
- [ ] If I need directions or other information
- [ ] I think it's safe to call
- [ ] Personal Safety
- [ ] If state law permits
- [ ] No police officers in sight
- [ ] Report a traffic crash/emergency
- [ ] Report a medical emergency
- [ ] Tired (talking keeps me awake)
- [ ] Other ______

Which of the following ways do you usually MAKE a call while driving?

- [ ] Manual dialing
- [ ] Voice-dial (speaking a name or phone number)
- [ ] Speed dial or favorites
- [ ] Scroll through saved numbers and select
- [ ] Varies
- [ ] Other ______

How, if at all, would you say your driving is different when you are TALKING on the phone?

- [ ] No difference
- [ ] Drive slower
- [ ] Drive faster
- [ ] Change lanes more frequently
- [ ] Change lanes less frequently
- [ ] Avoid changing lanes altogether
- [ ] Apply the brakes suddenly
- [ ] Drift out of the lane or roadway
- [ ] Use turn signal less regularly
- [ ] Use turn signal more regularly
- [ ] Increase distance from lead vehicle
- [ ] Follow lead vehicle more closely
- [ ] Look in your rear or side view mirrors more frequently
- [ ] Look in your rear or side view mirrors less frequently
- [ ] Other ______

Is there any driving situation in which you would NEVER TALK on a phone while driving?

- [ ] When moving (not at stop signs or stop lights)
- [ ] On long trips
- [ ] On short trips
- [ ] Fast-moving traffic (freeway)
- [ ] Bumper-to-bumper traffic
- [ ] On an empty roadway
- [ ] Merging with traffic
- [ ] Bad weather
- [ ] Driving a familiar route
- [ ] Driving in unfamiliar area/roads
- [ ] Driving at nighttime
- [ ] Marked school zones
- [ ] Residential streets
- [ ] Parking lots
- [ ] With other adult passengers in the car
- [ ] With a baby or child on board
- [ ] Winding/curving roads
- [ ] Marked construction zones
- [ ] When I see a police officer
- [ ] Other ______

Texting or E-mailing While Driving

Do you ever SEND text messages or e-mails when you are driving?

- ( ) Yes
- ( ) No

If you SEND a text message or e-mail while driving, do you USUALLY...

- ( ) Continue to drive while completing the message
- ( ) Pull over to a safe location to send the message
- ( ) Hand the phone to a passenger to do your messaging
- ( ) Use a Voice Command feature (speech dictation)
- ( ) Other ______

What makes it more likely you will SEND a text message or e-mail while driving?

- [ ] Who I'm messaging
- [ ] How important I think the message is
- [ ] Work-related
- [ ] Personal or social
- [ ] Non-stressful traffic conditions
- [ ] Good weather conditions
- [ ] Traveling at a low speed
- [ ] Time of day
- [ ] Boredom
- [ ] In need of directions or other information
- [ ] I think it's safe to text
- [ ] Personal safety
- [ ] If state law permits
- [ ] If no police officers are in sight
- [ ] Report a traffic crash/emergency
- [ ] Report a medical emergency
- [ ] Tired (texting keeps me awake)
- [ ] Other

How would you say your driving is different when you are SENDING TEXT OR E-MAIL MESSAGES?

- [ ] No difference
- [ ] Drive slower
- [ ] Drive faster
- [ ] Change lanes more frequently
- [ ] Change lanes less frequently
- [ ] Avoid changing lanes altogether
- [ ] Apply the brakes suddenly
- [ ] Drift out of the lane or roadway
- [ ] Use turn signal less regularly
- [ ] Use turn signal more regularly
- [ ] Increase distance from lead vehicle
- [ ] Follow lead vehicle more closely
- [ ] Look in your rear or side view mirrors more frequently
- [ ] Look in your rear or side view mirrors less frequently
- [ ] Other

Is there any driving situation in which you would NEVER SEND a text or e-mail message while driving?

- ☐ When moving (not at stop signs or stop lights)
- ☐ On long trips
- ☐ On short trips
- ☐ Fast-moving traffic (freeway)
- ☐ Bumper-to-bumper traffic
- ☐ On an empty roadway
- ☐ Merging with traffic
- ☐ Bad weather
- ☐ Driving a familiar route
- ☐ Driving in unfamiliar area/roads
- ☐ Driving at nighttime
- ☐ Marked school zones
- ☐ Residential streets
- ☐ Parking lots
- ☐ With other adult passengers in the car
- ☐ With a baby or child on board
- ☐ Winding/curving roads
- ☐ Marked construction zones
- ☐ When I see a police officer
- ☐ Other ____________

Perceptions About Danger of Distractions

How many seconds do you believe a driver can take his or her eyes off the road before driving becomes significantly more dangerous?

- ○ Less than 1 second
- ○ 1-2 seconds
- ○ 3-4 seconds
- ○ 5-10 seconds
- ○ 10 seconds or more

How safe would you feel if you were a passenger riding in a car while your driver was doing the following:

	Very unsafe	Somewhat unsafe	A little less safe	Safe, no problem, would not pay any more attention
Talking to other passengers in the vehicle	○	○	○	○
Eating or drinking	○	○	○	○

	Very unsafe	Somewhat unsafe	A little less safe	Safe, no problem, would not pay any more attention
Talking on a cell phone while holding the phone	○	○	○	○
Talking on a cell phone with a hands-free device	○	○	○	○
Reading, such as a book, newspaper, or an iPad or Kindle	○	○	○	○
	Very unsafe	Somewhat unsafe	A little less safe	Safe, no problem, would not pay any more attention
Reading e-mails or text messages	○	○	○	○
Sending text messages or emails	○	○	○	○
Interacting with children in the back seat	○	○	○	○
Doing personal grooming, such as putting on makeup, shaving, looking in the mirror	○	○	○	○
Adjusting the car radio, tape, or CD player	○	○	○	○
	Very unsafe	Somewhat unsafe	A little less safe	Safe, no problem, would not pay any more attention
Singing along to a song on the radio	○	○	○	○
Using a laptop computer	○	○	○	○
Using a portable music player with headphones on	○	○	○	○
Manipulating a navigation system for driving directions	○	○	○	○
Watching a movie	○	○	○	○

Changes in Distracted Driving

In the past 30 days, has your frequency of making and receiving phone calls while driving increased, decreased, or stayed the same?

- ○ Increased
- ○ Decreased
- ○ Stayed the same
- ○ New driver (I haven't driven for more than 30 days)
- ○ Never used a phone while driving

What caused your frequency of making and receiving phone calls while driving to decrease?

- ☐ Increased awareness of safety
- ☐ Law that bans cell phone use
- ☐ Don't want to get a ticket

- Was in a crash
- Influence/pressure from others
- More long distance driving
- The weather
- Driving faster
- Other

In the past 30 days, has the frequency with which you send and receive text messages or emails while driving increased, decreased, or stayed the same?

- Increased
- Decreased
- Stayed the same
- New driver (I haven't driven for more than 30 days)
- Never used a phone while driving

What caused the frequency with which you send and receive text messages or e-mails while driving to decrease?

- Increased awareness of safety
- Law that bans cell phone use
- Don't want to get a ticket
- Was in a crash
- Influence/pressure from others
- More long distance driving
- The weather
- Driving faster
- Other

Distracted Driving Laws

Does Florida have a law banning talking on a handheld cell phone while driving?

- Yes
- Yes, probably
- No

Assume that over the next six months someone frequently TALKS on a handheld cell phone while driving. How likely do you think that person would be to receive a ticket for talking on a cell phone while driving?

- Very likely
- Somewhat likely
-

Somewhat unlikely

- ◯ Very unlikely

Does Florida have a law banning TEXTING OR E-MAILING while driving?

- ◯ Yes
- ◯ Yes, probably
- ◯ No

Assume that over the next six months someone frequently sends text messages or e-mails while driving. How likely do you think that person would be to receive a ticket for sending text messages or e-mails while driving?

- ◯ Very likely
- ◯ Somewhat likely
- ◯ Somewhat unlikely
- ◯ Very unlikely

Do you support a State law banning talking on a handheld cell phone while driving?

- ◯ Yes
- ◯ No

What do you think the fine should be for talking on a handheld cell phone while driving?

$ ______

Do you support a State law banning texting or e-mailing while driving?

- ◯ Yes
- ◯ No

What do you think the fine should be for sending text messages or e-mails while driving?

$ ______

Program Awareness

In the past 30 days, have you seen or heard of any special effort by police to ticket drivers in your community for using handheld cell phones while driving?

- ◯ Yes
- ◯ No

Where did you see or hear about that special effort?

- [ ] TV- advertisement/public service announcement
- [ ] TV-news
- [ ] Radio-advertisement/public service announcement
- [ ] Radio-news
- [ ] Online news/blog
- [ ] Internet ad/banner
- [ ] Social networking website (Facebook, MySpace, Twitter)
- [ ] Online video (YouTube, Google Video)
- [ ] Friend/relative
- [ ] Newspaper/magazine
- [ ] Witnessed enforcement activity
- [ ] Billboard/signs
- [ ] Educational program
- [ ] I'm a police officer/judge
- [ ] Direct contact by police officer
- [ ] Internet game
- [ ] Other ______

Were you personally stopped by police for using a handheld cell phone while driving in the past 30 days?

- ○ Yes
- ○ No

Did you receive a ticket or warning?

- ○ Yes- ticket for talking on a cell phone
- ○ Yes- warning for talking on a cell phone
- ○ Yes- ticket for texting or sending an e-mail
- ○ Yes- warning for texting or sending an e-mail
- ○ No

Exposure to Distracted Driving Crashes and Stories

Have you been involved in a crash or near-crash as a driver in the past year?

- ○ Yes- near-crash
- ○ Yes- crash
- ○ No

Were you using a cell phone at the time of the LAST [crash/near-crash] you were in?

- Yes- talking
- Yes- reading electronic text
- Yes- sending text message or e-mail
- No

Perceptions of and Responses to Other Distracted Drivers

What percentage of drivers do you believe at least occasionally TALK on a cell phone while driving?

What percentage of drivers do you believe at least occasionally SEND TEXT MESSAGES OR E-MAILS on a cell phone while driving?

Intervening as a Passenger

When riding as a passenger, how comfortable would you feel if your driver was TALKING on a cell phone while driving?

- Very comfortable- No problem
- Aware but uncomfortable
- Somewhat uncomfortable
- Uncomfortable
- Very uncomfortable

When riding as a passenger, how comfortable would you feel if your driver was SENDING TEXT MESSAGES OR E-MAILS while driving?

- Very comfortable- No problem
- Aware but uncomfortable
- Somewhat uncomfortable
- Uncomfortable
- Very uncomfortable

How likely are you to do or say something to your driver if they're TALKING on a handheld cell phone while driving?

- Very likely
- Somewhat likely
- Somewhat unlikely
- Very unlikely

How likely are you to do or say something to your driver if they're SENDING TEXT MESSAGES OR E-MAILS while driving?

- ○ Very likely
- ○ Somewhat likely
- ○ Somewhat unlikely
- ○ Very unlikely

Demographic Questions (NHTSA)

What is your age?

[ ]

Where do you currently live?

- ○ On campus dormitory
- ○ Off-campus dormitory
- ○ Apartment
- ○ House
- ○ Other

Including yourself, how many persons, age 16 or older, are living in your household at least half of the time or consider it their primary residence?

[ ]

How many children age 15 or younger are living in your household at least half of the time or consider it their primary residence?

[ ]

Do you consider yourself to be Hispanic or Latino?

- ○ Yes
- ○ No

Which of the following racial categories describe you? You may select more than one.

- ☐ American Indian or Alaska Native
- ☐ Asian
- ☐ Black or African American
- ☐ Native Hawaiian or other Pacific Islander
- ☐ White

What is the highest level of education you have completed?

- Some high school
- High school/GED
- Some college
- College
- Graduate or professional school

How many landline telephone numbers do you have in your household?

How many separate cell phone numbers do you have in your household?

What is your approximate household income?

- Less than $10,000
- $10,000-$14,999
- $15,000-$24,999
- $25,000-$49,999
- $50,000-$99,999
- $100,000-$149,999
- $150,000-$199,999
- $200,000 or more
- Decline to answer

What is your sex?

- Male
- Female
