# Supplementary material for: The Health Education Research Experience (HERE) program metadata dataset
Source: Data Brief. 2020 Jan 25;29:105180. doi: 10.1016/j.dib.2020.105180 (PMC7100622; doi:10.1016/j.dib.2020.105180)
Supplement: Multimedia component 12 [file mmc12.pdf]

Technology, Health, and College Students

Informed Consent
Protocol Title: Technology, Health, and College Students
Please read this consent document carefully before you decide to participate in this study.

Purpose of the research study:
The purpose of this study is to examine University of Florida students' use of technology to search for and access health information. This research will supplement the current Health Education literature. We are also interested in how you complete this survey (e.g. on your computer, your phone, or a tablet computer like an iPad). As such, the survey program, Qualtrics, will collect technical information addressed in the Confidentiality Section below.

Role of Research in HSC 3102:
One of the primary responsibilities of Certified Health Education Specialists is to *Conduct Evaluation and Research Related to Health Education*. As such, one of the goals of HSC 3102 – Personal and Family Health -- is to familiarize you with the research process in health education. To familiarize you with the research process in health education, we have created online surveys and introspective journal entries related to the content in each module.

Earning Health Education Research Experience Points:
This module includes a survey AND a journal entry. For this module, you may choose to participate in EITHER activity to receive your Health Education Research Experience points (5 points). Deadlines for the this module's survey participation or journal entry are listed in the Sakai course website and correspond with the deadline for completing this module.

What you will be asked to do in the study:
You will be asked to take a 35-item questionnaire online through Qualtrics. In this study you will be asked how you search for health information and how believable different sources are. You will be asked to provide demographic information but will not be asked or required to provide personal identification information.

At the end of the survey, you will be directed to an external website which will collect your name and email address in order for the instructor to assign credit for participation in this study. If you choose to enter an email address in the external website form, you will receive a confirmation email for your records. If you choose to participate in the study and at the end of your participation you are not directed to the external website and/or do not receive a confirmation email, please contact [redacted] as soon as you encounter the technical difficulty.

Your name will not be connected at any time with your survey responses. The instructor will not be able to view your responses or connect your name with your responses.

Confidentiality:
We will not connect your name or email address to your responses. Your information will be assigned a code number. The PI, Co-PI, and Supervisor will not collect IP addresses, track IP addresses, or attach IP addresses to information. Your name will not be used in any report, presentation, or publication.

This survey contains a hidden item that collects information about your browser, browser version, operating system, screen resolution, flash version, java support version, and user agent from each device used to complete a survey. An example of the output created by Qualtrics for this item is below. (The output is the information that the researchers will be able to see when we analyze the results.)

Browser	Version	Operating System	Screen Resolution	Flash Version	Java Support	User Agent
Chrome	14.0.835.202	WOW64	1600x900	11.0.1	1	Mozilla/5.0 (Windows NT 6.1; WOW64) AppleWebKit/535.1 (KHTML, like Gecko) Chrome/14.0.835.202 Safari/535.1

This information identifies technical specifications of your device but cannot be used to identify you or your device.

Additional security:
The responses you provide are completely anonymous and cannot be connected with you at any time. The survey is delivered through Qualtrics. There is a minimal risk that security of anonlinene data may be breached, but Qualtrics provides password protection (only the PI and Co-PI can access the data), hosts data on secure servers, and all results are firewall protected so it is highly unlikely that a security breach of the online data would occur or would result in an adverse consequence for you. The Qualtrics privacy statement can be located by clicking on the following link: http://www.qualtrics.com/privacy-statement

Time required:
Approximately 20-30 minutes

Risks and Benefits:
There are minimal risks associated with this study. We do not anticipate that you will benefit directly by participating in this research.

Compensation:
You will receive Health Education Research Experience participation credit for this module in HSC 3102. The participation credit for this module is five (5) points of your total course grade.

Voluntary participation:
Your participation in this study is completely voluntary. There is no penalty for not participating. If you would prefer to complete the journal entry for this module instead of this research, please close this window, return to the 3102 course website in Sakai and access the instructions for the module's journal entry located in the corresponding module page under the Course Materials tab.

Right to withdraw from the study:
You have the right to withdraw from the study at anytime without consequence. You will still receive the participation credit (5 points) if you withdraw from the study before the conclusion of the survey.

Whom to contact if you have questions about the study:
[redacted]

Whom to contact about your rights as a research participant in the study:
IRB02 Office, [redacted], University of Florida, Gainesville, FL 32611-2250; [redacted]

Agreement:
I have read the procedure described above. I voluntarily agree to participate in the procedure.

- ○ I consent (I want to participate in this study).
- ○ I do not consent (I do not want to participate in this study).

Health Information Sources

Browser Meta Info

#EditSection, BrowserInfoExplanation#
Browser: **Chrome**
Version: **79.0.3945.88**
Operating System: **Windows NT 10.0**
Screen Resolution: **1280x1024**
Flash Version: **-1**
Java Support: **0**
User Agent: **Mozilla/5.0 (Windows NT 10.0; Win64; x64) AppleWebKit/537.36 (KHTML, like Gecko) Chrome/79.0.3945.88 Safari/537.36**

Which source do you FIRST consult when searching for health information?

- Campus newspaper
- Friend (who is not also a health educator, nurse, or physician)
- Health Educator (who is not also your parent, relative or a friend)
- Internet
- Magazine
- Mobile App
- Nurse (who is not also your parent, relative, or friend)
- Parent(s)
- Physician (who is not also your parent, relative, or friend)
- Professor (who is not also your parent, relative, or friend)
- A relative other than your parent
- Television
- It depends on what kind of information I am searching for.
- Other

To what extent do you agree or disagree with the following statement: I know how to search for health information on the Internet.

- Strongly agree
- Agree
- Disagree
- Strongly Disagree

To what extent do you believe the following sources of health information?

	Unbelievable	Somewhat Unbelievable	Somewhat Believable	Believable
Campus newspaper				
Family members				
Friends				
Health educators				
Internet				
Magazines				
Mobile apps				
Student Health Care Center				

Devices and Apps

Do you currently own any of the following?

	Yes	No

	Yes	No
Cell phone with Internet capability (smart phone)	○	○
Cell phone without Internet capability	○	○
Desktop computer	○	○
Electronic book reader (e.g. Nook, Kindle)	○	○
Game console (e.g. X-Box, PlayStation, Nintendo Wii)	○	○
	Yes	No
Laptop computer	○	○
mp3 player (e.g. iPod)	○	○
Netbook computer	○	○
Portable gaming device (e.g. Sony PSP, Nintendo 3DS)	○	○
Tablet computer (e.g. iPad)	○	○

What is the brand of your Internet-enabled cell phone?

- ○ Apple iPhone
- ○ Blackberry
- ○ Motorola
- ○ Palm
- ○ Samsung
- ○ LG
- ○ HTC
- ○ Other

Do you currently have any health-related software applications (apps) on your cell phone?

- ○ Yes
- ○ No
- ○ I'm not sure

Do you currently have any health-related apps on your tablet computer?

- ○ Yes
- ○ No
- ○ I'm not sure

Do you have any of the following apps on your cell phone or tablet computer?

	Yes	No	I'm not sure	I don't use apps
Ab Workouts	○	○	○	○

	Yes	No	I'm not sure	I don't use apps
Calorie Counter				
DroidFit				
Health & Fitness Magazine				
iFitness				
iTriage				
	Yes	No	I'm not sure	I don't use apps
LiveStrong				
Lose It				
MyFitnessPal				
Nike + GPS				
Nike Training Club				
RunKeeper				
	Yes	No	I'm not sure	I don't use apps
RunTracker				
WebMD				
Weight Watchers				
Other				

Within the last month, how often did you use health information from an app to inform your health decisions?

- Never
- Once
- 2-3 Times a Month
- Once a Week
- 2-3 Times a Week
- Daily

Block 4

Do you have access to a high-speed Internet connection at your current residence?

- Yes
- No
- I'm not sure

Have you ever used the Internet to search for health information?

- Yes
- No

If you have used the Internet to search for health information, which website do you use FIRST when you search for health information on the Internet?

- Ask.com
- Centers for Disease Control and Prevention (CDC)
- CNN
- Fox
- goaskalice
- Google
- Mayo Clinic
- National Institutes of Health (NIH)
- WebMD
- Wikipedia
- World Health Organization (WHO)
- Yahoo Health
- Other

Within the last 30 days, have you searched for health information on the Internet related to your own health?

- Yes
- No
- I'm not sure

Within the last 30 days, have you searched for health information on the Internet related to someone else's health?

- Yes
- No
- I'm not sure

Within the last 30 days, have you used your cell phone to search the Internet for health-related information?

- Yes
- No
- I'm not sure

How frequently did you use your cell phone to search the Internet for health-related information?

- Never
- Less than Once a Month
- Once a Month
- 2-3 Times a Month
- Once a Week
- 2-3 Times a Week
- Daily

Do you have an unlimited text message/SMS plan?

- Yes
- No
- I don't know

Approximately how many text/SMS messages do you send per day?

- 0-10
- 11-20
- 21-50
- More than 50
- I don't know

How many text/SMS messages do you receive per day?

- 0-10
- 11-20
- 21-50
- More than 50
- I don't know

Within the last 30 days, did you receive any health information text messages?

- Yes
- No
- I'm not sure

Within the last 30 days have you used the Internet to search for information about any of the following topics?

	Yes	No	I'm not sure
Alcohol			
Alternative treatments			
Cancer			
Environmental health hazards			
Exercise/ fitness			
Experimental treatments			
Health insurance			
	Yes	No	I'm not sure
Heart conditions			
Illegal drugs			
Injury prevention			
Medical procedures			

	Yes	No	I'm not sure
Medication side effects	○	○	○
Mental health (including anxiety, depression, and stress)	○	○	○
Nutrition	○	○	○
	Yes	No	I'm not sure
Occupational health and safety	○	○	○
Oral health	○	○	○
Over-the-counter medications	○	○	○
Prescription medications	○	○	○
Sexual health	○	○	○
Sleep health	○	○	○
Smoking cessation (how to quit smoking)	○	○	○
	Yes	No	I'm not sure
Stroke	○	○	○
Vaccinations	○	○	○
Violence prevention	○	○	○
Vision health	○	○	○
Other ______	○	○	○

Social Media

Please tell us if you have ever used the Internet to do any of the following things:
Do you ever use the Internet to...

	Yes	No
Create or work on an on-line journal or blog	○	○
Use a social networking site (e.g. MySpace, Facebook, LinkedIn, etc)	○	○
Visit virtual world, (e.g. Sims, Second Life)	○	○
Post comments to an on-line news website, blog, or photo/video site	○	○

On which social media sites do you have a profile or account?

	Yes	No
Blogger	○	○
Facebook	○	○
FlickR	○	○
Foursquare	○	○
Google Plus+	○	○
	Yes	No
MySpace	○	○

	Yes	No
LinkedIn	○	○
LiveJournal	○	○
Second Life	○	○
Twitter	○	○
	Yes	No
Ning	○	○
YouTube	○	○
Other: Please List ____	○	○
I do not have any social media profiles	○	○

On how many social networking websites do you currently have a profile?

- ○ 1
- ○ 2
- ○ 3
- ○ 4
- ○ 5 or more
- ○ I don't have a social network profile.

Perceived Health

How do you rate your current quality of life?

- ○ Excellent
- ○ Very Good
- ○ Good
- ○ Fair
- ○ Poor

How do you rate your current physical health (including physical illness and injury)?

- ○ Excellent
- ○ Very Good
- ○ Good
- ○ Fair
- ○ Poor

How do you rate your current mental health (including stress, depression, and problems with emotions)?

- ○ Excellent
- ○ Very Good

- Good
- Fair
- Poor

Demographics

What is your age?

What is your classification at the University of Florida?

- Freshman
- Sophomore
- Junior
- Senior
- Graduate Student
- Professional Student
- Non-degree seeking student
- I am not a student at the University of Florida

In which college is your current major?

- College of Agricultural and Life Sciences
- College of Business Administration
- College of Dentistry
- College of Design, Construction, and Planning
- College of Education
- College of Engineering
- College of Fine Arts
- College of Health and Human Performance
- College of Journalism and Communications
- College of Law
- College of Liberal Arts and Sciences
- College of Medicine
- College of Nursing
- College of Pharmacy
- College of Public Health and Health Professions
- College of Veterinary Medicine

How would you classify your sexual orientation?

- Asexual
- Bisexual/Bi
- Heterosexual/Straight
- Homosexual/Gay/Lesbian/Queer
- Unsure
- Decline to answer

What is your current relationship status?

- Married
- In a committed relationship (with a steady partner)
- Single (not dating)
- Dating
- Divorced
- Widowed
- Separated
- Other

What is your current health insurance status?

- I am covered under my parents' insurance.
- I have health insurance through my job not associated with the University of Florida.
- I have health insurance through my spouse.
- I have health insurance through the University of Florida.
- I am not insured.
- I don't know.

Where do you currently live?

- On campus dormitory
- Off-campus dormitory
- Apartment
- House
- Other

What is your approximate grade point average (one decimal place)?

What is your sex?

- Male

- ○ Female

Are you a member of a social fraternity or sorority?

- ○ Yes
- ○ No
- ○ I am in the process of pledging/rushing/recruitment this semester

Have you ever served on active duty in the U.S. Armed Forces, military Reserves, or National Guard? *Active Duty does not include training for the Reserves or National Guard, but DOES include activation, for example, for the Persian Gulf War.*

- ○ Yes, now on active duty
- ○ Yes, on active duty during the last 12 months, but not now
- ○ Yes, on active duty in the past, but not during the last 12 months
- ○ No, training for Reserves or National Guard only
- ○ No, never served in the military

What is your race? (One or more categories may be selected)

- ☐ White
- ☐ Black or African American
- ☐ American Indian or Alaska Native
- ☐ Asian Indian
- ☐ Chinese
- ☐ Filipino
- ☐ Japanese
- ☐ Korean
- ☐ Vietnamese
- ☐ Other Asian
- ☐ Native Hawaiian
- ☐ Guamanian or Chamorro
- ☐ Samoan
- ☐ Other Pacific Islander

Are you Hispanic, Latino/a, or Spanish Origin? (One or more categories may be selected)

- ☐ No, not of Hispanic, Latino/a, or Spanish origin
- ☐ Yes, Mexican, Mexican American, Chicano/a
- ☐ Yes, Puerto Rican
- ☐ Yes, Cuban
- ☐ Yes, Another Hispanic, Latino/a, or Spanish origin

Do you have any comments regarding this survey or how we can improve this survey for future participants?
