# Supplementary material for: The Health Education Research Experience (HERE) program metadata dataset
Source: Data Brief. 2020 Jan 25;29:105180. doi: 10.1016/j.dib.2020.105180 (PMC7100622; doi:10.1016/j.dib.2020.105180)
Supplement: Multimedia component 13 [file mmc13.pdf]

The Field of Health Education

Informed Consent
Protocol Title: The Field of Health Education
Please read this consent document carefully before you decide to participate in this study.

Purpose of the research study:
The purpose of this study is to examine University of Florida students' knowledge of the field of Health Education. This research will supplement the current Health Education literature. We are also interested in how you complete this survey (e.g. on your computer, your phone, or a tablet computer like an IPad). As such, the survey program, Qualtrics, will collect technical information addressed in the Confidentiality Section below.

Role of Research in HSC 3102:
One of the primary responsibilities of Certified Health Education Specialists is to *Conduct Evaluation and Research Related to Health Education*. As such, one of the goals of HSC 3102 – Personal and Family Health -- is to familiarize you with the research process in health education. To familiarize you with the research process in health education, we have created online surveys and introspective journal entries related to the content in each module.

Earning Health Education Research Experience Points:
This module includes a survey AND a journal entry. For this module, you may choose to participate in EITHER activity to receive your Health Education Research Experience points (5 points). Deadlines for this module's survey participation or journal entry are listed in the Sakai course website and correspond with the deadline for completing this module.

What you will be asked to do in the study:
You will be asked to take a 35-item questionnaire online through Qualtrics. In this study you will be asked your opinions about the field of health education and the work of health education specialists. You will be asked to provide demographic information but will not be asked or required to provide personal identification information.

At the end of the survey, you will be directed to an external website which will collect your name and email address in order for the instructor to assign credit for participation in this study. If you choose to enter an email address in the external website form, you will receive a confirmation email for your records. If you choose to participate in the study and at the end of your participation you are not directed to the external website and/or do not receive a confirmation email, please contact [redacted] as soon as you encounter the technical difficulty.

Confidentiality:
We will not connect your name or email address to your responses. Your information will be assigned a code number. The PI, Co-PI, and Supervisor will not log IP addresses, track IP addresses, or attach IP addresses to information. Your name will not be used in any report, presentation, or publication.

This survey contains a hidden item that collects information about your browser, browser version, operating system, screen resolution, flash version, java support version, and user agent from each device used to complete a survey. An example of the output created by Qualtrics for this item is below. (The output is the information that the researchers will be able to see when we analyze the results.)

Browser	Version	Operating System	Screen Resolution	Flash Version	Java Support	User Agent
Chrome	14.0.835.202	WOW64	1600x900	11.0.1	1	Mozilla/5.0 (Windows NT 6.1; WOW64) AppleWebKit/535.1 (KHTML, like Gecko) Chrome/14.0.835.202 Safari/535.1

This information identifies technical specifications of your device but cannot be used to identify you or your device.

Additional security:
The responses you provide are completely anonymous and cannot be connected with you at any time. The survey is delivered through Qualtrics. There is a minimal risk that security of any online data may be breached, but Qualtrics provides password protection (only the PI and Co-PI can access the data), hosts data on secure servers, and all results are firewall protected so it is highly unlikely that a security breach of the online data would occur or would result in an adverse consequence for you. The Qualtrics privacy statement can be located by clicking on the following link: http://www.qualtrics.com/privacy-statement

Time required:
Approximately 20-30 minutes

Risks and Benefits:
There are minimal risks associated with this study. We do not anticipate that you will benefit directly by participating in this research.

Compensation:
You will receive Health Education Research Experience participation credit for this module in HSC 3102. The participation credit for this module is five (5) points of your total course grade.

Voluntary participation:
Your participation in this study is completely voluntary. There is no penalty for not participating. If you prefer to complete the journal entry for this module instead of this research, please close this window, return to the 3102 course website in Sakai and access the instructions for the module's journal entry located in the corresponding module page under the Course Materials tab.

Right to withdraw from the study:
You have the right to withdraw from the study at anytime without consequence. You will still receive the participation credit (5 points) if you withdraw from the study before the conclusion of the survey. If you choose to participate in the study and at the end of your participation you are not directed to the external website, please contact [redacted] as soon as you encounter the technical difficulty.

Whom to contact if you have questions about the study:
[redacted]

[redacted]

Whom to contact about your rights as a research participant in the study:
IRB02 Office, [redacted], University of Florida, Gainesville, FL 32611-2250; [redacted]

Agreement:
I have read the procedure described above. I voluntarily agree to participate in the study.

- ○ I consent (I want to participate in this study).
- ○ I do not consent (I do not want to participate in this study).
- ○ I have already participated in this study.

The Field of Health Education

Browser Meta Info

#EditSection, BrowserInfoExplanation#
Browser: **Chrome**
Version: **79.0.3945.88**
Operating System: **Windows NT 10.0**
Screen Resolution: **1280x1024**
Flash Version: **-1**
Java Support: **0**
User Agent: **Mozilla/5.0 (Windows NT 10.0; Win64; x64) AppleWebKit/537.36 (KHTML, like Gecko) Chrome/79.0.3945.88 Safari/537.36**

A health education specialist: assesses individual and community needs for health education.

- ○ Agree
- ○ Disagree
- ○ I am not sure.

A health education specialist: plans health education strategies, interventions, and programs.

- ○ Agree
- ○ Disagree
- ○ I am not sure.

A health education specialist: prescribes medication as part of health education programs.

- ○ Agree
- ○ Disagree
- ○ I am not sure.

A health education specialist: dispenses medication as part of health education programs.

- ○ Agree

- Disagree
- I am not sure.

A health education specialist: implements health education strategies, interventions, and programs.

- Agree
- Disagree
- I am not sure.

A health education specialist: conducts evaluation and research related to health education.

- Agree
- Disagree
- I am not sure.

A health education specialist: administers health education strategies, interventions, and programs.

- Agree
- Disagree
- I am not sure.

A health education specialist: diagnoses diseases and conditions in health education settings.

- Agree
- Disagree
- I am not sure.

A health education specialist: serves as a health education resource person.

- Agree
- Disagree
- I am not sure.

A health education specialist: only teaches health in elementary schools.

- Agree
- Disagree
- I am not sure.

A health education specialist: only teaches health in middle/intermediate schools.

- Agree
- Disagree
- I am not sure.

A health education specialist: only teaches health in high schools.

- Agree
- Disagree
- I am not sure.

A health education specialist: communicates and advocates for health and health education.

- Agree
- Disagree
- I am not sure.

Does the University of Florida employ health education specialists?

- Yes
- No
- I am not sure.

Does a unified health education professional organization exist?

- Yes
- No
- I am not sure.

Is it possible to earn a bachelor's degree through the Department of Health Education and Behavior at the University of Florida?

- Yes
- No
- I am not sure.

Is it possible to earn a master's degree through the Department of Health Education and Behavior at the University of Florida?

- Yes
- No
- I am not sure.

Is it possible to earn a doctoral (Ph.D.) degree through the Department of Health Education and Behavior at the University of Florida?

- Yes
- No
- I am not sure.

Does a health education specialist need a state license to practice health education?

- Yes
- No
- I am not sure.

Does a health education specialist need a national license to practice health education?

- Yes
- No
- I am not sure.

The field of health education has a code of ethics.

- Yes it does.
- No, it does not.
- I am not sure.

An individual must be a Certified Health Education Specialist (CHES) to be considered a health education specialist.

- Yes
- No
- I am not sure.

What is your sex?

- Male
- Female

What is your classification at the University of Florida?

- Freshman
- Sophomore
- Junior
- Senior
- Graduate Student
- Professional Student
- Non-degree seeking student
- I am not a student at the University of Florida

Are you a Health Education & Behavior major at the University of Florida?

- Yes
- No

How many courses in Health Education and Behavior have you completed at the University of Florida?

- 0
- 1
- 2
- 3
- 4
- 5
- 6
- 7
- 8
- 9
- 10
- More than 10
- I don't know

In which college is your current major?

- College of Agricultural and Life Sciences
- College of Business Administration
- College of Dentistry
- College of Design, Construction, and Planning
- College of Education
- College of Engineering
- College of Fine Arts
- College of Health and Human Performance
- College of Journalism and Communications
- College of Law
- College of Liberal Arts and Sciences
- College of Medicine
- College of Nursing
- College of Pharmacy
- College of Public Health and Health Professions
- College of Veterinary Medicine

Are you a member of a social fraternity or sorority?

- Yes
- No
- I am in the process of pledging/rushing/recruitment this semester

Have you ever served on active duty in the U.S. Armed Forces, military Reserves, or National Guard? *Active Duty does not include training for the Reserves or National Guard, but DOES include activation, for example, for the Persian Gulf War.*

- ○ Yes, now on active duty
- ○ Yes, on active duty during the last 12 months, but not now
- ○ Yes, on active duty in the past, but not during the last 12 months
- ○ No, training for Reserves or National Guard only
- ○ No, never served in the military

What is your race? (One or more categories may be selected)

- ☐ White
- ☐ Black or African American
- ☐ American Indian or Alaska Native
- ☐ Asian Indian
- ☐ Chinese
- ☐ Filipino
- ☐ Japanese
- ☐ Korean
- ☐ Vietnamese
- ☐ Other Asian
- ☐ Native Hawaiian
- ☐ Guamanian or Chamorro
- ☐ Samoan
- ☐ Other Pacific Islander

Are you Hispanic, Latino/a, or Spanish Origin? (One or more categories may be selected)

- ☐ No, not of Hispanic, Latino/a, or Spanish origin
- ☐ Yes, Mexican, Mexican American, Chicano/a
- ☐ Yes, Puerto Rican
- ☐ Yes, Cuban
- ☐ Yes, Another Hispanic, Latino/a, or Spanish origin

Do you have any comments regarding this survey or how we can improve this survey for future participants?

Powered by Qualtrics
